# Templation and Concentration Drive Conversion Between a Fe^II^_12_L_12_ Pseudoicosahedron, a Fe^II^_4_L_4_ Tetrahedron, and a Fe^II^_2_L_3_ Helicate

**DOI:** 10.1021/jacs.1c11536

**Published:** 2022-01-11

**Authors:** Dawei Zhang, Quan Gan, Alex J. Plajer, Roy Lavendomme, Tanya K. Ronson, Zifei Lu, Jesper D. Jensen, Bo W. Laursen, Jonathan R. Nitschke

**Affiliations:** † Shanghai Key Laboratory of Green Chemistry and Chemical Processes, School of Chemistry and Molecular Engineering, East China Normal University, Shanghai 200062, People’s Republic of China; ‡ Department of Chemistry, University of Cambridge, Lensfield Road, Cambridge, CB2 1EW, United Kingdom; # Hubei Key Laboratory of Bioinorganic Chemistry & Materia Medica, School of Chemistry and Chemical Engineering, Huazhong University of Science and Technology, Wuhan 430074, People’s Republic of China; ⊥ Oxford Chemistry, Chemical Research Laboratory, 12 Mansfield Road, Oxford, OX1 3TA, U.K.; § COMOC—Center for Ordered Materials, Organometallics and Catalysis, Department of Chemistry, Ghent University, Krijgslaan 281-S3, 9000 Ghent, Belgium; ∥ Department of Chemistry & Nano-Science Center, University of Copenhagen, Universitetsparken 5, 2100, Copenhagen, Denmark

## Abstract

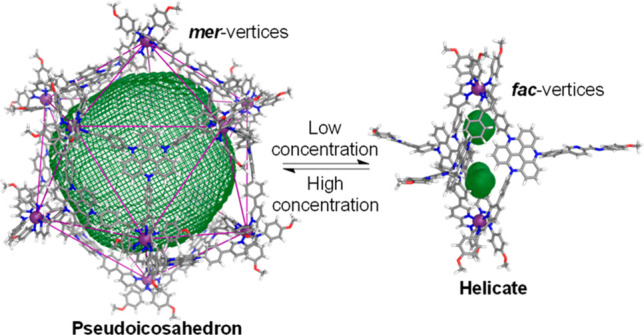

We report the construction of three structurally distinct self-assembled architectures: Fe^II^_12_L_12_ pseudoicosahedron **1**, Fe^II^_2_L_3_ helicate **2**, and Fe^II^_4_L_4_ tetrahedron **3**, formed from a single triazatriangulenium subcomponent **A** under different reaction conditions. Pseudoicosahedral capsule **1** is the largest formed through subcomponent self-assembly to date, with an outer-sphere diameter of 5.4 nm and a cavity volume of 15 nm^3^. The outcome of self-assembly depended upon concentration, where the formation of pseudoicosahedron **1** was favored at higher concentrations, while helicate **2** exclusively formed at lower concentrations. The conversion of pseudoicosahedron **1** or helicate **2** into tetrahedron **3** occurred following the addition of a CB_11_H_12_^–^ or B_12_F_12_^2–^ template.

Coordination-driven self-assembly is an efficient tool for the construction of polyhedral metal–organic complexes,^1^ the cavities of which have proven useful in a range of applications, including molecular recognition,^2^ stereochemical sensing,^3^ chemical separation,^4^ stabilization of reactive species,^5^ and catalysis.^6^ The strategy of subcomponent self-assembly,^7^ involving the formation of structures containing multiple N→metal and C=N linkages during the same overall process, allows the preparation of a variety of capsules with different shapes and cavity sizes, which bind many different guests.^7a^

An attractive goal is the construction of large self-assembled architectures^8^ that resemble the icosahedral structures adopted by some protein cages.^9^ As in the cases of icosahedral viral capsids assembled from multiple copies of a single protein subunit, self-assembly can allow the construction of larger architectures from much smaller components. The large internal voids of capsules with a sufficient degree of cavity enclosure may be suitable for binding large substrates,^10^ enabling synthetic encapsulants to approach the complex functions exhibited by biological systems.

In analogy to the structural changes of biological molecules,^11^ designing stimuli-responsive transformations within systems of discrete self-assembled container molecules is an important challenge in supramolecular chemistry.^12^ Such transformations may lead to functions that include guest uptake and release,^13^ chemical purification,^14^ reagent storage,^15^ and drug delivery.^16^ Various stimuli, such as light,^13b,17^ pH,^18^ temperature,^19^ solvent,^2c,20^ concentration,^21^ or additional chemical signals,^22^ have been employed to trigger transformation processes that lead to structural conversions. Supramolecular transformations involving multiple different structure types based upon a single ligand and metal ion remain rare,^23^ however.

Here, we report the preparation of three different architectures, a Fe^II^_12_L_12_ pseudoicosahedron, a Fe^II^_2_L_3_ helicate, and a Fe^II^_4_L_4_ tetrahedron, assembled from the same triazatriangulenium (TATA) subcomponent under different reaction conditions. Changes in ligand concentration or the addition of template anions triggered complete conversions between these assemblies.

Subcomponent **A** (Figure 1) was prepared following our previously reported procedure.^24^ We first explored its self-assembly at a concentration of 4.4 mM in acetonitrile. The reaction of subcomponents **A** (1 equiv) and *p*-anisidine (3 equiv) with Fe(BF_4_)_2_ (1 equiv) in acetonitrile at 70 °C resulted in the formation of the very large architecture **1**. ESI-MS showed a series of sharp peaks (Figure 2b), corresponding to charge states from 18+ to 11+, all of which were consistent with a Fe^II^_12_L_12_ composition.

**Figure 1:**
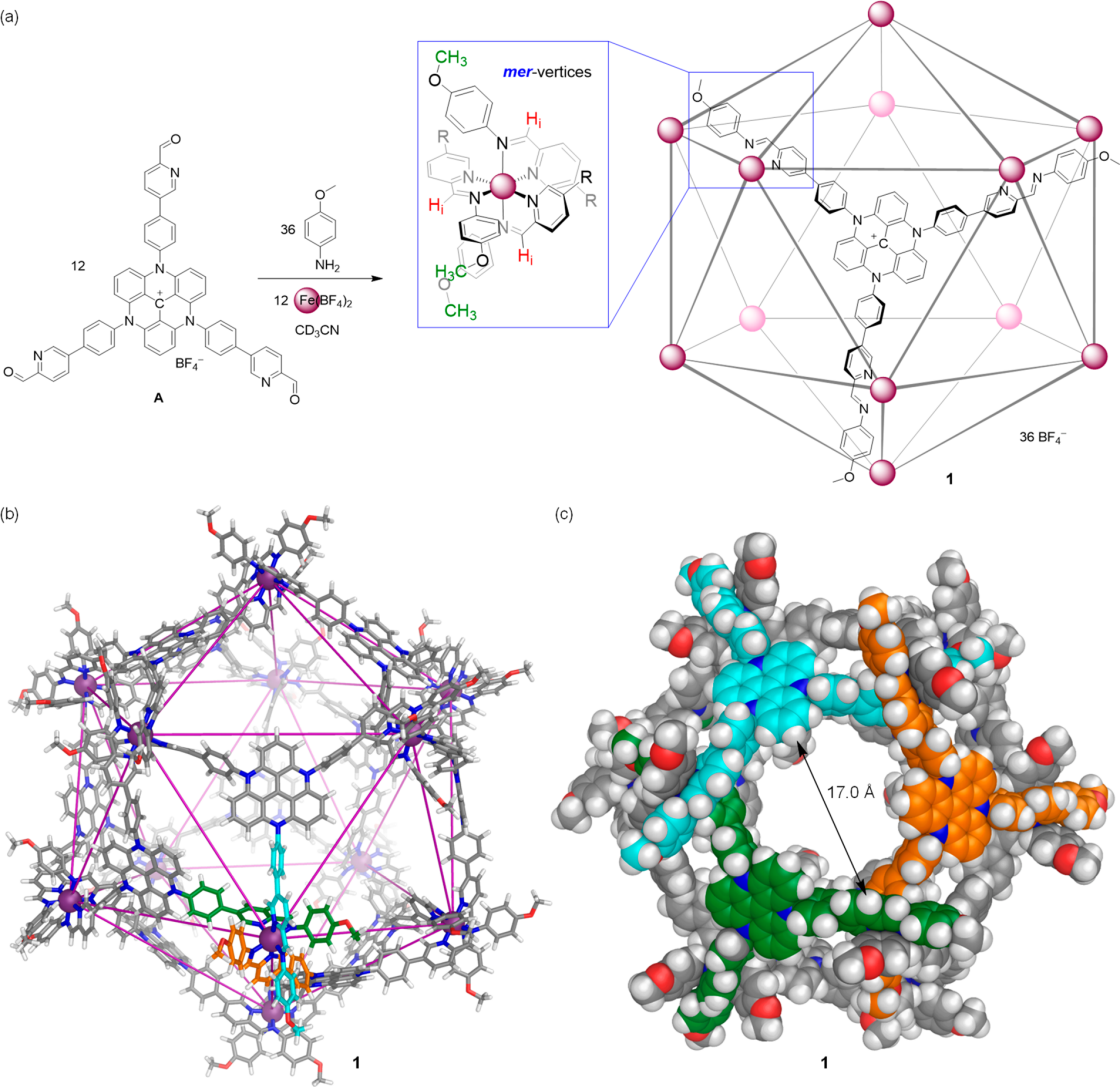
(a) Subcomponent self-assembly of Fe^II^_12_L_12_ pseudoicosahedron **1**, with cutout showing the *meridional* metal coordination geometry. (b) PM7-optimized molecular model of pseudoicosahedron **1**. Carbon atoms for the three distinct ligand arms about one of the *mer*-vertices have been colored cyan, green, and orange, respectively. (c) Model of **1** in space-filling mode, to show the porosity. Carbon atoms of the three triazatriangulenium ligands surrounding a large pore of **1** have been colored cyan, green, and orange, respectively.

**Figure 2:**
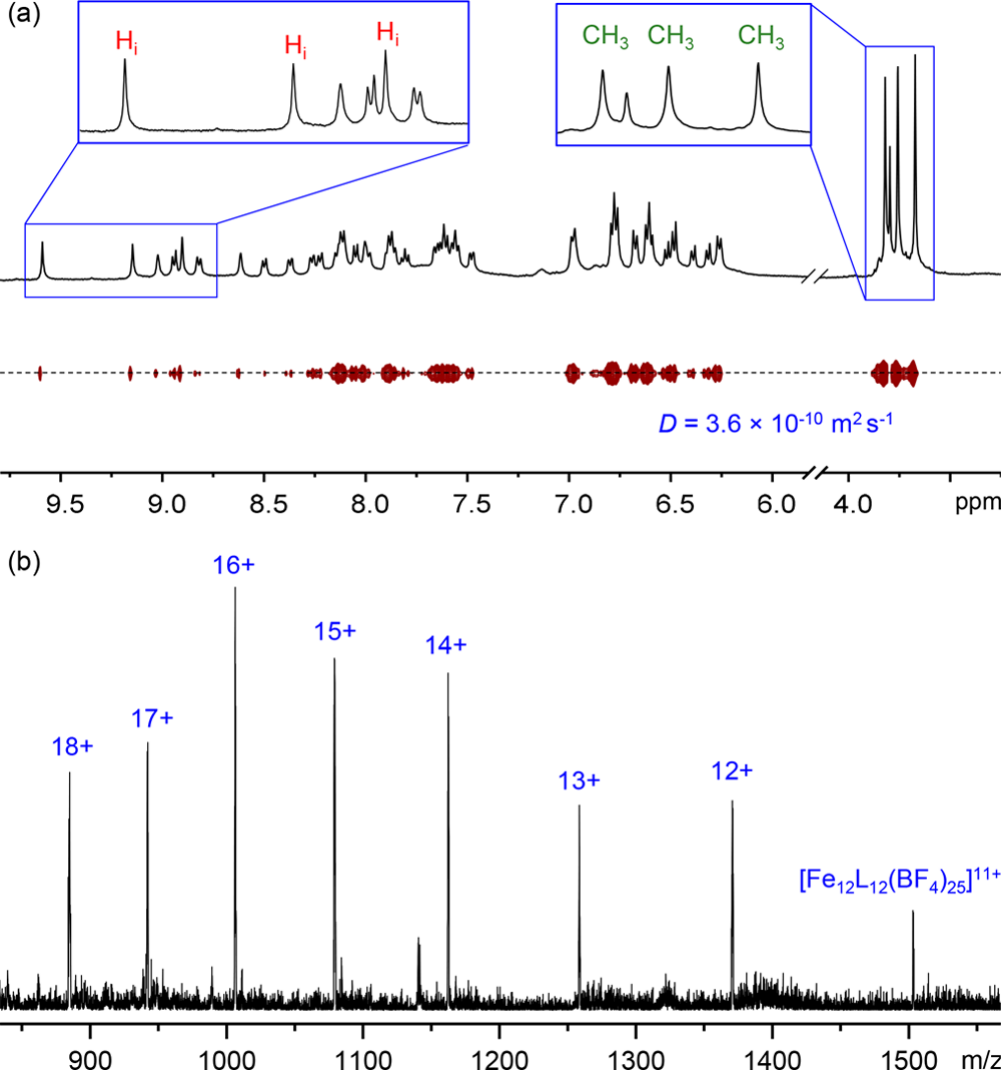
(a) ^1^H DOSY NMR spectrum (500 MHz, 298 K, CD_3_CN) of pseudoicosahedron **1**. The labeled peaks correspond to the imine and methoxy groups are highlighted in Figure 1a. (b) ESI-mass spectrum of **1**.

The ^1^H NMR spectrum of Fe^II^_12_L_12_**1** displayed a complex pattern of signals, consistent with desymmetrization of the ligand (Figures 2a and S4). Three magnetically distinct chemical environments for the protons on the initially *C*_3_-symmetric ligand were observed, with the imine and methoxy signals each exhibiting three sharp peaks with a 1:1:1 integration ratio. The ^1^H DOSY spectrum confirmed that all ^1^H signals belonged to a single species in solution (Figures 2a and S10).

These NMR and MS data were consistent with the formation of a Fe^II^_12_L_12_ pseudoicosahedral capsule with *meridional* (*mer*) coordination geometry at all iron(II) vertices (Figure 1a). The ^1^H NMR spectrum of **1** was fully assigned through 2D NMR (Figures S7–S9). This type of assembly has been observed for a smaller *C*_3_-symmetric triamine subcomponent, where a mixture of pseudoicosahedron and tetrahedron was obtained.^25^ We infer the formation of the larger capsule, rather than a Fe^II^_4_L_4_ tetrahedral cage, to result from the Coulombic repulsions between the cationic triazatriangulenium panels, which would be stronger in a tetrahedron where these panels are spatially closer together.

After many unsuccessful attempts at growing crystals of **1** suitable for X-ray diffraction, energy minimization of a pseudoicosahedral structure for **1** at the PM7 level of theory was carried out (Figure 1b and Table S2). The 12 iron(II) centers describe the vertices of an icosahedron, with the tris(bidentate) ligands capping 12 of the 20 icosahedral faces. All iron(II) centers in **1** display *mer* coordination around the metal centers, where two ligand arms extend above a triangular face of the “icosahedron” (Figure 1b, cyan and orange) and the third extends from below (Figure 1b, green). The longest Fe···Fe distance between antipodal vertices within this model of **1** is 4.1 nm, and the longest distance between the outermost methoxy groups is approximately 5.4 nm.

The PM7 model also indicates the capsule to be porous, with *C*_3_-symmetric openings, having diameters of as large as 17 Å, each surrounded by three TATA ligands (one type of opening is shown in Figure 1c). Pseudoicosahedron **1** encloses a cavity volume of 15095 Å^3^, as determined by VOIDOO calculations (Table S1).^26^ Capsule **1** thus represents the largest architecture prepared to date, to the best of our knowledge, using subcomponent self-assembly.^7i,25,27^

As the cavity of pseudoicosahedron **1** is large and positively charged, we tested the binding of a series of large anionic and neutral prospective guests. None of these prospective guests, listed in Scheme S3, gave any evidence of guest encapsulation. We infer that they are not large enough to provide a good fit for the cavity, as most have been reported to be encapsulated within smaller capsules.^28^

In contrast, when **A** (1 equiv) reacted with *p*-anisidine (3 equiv) and Fe(BF_4_)_2_ (either 0.67 or 1 equiv gave the same result) in acetonitrile at the lower **A** concentration of 2.2 mM, helicate **2** was formed instead of **1** (Scheme S4 and Figures S11–S16). An overall 7+ charge for **2** was confirmed by ESI-MS (Figure S17). The ^1^H NMR spectrum of **2** displayed signals corresponding to a *C*_2_-symmetric bis-bidentate ligand, with one pyridyl-imine arm remaining uncoordinated (Figure S11). Both the imine and methoxy ^1^H NMR signals of **2** exhibited a 2:1 integral ratio, consistent with the formation of a helicate with *D*_3_ symmetry, in which both iron(II) centers adopted the same Λ or Δ handedness.^22,29^

The formation of smaller assembly **2** at a lower concentration is expected on the basis of Le Chatelier’s principle.^30^ The electrostatic interactions between the small BF_4_^–^ anion and the small cavities of the cationic assembly may also render BF_4_^–^ a suitable template for helicate formation.

The anion binding ability of helicate **2** was confirmed by carrying out ^1^H NMR titrations. The progressive addition of tetrabutylammonium perchlorate to a solution of **2** in CD_3_CN resulted in displacement of BF_4_^–^ by ClO_4_^–^, as indicated by shifts in the helicate ^1^H signals, consistent with binding in fast exchange on the NMR chemical shift time scale (Figure S18). The addition of excess ReO_4_^–^, PF_6_^–^, or I^–^ led to similar NMR observations, but Tf_2_N^–^ did not (Figure S19). The lack of shifts in the ^1^H signals of **2** (<0.02 ppm at most) after adding excess Tf_2_N^–^ indicated negligible interactions of this anion with helicate **2** relative to BF_4_^–^.

Although numerous attempts to obtain the crystal structure of a host–guest complex of **2** were unsuccessful, we were able to obtain the crystal structure of a host–guest complex of its structural analogue **2′**, which assembled from a similar subcomponent (**B**) bearing only two pyridine-aldehyde functionalities (Figures 3a and S20–S26). Helicate **2′** bound anions in solution in similar fashion to **2** (Figure S27).

**Figure 3:**
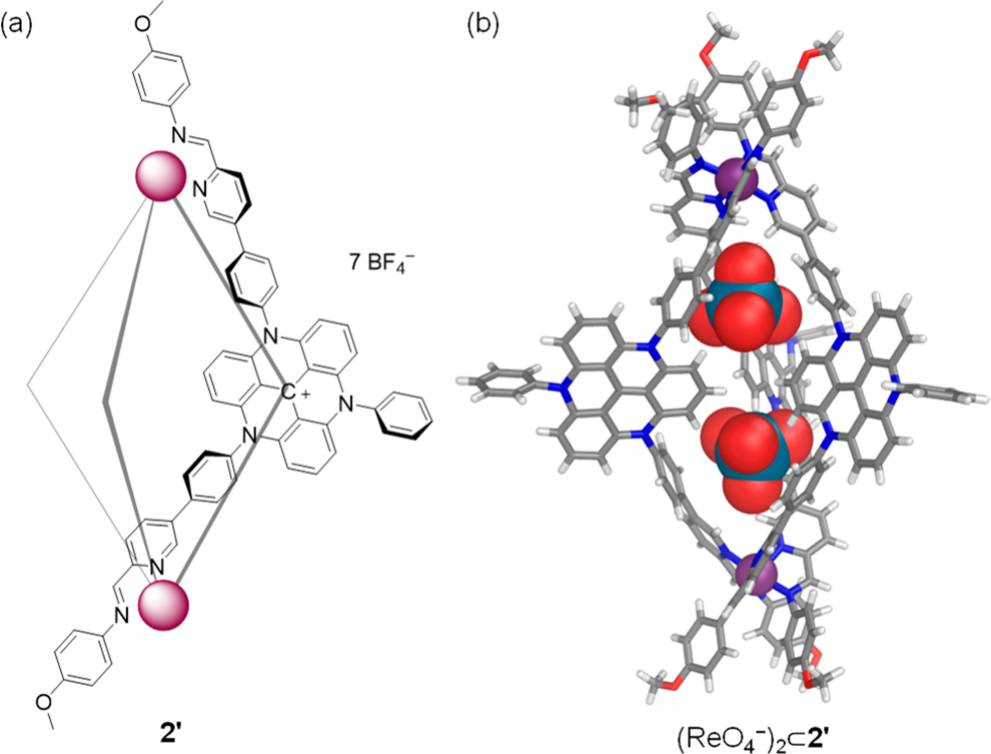
Schematic drawing of **2′** (a) and X-ray crystal structure of (ReO_4_^–^)_2_⊂**2′** (b). Disorder, noncentrally bound counterions, and solvent of crystallization are omitted for clarity.

Slow vapor diffusion of diethyl ether into an acetonitrile solution of **2′** in the presence of ReO_4_^–^ yielded crystals of (ReO_4_^–^)_2_⊂**2′** suitable for X-ray diffraction. Two ReO_4_^–^ anions were found within two distinct cavities of helicate **2′**, separated by the three converging TATA moieties (Figure 3b). Each of the two cavities is surrounded by three phenyl rings, giving cavity volumes of 76 and 77 Å^3^ (Table S1). The two iron(II) centers of **2′**, separated by 20.0 Å, have the same handedness, generating a structure with *D*_3_ symmetry, consistent with solution NMR spectra.

When larger anions were added to helicate **2** in solution, such as carba-*closo*-dodecaborate (CB_11_H_12_^–^) or dodecafluoro-*closo*-dodecaborate (B_12_F_12_^2–^), full conversion into tetrahedron **3** was observed after 12 h, resulting in a set of ligand ^1^H NMR signals consistent with a *T*-symmetric tetrahedral cage (Figures 4 and S28–S37). The Fe^II^_4_L_4_ composition of **3** was confirmed by ESI-MS (Figures S30 and S35). These results suggested that CB_11_H_12_^–^ and B_12_F_12_^2–^ could serve as templates to bring four cationic ligands together into the tetrahedral framework of **3**, overcoming interligand Columbic repulsions.^31^ A cavity volume of 371 Å^3^ was calculated based on a PM7 model of **3** (Table S4), substantially smaller than that of pseudoicosahedron **1** (15095 Å^3^) and larger than the twin cavities of **2** (55 Å^3^ each when calculated from a PM7 model of **2** in the absence of bound anions, see Table S3) (Table S1). Conversion of pseudoicosahedron **1** into tetrahedron **3** also occurred following the addition of either of the template anions CB_11_H_12_^–^ or B_12_F_12_^2–^ (Figure 4).

**Figure 4:**
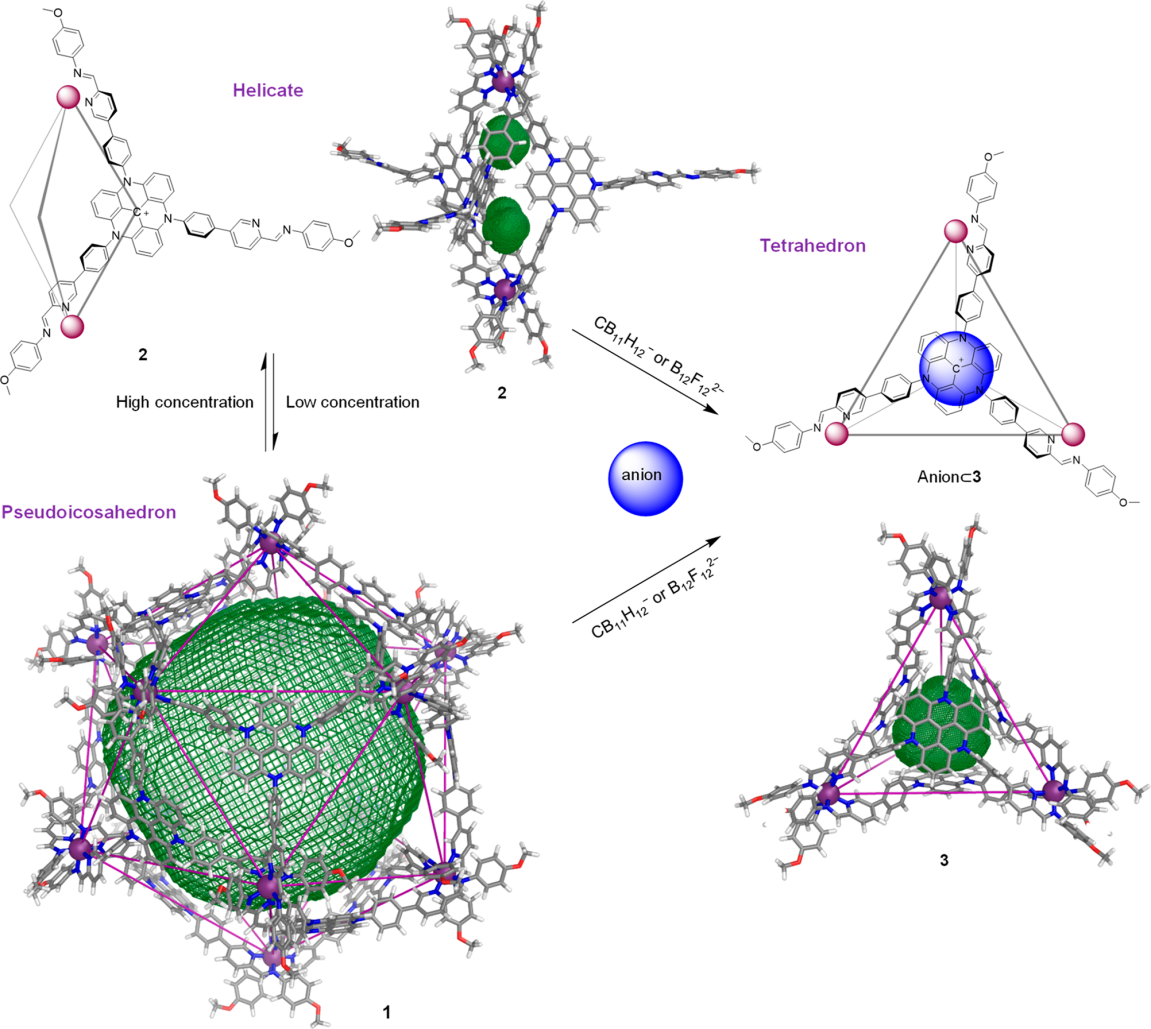
Interconversion between pseudoicosahedron **1**, helicate **2**, and tetrahedron **3**, showing PM7-optimized molecular models of each with the cavity volumes outlined in green mesh. Pseudoicosahedron **1** and helicate **2** interconverted following a change in ligand concentration. Addition of CB_11_H_12_^–^ or B_12_F_12_^2–^ to assembly **1** or **2** drove the formation of tetrahedron **3**, with the template anion bound inside the cavity.

In summary, we have demonstrated the construction of a series of distinct capsules under different reaction conditions from TATA-containing subcomponent **A**, from large pseudoicosahedron **1**, to medium-sized tetrahedron **3** and smaller helicate **2**. These structures have drastically different cavity volumes, shapes, and sizes. Pseudoicosahedron **1** encloses a cavity volume that may allow the encapsulation of guests with diameters in the range of 3–4 nm.^10^ Prior studies of smaller TATA-based metal–organic assemblies have shown binding to small biological anions in water,^24^ suggesting that water-soluble versions^32^ of pseudoicosahedron **1** may prove useful for binding larger and more complex biomolecules, such as proteins and nucleic acids.

## Supporting Information Available

Complete experimental details X-ray data for (ReO_4_^–^)_2_⊂**2′** (CCDC 2071272) (PDF)

## Accession Codes

CCDC 2071272 contains the supplementary crystallographic data for this paper. These data can be obtained free of charge via www.ccdc.cam.ac.uk/data_request/cif, or by emailing data_request@ccdc.cam.ac.uk, or by contacting The Cambridge Crystallographic Data Centre, 12 Union Road, Cambridge CB2 1EZ, UK; fax: +44 1223 336033.

## References

[ref1] aBurke B. P.; Grantham W.; Burke M. J.; Nichol G. S.; Roberts D.; Renard I.; Hargreaves R.; Cawthorne C.; Archibald S. J.; Lusby P. J. Visualizing kinetically robust Co^III^_4_L_6_ assemblies in vivo: SPECT imaging of the encapsulated [^99m^Tc]TcO_4_^–^ anion. J. Am. Chem. Soc. 2018, 140, 16877–16881. 10.1021/jacs.8b09582. 30485075

[ref2] aChan A. K.; Lam W. H.; Tanaka Y.; Wong K. M.; Yam V. W. Multiaddressable molecular rectangles with reversible host-guest interactions: modulation of pH-controlled guest release and capture. Proc. Natl. Acad. Sci. U.S.A. 2015, 112, 690–695. 10.1073/pnas.1423709112. 25568083 PMC4311808

[ref3] aYou L.; Berman J. S.; Anslyn E. V. Dynamic multi-component covalent assembly for the reversible binding of secondary alcohols and chirality sensing. Nat. Chem. 2011, 3, 943–948. 10.1038/nchem.1198. 22109274 PMC3226768

[ref4] aZhang D.; Ronson T. K.; Zou Y.-Q.; Nitschke J. R. Metal–organic cages for molecular separations. Nat. Rev. Chem. 2021, 5, 168–182. 10.1038/s41570-020-00246-1. 37117530

[ref5] Mal P.; Breiner B.; Rissanen K.; Nitschke J. R. White phosphorus is air-stable within a self-assembled tetrahedral capsule. Science 2009, 324, 1697–1699. 10.1126/science.1175313. 19556504

[ref6] aYoshizawa M.; Tamura M.; Fujita M. Diels-alder in aqueous molecular hosts: Unusual regioselectivity and efficient catalysis. Science 2006, 312, 251–254. 10.1126/science.1124985. 16614218

[ref7] aZhang D.; Ronson T. K.; Nitschke J. R. Functional capsules via subcomponent self-assembly. Acc. Chem. Res. 2018, 51, 2423–2436. 10.1021/acs.accounts.8b00303. 30207688

[ref8] aWang H.; Liu C. H.; Wang K.; Wang M.; Yu H.; Kandapal S.; Brzozowski R.; Xu B.; Wang M.; Lu S.; Hao X. Q.; Eswara P.; Nieh M. P.; Cai J.; Li X. Assembling pentatopic terpyridine ligands with three types of coordination moieties into a giant supramolecular hexagonal prism: synthesis, self-assembly, characterization, and antimicrobial study. J. Am. Chem. Soc. 2019, 141, 16108–16116. 10.1021/jacs.9b08484. 31509694 PMC6849473

[ref9] aDouglas T.; Young M. Host–guest encapsulation of materials by assembled virus protein cages. Nature 1998, 393, 152–155. 10.1038/30211.

[ref10] aFujita D. Challenges to large molecular encapsulation. Pur. Appl. Chem. 2014, 86, 3–11. 10.1515/pac-2014-5009.

[ref11] aSaibil H. R.; Fenton W. A.; Clare D. K.; Horwich A. L. Structure and allostery of the chaperonin GroEL. J. Mol. Biol. 2013, 425, 1476–1487. 10.1016/j.jmb.2012.11.028. 23183375

[ref12] Wang W.; Wang Y. X.; Yang H. B. Supramolecular transformations within discrete coordination-driven supramolecular architectures. Chem. Soc. Rev. 2016, 45, 2656–2693. 10.1039/C5CS00301F. 27009833

[ref13] aKishi N.; Akita M.; Kamiya M.; Hayashi S.; Hsu H. F.; Yoshizawa M. Facile catch and release of fullerenes using a photoresponsive molecular tube. J. Am. Chem. Soc. 2013, 135, 12976–12979. 10.1021/ja406893y. 23957216

[ref14] Chen J.; Wezenberg S. J.; Feringa B. L. Intramolecular transport of small-molecule cargo in a nanoscale device operated by light. Chem. Commun. 2016, 52, 6765–6768. 10.1039/C6CC02382G. 27068214

[ref15] Shanmugaraju S.; Umadevi D.; Savyasachi A. J.; Byrne K.; Ruether M.; Schmitt W.; Watson G. W.; Gunnlaugsson T. Reversible adsorption and storage of secondary explosives from water using a Tröger’s base-functionalised polymer. J. Mater. Chem. A 2017, 5, 25014–25024. 10.1039/C7TA07292A.

[ref16] aCullen W.; Turega S.; Hunter C. A.; Ward M. D. pH-dependent binding of guests in the cavity of a polyhedral coordination cage: reversible uptake and release of drug molecules. Chem. Sci. 2015, 6, 625–631. 10.1039/C4SC02090A. 28936311 PMC5588781

[ref17] aChen S.; Chen L. J.; Yang H. B.; Tian H.; Zhu W. Light-triggered reversible supramolecular transformations of multi-bisthienylethene hexagons. J. Am. Chem. Soc. 2012, 134, 13596–13599. 10.1021/ja306748k. 22881042

[ref18] aKishimoto M.; Kondo K.; Akita M.; Yoshizawa M. A pH-responsive molecular capsule with an acridine shell: catch and release of large hydrophobic compounds. Chem. Commun. 2017, 53, 1425–1428. 10.1039/C6CC09094J. 28079195

[ref19] aWang S.; Yao C.; Ni M.; Xu Z.; Cheng M.; Hu X.-Y.; Shen Y.-Z.; Lin C.; Wang L.; Jia D. Thermo- and oxidation-responsive supramolecular vesicles constructed from self-assembled pillar[6]arene-ferrocene based amphiphilic supramolecular diblock copolymers. Polym. Chem. 2017, 8, 682–688. 10.1039/C6PY01961G.

[ref20] aHeo J.; Jeon Y. M.; Mirkin C. A. Reversible interconversion of homochiral triangular macrocycles and helical coordination polymers. J. Am. Chem. Soc. 2007, 129, 7712–7713. 10.1021/ja0716812. 17539639

[ref21] aFrischmann P. D.; Kunz V.; Stepanenko V.; Wurthner F. Subcomponent self-assembly of a 4 nm M_4_L_6_ tetrahedron with Zn(II) vertices and perylene bisimide dye edges. Chem.—Eur. J. 2015, 21, 2766–2769. 10.1002/chem.201405866. 25582932

[ref22] Zhang D.; Ronson T. K.; Xu L.; Nitschke J. R. Transformation network culminating in a heteroleptic Cd_6_L_6_L’_2_ twisted trigonal prism. J. Am. Chem. Soc. 2020, 142, 9152–9157. 10.1021/jacs.0c03798. 32357009 PMC7243256

[ref23] aLi B.; Zhang W.; Lu S.; Zheng B.; Zhang D.; Li A.; Li X.; Yang X. J.; Wu B. Multiple transformations among anion-based A_2n_L_3n_ assemblies: bicapped trigonal antiprism A_8_L_12_, tetrahedron A_4_L_6_, and triple helicate A_2_L_3_ (A = Anion). J. Am. Chem. Soc. 2020, 142, 21160–21168. 10.1021/jacs.0c10346. 33272016

[ref24] Plajer A. J.; Percastegui E. G.; Santella M.; Rizzuto F. J.; Gan Q.; Laursen B. W.; Nitschke J. R. Fluorometric recognition of nucleotides within a water-soluble tetrahedral capsule. Angew. Chem., Int. Ed. 2019, 58, 4200–4204. 10.1002/anie.201814149. 30666756

[ref25] Bilbeisi R. A.; Ronson T. K.; Nitschke J. R. A self-assembled [Fe^II^_12_L_12_] capsule with an icosahedral framework. Angew. Chem., Int. Ed. 2013, 52, 9027–9030. 10.1002/anie.201302976. 23857765

[ref26] Kleywegt G. J.; Jones T. A. Detection, delineation, measurement and display of cavities in macromolecular structures. Acta Crystallogr. 1994, D50, 178–185. 10.1107/S0907444993011333. 15299456

[ref27] aRizzuto F. J.; Nitschke J. R. Stereochemical plasticity modulates cooperative binding in a Co^II^_12_L_6_ cuboctahedron. Nat. Chem. 2017, 9, 903–908. 10.1038/nchem.2758. 28837174

[ref28] aZhang D.; Ronson T. K.; Greenfield J. L.; Brotin T.; Berthault P.; Leonce E.; Zhu J. L.; Xu L.; Nitschke J. R. Enantiopure [Cs^+^/Xe⊂cryptophane]⊂Fe^II^_4_L_4_ hierarchical superstructures. J. Am. Chem. Soc. 2019, 141, 8339–8345. 10.1021/jacs.9b02866. 31034215

[ref29] Bilbeisi R. A.; Clegg J. K.; Elgrishi N.; de Hatten X.; Devillard M.; Breiner B.; Mal P.; Nitschke J. R. Subcomponent self-assembly and guest-binding properties of face-capped Fe_4_L_4_^8+^ capsules. J. Am. Chem. Soc. 2012, 134, 5110–5119. 10.1021/ja2092272. 22043943

[ref30] Bai X.; Jia C.; Zhao Y.; Yang D.; Wang S. C.; Li A.; Chan Y. T.; Wang Y. Y.; Yang X. J.; Wu B. Peripheral templation-modulated interconversion between an A_4_L_6_ tetrahedral anion cage and A_2_L_3_ triple helicate with guest capture/release. Angew. Chem., Int. Ed. 2018, 57, 1851–1855. 10.1002/anie.201712080. 29251815

[ref31] Zhang D.; Ronson T. K.; Mosquera J.; Martinez A.; Guy L.; Nitschke J. R. Anion binding in water drives structural adaptation in an azaphosphatrane-functionalized Fe^II^_4_L_4_ tetrahedron. J. Am. Chem. Soc. 2017, 139, 6574–6577. 10.1021/jacs.7b02950. 28463507

[ref32] Percastegui E. G.; Ronson T. K.; Nitschke J. R. Design and applications of water-soluble coordination cages. Chem. Rev. 2020, 120, 13480–13544. 10.1021/acs.chemrev.0c00672. 33238092 PMC7760102

