# Supplementary material for: Templation and Concentration Drive Conversion Between a FeII12L12 Pseudoicosahedron, a FeII4L4 Tetrahedron, and a FeII2L3 Helicate
Source: J Am Chem Soc. 2022 Jan 11;144(3):1106–12. doi: 10.1021/jacs.1c11536 (PMC9097479; doi:10.1021/jacs.1c11536)
Supplement: Supplementary file 1 — ja1c11536_si_001.pdf [file ja1c11536_si_001.pdf]

Supporting Information for

Templation and Concentration Drive Conversion Between a $Fe^{II}_{12}L_{12}$ Pseudo-icosahedron, a $Fe^{II}_4L_4$ Tetrahedron, and a $Fe^{II}_2L_3$ Helicate

Dawei Zhang,[†,‡] Quan Gan,[‡,#] Alex J. Plajer,[‡,⊥] Roy Lavendomme,[§] Tanya K. Ronson,[‡] Zifei Lu,[‡] Jesper D. Jensen,[∥] Bo W. Laursen,[∥] and Jonathan R. Nitschke[‡,*]

[†]Shanghai Key Laboratory of Green Chemistry and Chemical Processes, School of Chemistry and Molecular Engineering, East China Normal University, Shanghai 200062, People's Republic of China.
[‡]Department of Chemistry, University of Cambridge, Lensfield Road, Cambridge, CB2 1EW, United Kingdom.
[#]Hubei Key Laboratory of Bioinorganic Chemistry & Materia Medica, School of Chemistry and Chemical Engineering, Huazhong University of Science and Technology, Wuhan 430074, People's Republic of China.
[⊥]Oxford Chemistry, Chemical Research Laboratory, 12 Mansfield Road, Oxford, OX1 3TA (UK).
[§]COMOC − Center for Ordered Materials, Organometallics and Catalysis, Department of Chemistry, Ghent University, Krijgslaan 281-S3, 9000 Ghent, Belgium.
[∥]Department of Chemistry & Nano-Science Center, University of Copenhagen, Universitetsparken 5, 2100, Copenhagen, Denmark.

Contents

1. Materials and methods 2
2. Synthesis of subcomponent **B** 3
3. Self-assembly and characterization of pseudo-icosahedron **1** 5
4. Self-assembly and characterization of helicate **2** 12
5. Self-assembly and characterization of helicate **2'** 18
6. Self-assembly and characterization of tetrahedron **3** 23
7. X-ray crystallography 28
8. Volume calculations 30
9. Molecular modelling 31
10. References 47

1. Materials and methods

Unless otherwise specified, reagents and solvents were purchased from commercial suppliers and used without further purification.

Centrifugation of cage samples was carried out using a Grant-Bio LMC-3000 low speed benchtop centrifuge.

NMR spectra were recorded using a Bruker Avance 500 MHz (^{1}H, ^{19}F and 2D experiments), a Bruker DCH 500 MHz dual cryoprobe (high-resolution ^{13}C), and a Bruker Avance 500 MHz TCI-ATM cryoprobe (^{1}H NMR) in deuterated solvents. Chemical shifts for ^{1}H, ^{13}C, and ^{19}F NMR are reported in ppm on the δ scale; ^{1}H and ^{13}C were referenced to the residual solvent peak. ^{19}F was referenced to the $CFCl_3$ external standard in the NMR spectrometer. Coupling constants (J) are reported in Hz. The following abbreviations are used to describe signal multiplicity for ^{1}H, ^{13}C and ^{19}F NMR spectra: s: singlet, d: doublet, t: triplet, dd: doublet of doublets; dt: doublet of triplets; m: multiplet.

DOSY experiments were performed on a Bruker DPX S5 500 MHz BB ATM spectrometer. Maximum gradient strength was 6.57 G/cmA. The standard Bruker pulse program, ledbpgp2s, employing a stimulated echo and longitudinal eddy-current delay (LED) using bipolar gradient pulses for diffusion was utilized. Rectangular gradients were used with a total duration of 1.5 ms. Gradient recovery delays were 1200 µs. Individual rows of the quasi-2D diffusion databases were phased and baseline corrected.

A microwave reactor from Discover SP-D 80-CEM Corporation was used for the preparation of metal-organic assemblies in particular cases. Low resolution electrospray ionization mass spectrometry was undertaken on a Micromass Quattro LC mass spectrometer (cone voltage 10-30 eV; desolvation temp. 40 °C; ionization temp. 40 °C) infused from a Harvard syringe pump at a rate of 10 µL/min.

2. Synthesis of subcomponent B

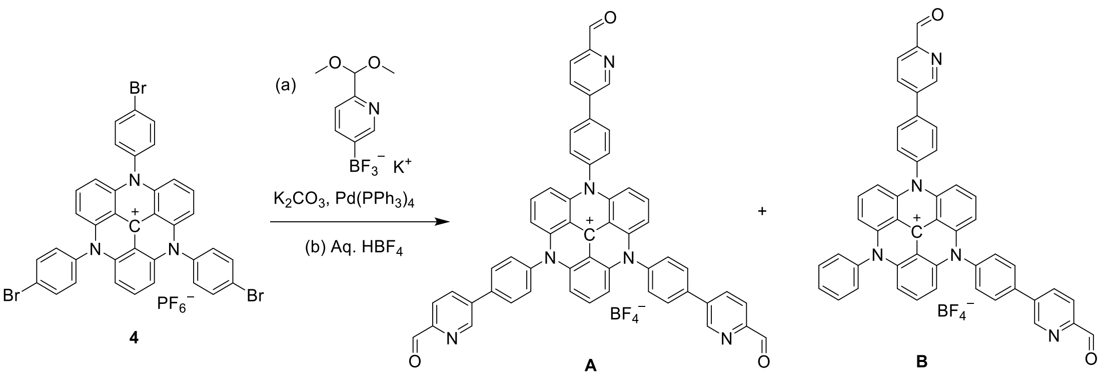

Scheme S1. Synthesis of subcomponent **B**.

Compound **4** and subcomponent **A** were synthesized according to reported procedures.[1,2]

Subcomponent **B** was obtained as a secondary product during the synthesis of subcomponent **A**. A 100 mL Schlenk flask was charged with compound **4** (357 mg, 0.400 mmol), potassium carbonate (680 mg, 4.80 mmol, 12 equiv.) and potassium(2-(dimethoxymethyl)-5-(trifluoroboranyl)pyridine) (342 mg, 1.32 mmol, 3.3 equiv.). A 1:1 H_2O:DMF mixture (40 mL) was added and nitrogen was bubbled through the resulting solution for 1 hour. Tetrakis(triphenylphosphine)-palladium(0) (69 mg, 0.060 mmol, 15 mol%) was added under a stream of nitrogen and the reaction mixture was stirred and heated at 100 °C under a nitrogen atmosphere for 3 days; the colour of the crude mixture changed from red to orange during this time. The reaction mixture was poured into 100 mL H_2O and extracted 4 times with 50 mL of DCM. The combined organic phases were dried over anhydrous $MgSO_4$ and the solvent was removed *in vacuo*. The crude coupling products were purified by column chromatography (SiO_2, eluent 2:100 MeOH:DCM) and the precursors of subcomponents **A** and **B** were successfully separated at this stage. The precursor of subcomponent **B** was dissolved in 10 mL of a 1:1:2 HBF_4:H_2O:THF mixture and stirred for 1 week at room temperature. Afterwards, the reaction mixture was slowly poured into 50 mL of an aqueous Na_2CO_3 solution and the resulting suspension was extracted 4 times with 15 mL of DCM. The combined organic fractions were dried over anhydrous $MgSO_4$ and the solvent was removed *in vacuo* to yield subcomponent **B** (70.7 mg, 0.088 mmol, 22%) as a red powder.

^{1}H NMR (500 MHz, 298 K, CD_3CN): δ (ppm) 10.11 (d, J = 0.7 Hz, 2H), 9.23 (dd, J = 0.7 Hz, 2.2 Hz, 2H), 8.37-8.35 (m, 2H), 8.24-8.21 (m, 4H), 8.10 (dd, J = 0.75 Hz, 8.1 Hz, 2H), 7.89-7.86 (m, 2H), 7.81-7.78 (t, J = 7.5 Hz, 1H), 7.72-7.67 (m, 7H), 7.56-7.54 (m, 2H), 6.52-6.49 (m, 4H), 6.44 (d, J = 8.1 Hz, 2H). **^{13}C NMR** (125.8 MHz, 298 K, CD_3CN): δ (ppm) 193.2,

152.3,148.9, 142.4, 142.1, 142.0(1), 141.9(8), 138.9(1), 138.8(5), 138.4(9), 137.9, 137.4(1), 137.3(8), 135.9, 132.2, 131.3, 130.6, 129.6, 128.5, 121.7, 110.3, 110.2, 107.2, 107.1, 107.0. **^{19}F NMR** (470.4 MHz, 298 K, CD_3CN): δ -151.84, -151.89 ppm. **ESI-MS**: *m/z* found: 720.32, $[M]^+$; $C_{49}H_{30}N_5O_2$ requires: 720.24.

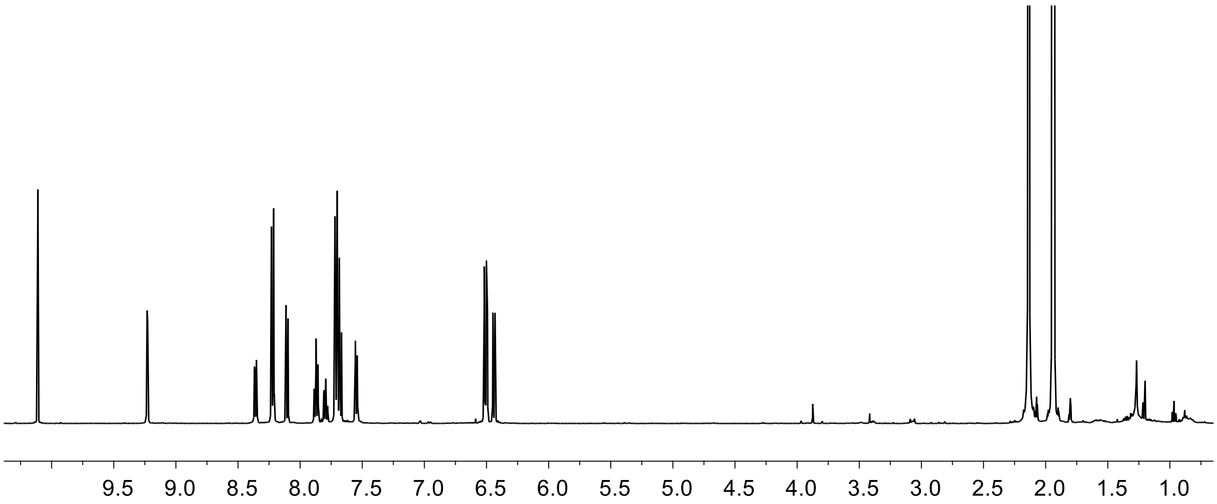

Figure S1. ^{1}H NMR spectrum (500 MHz, 298 K, CD_3CN) of subcomponent **B**.

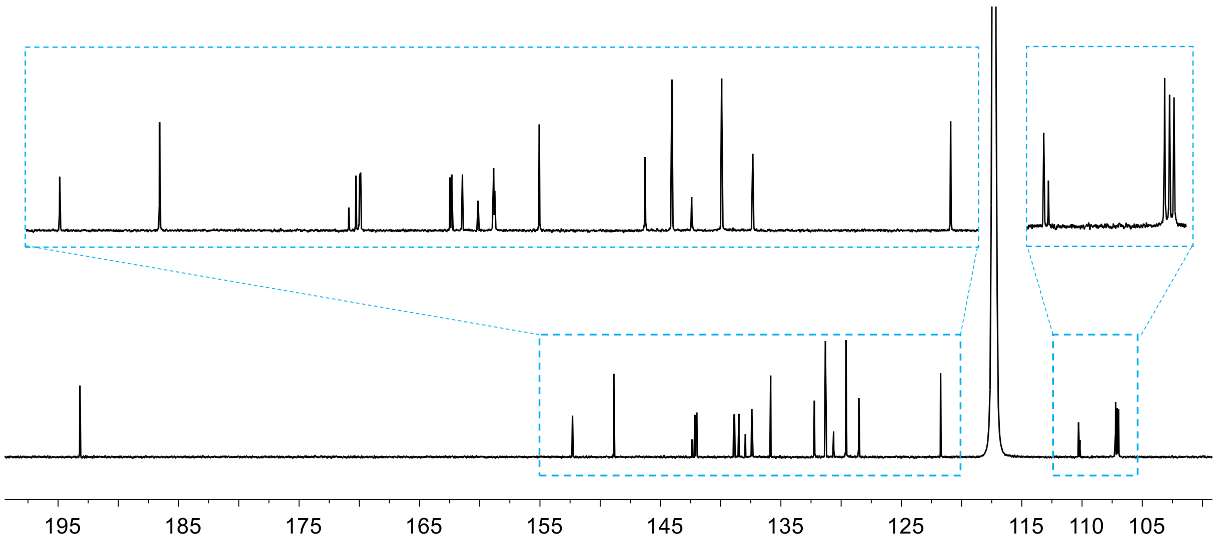

Figure S2. ^{13}C NMR spectrum (125.8 MHz, 298 K, CD_3CN) of subcomponent **B**.

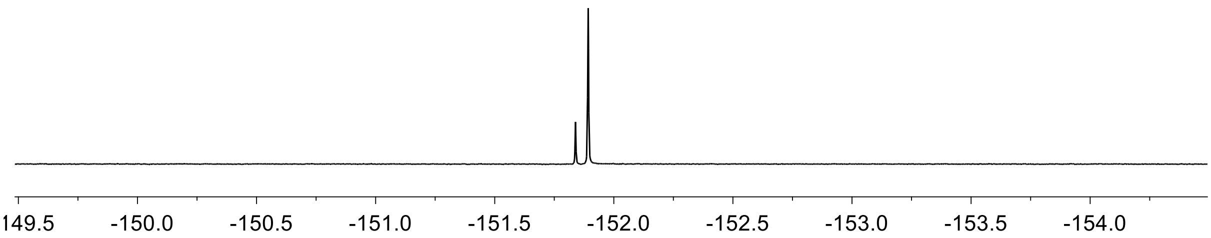

Figure S3. ^{19}F NMR spectrum (CD_3CN, 298 K, 470 MHz) of subcomponent **B**.

3. Self-assembly and characterization of pseudo-icosahedron 1

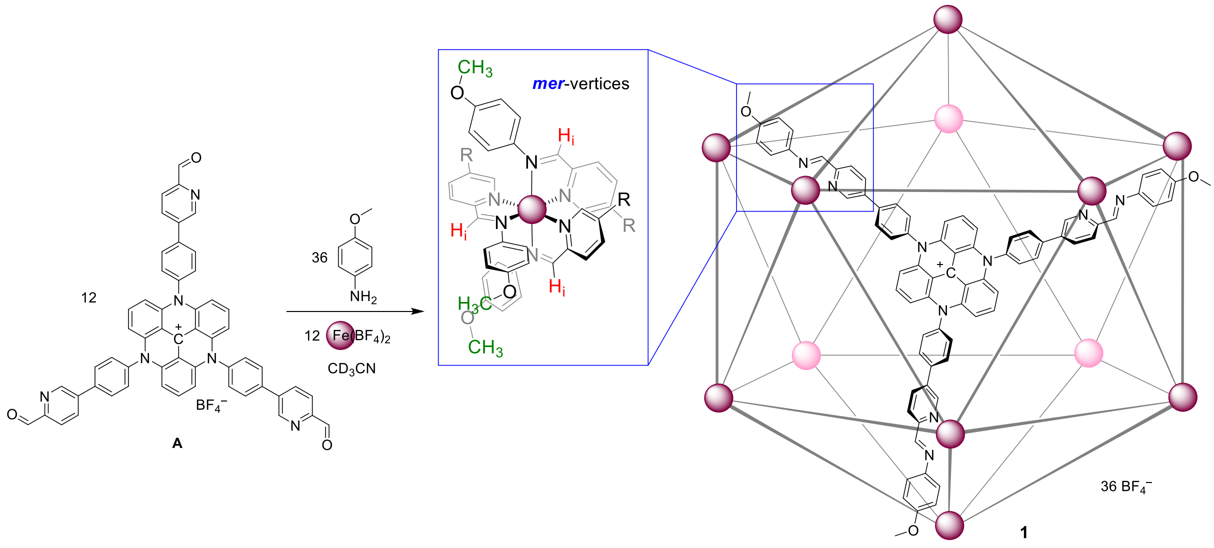

Scheme S2. Subcomponent self-assembly of pseudo-icosahedron **1**.

Subcomponent **A** (2.0 mg, 2.2 µmol, 1.0 equiv), *p*-anisidine (0.81 mg, 6.6 µmol, 3.0 equiv), and $Fe(BF_4)_2 \cdot 6H_2O$ (0.74 mg, 2.2 µmol, 1.0 equiv) were combined in CD_3CN (0.5 mL) and stirred at 70 °C for two days. The red reaction mixture produced pseudo-icosahedron **1** in a clean form in solution without further purification. **^{1}H NMR** (500 MHz, 298 K, CD_3CN): δ (ppm) 9.58 (s, 12H), 9.14 (s, 12H), 9.02 (s, 12H), 8.93 (d, J = 8.4 Hz, 12H), 8.90 (s, 12H), 8.82 (d, J = 8.0 Hz, 12H), 8.61 (s, 12H), 8.49 (d, J = 7.3 Hz, 12H), 8.37 (d, J = 7.9 Hz, 12H), 8.26 (d, J = 9.0 Hz, 12H), 8.22 (d, J = 8.5 Hz, 12H), 8.14-8.10 (m, 36H), 8.04 (d, J = 8.1 Hz, 12H), 7.98 (m, 24H), 7.89-7.87 (m, 48H), 7.80 (t, J = 8.2 Hz, 12H), 7.66-7.54 (m, 60H), 7.47 (d, J = 8.2 Hz, 12H), 6.97 (d, J = 7.7 Hz, 24H), 6.79-6.76 (m, 48H), 6.67 (d, J = 8.6 Hz, 24H), 6.61-6.58 (m, 36H), 6.51 (d, J = 8.5 Hz, 12H), 6.48 (d, J = 8.4 Hz, 12H), 6.38 (d, J = 8.4 Hz, 12H), 6.31 (d, J = 8.4 Hz, 12H), 6.25 (d, J = 8.4 Hz, 24H), 3.81 (s, 36H), 3.75 (s, 36H), 3.67 (s, 36H). **^{13}C NMR** (125.8 MHz, 298 K, CD_3CN): δ (ppm) 174.6, 171.5, 170.5, 161.3, 161.0, 160.9, 158.9, 158.8, 158.7, 158.4, 155.4, 154.7, 154.5, 145.7, 144.8, 143.4, 143.2, 143.0, 142.9, 142.8, 142.7, 142.5, 141.3, 141.1, 141.0, 140.9, 140.5, 140.2, 140.1, 139.2, 138.4(3), 138.3(7), 138.2, 138.0, 137.7, 137.6, 137.1, 137.0, 136.1, 133.0, 132.1, 132.0, 131.9, 131.1, 130.9, 130.7, 130.6, 130.2, 129.2, 125.1, 124.4, 122.5, 115.8, 115.6, 115.3, 115.2, 111.4, 111.2, 108.1, 107.9, 107.7, 56.5, 56.4, 56.2. **^{19}F NMR** (470 MHz, 298 K, CD_3CN): δ (ppm) -151.4. **ESI-MS**: m/z 884.6 $[M+18BF_4^-]^{18+}$, 941.7 $[M+19BF_4^-]^{17+}$, 1006.0 $[M+20BF_4^-]^{16+}$, 1278.9 $[M+21BF_4^-]^{15+}$, 1162.2 $[M+22BF_4^-]^{14+}$, 1258.3 $[M+23BF_4^-]^{13+}$, 1370.4 $[M+24BF_4^-]^{12+}$, 1502.9 $[M+25BF_4^-]^{11+}$.

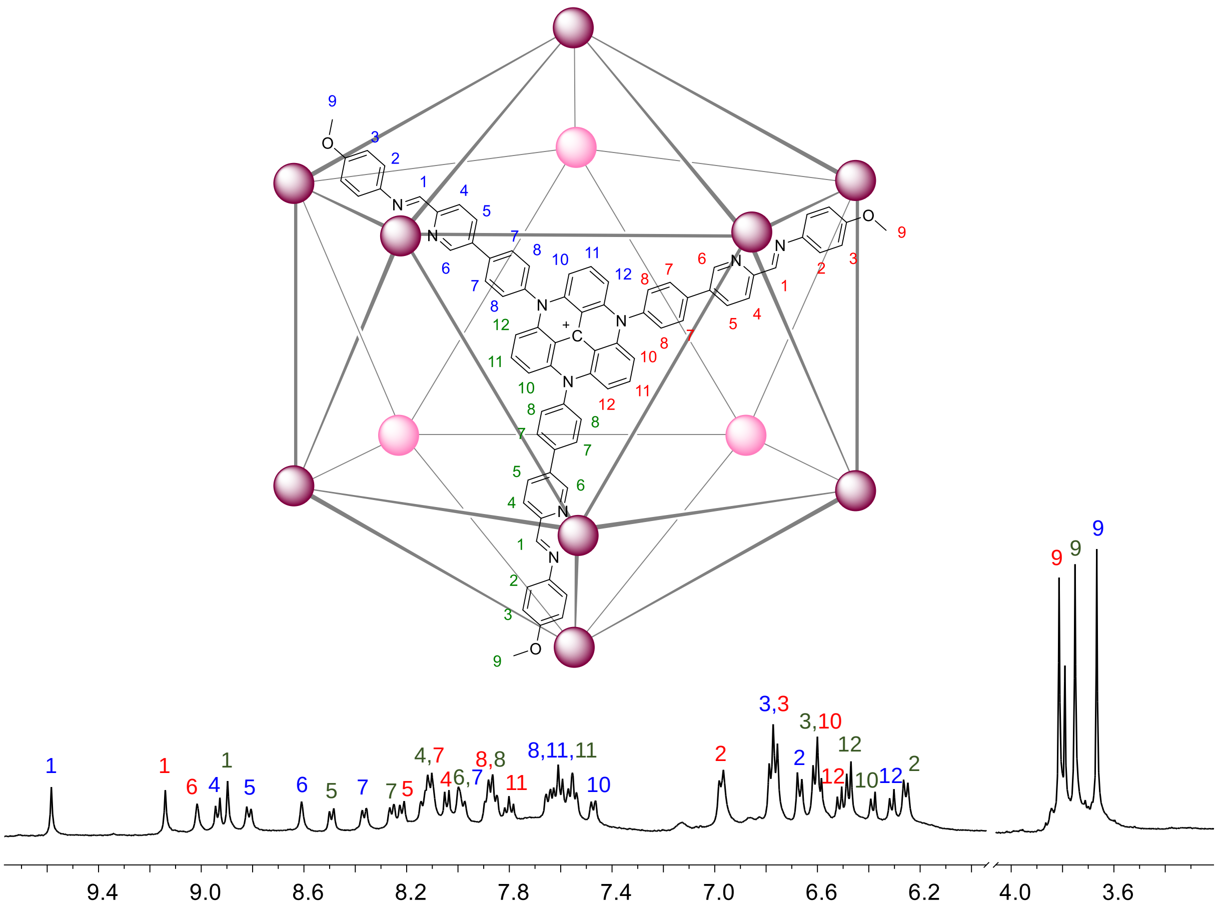

Figure S4. ^{1}H NMR spectrum (500 MHz, 298 K, CD_3CN) of pseudo-icosahedron **1**.

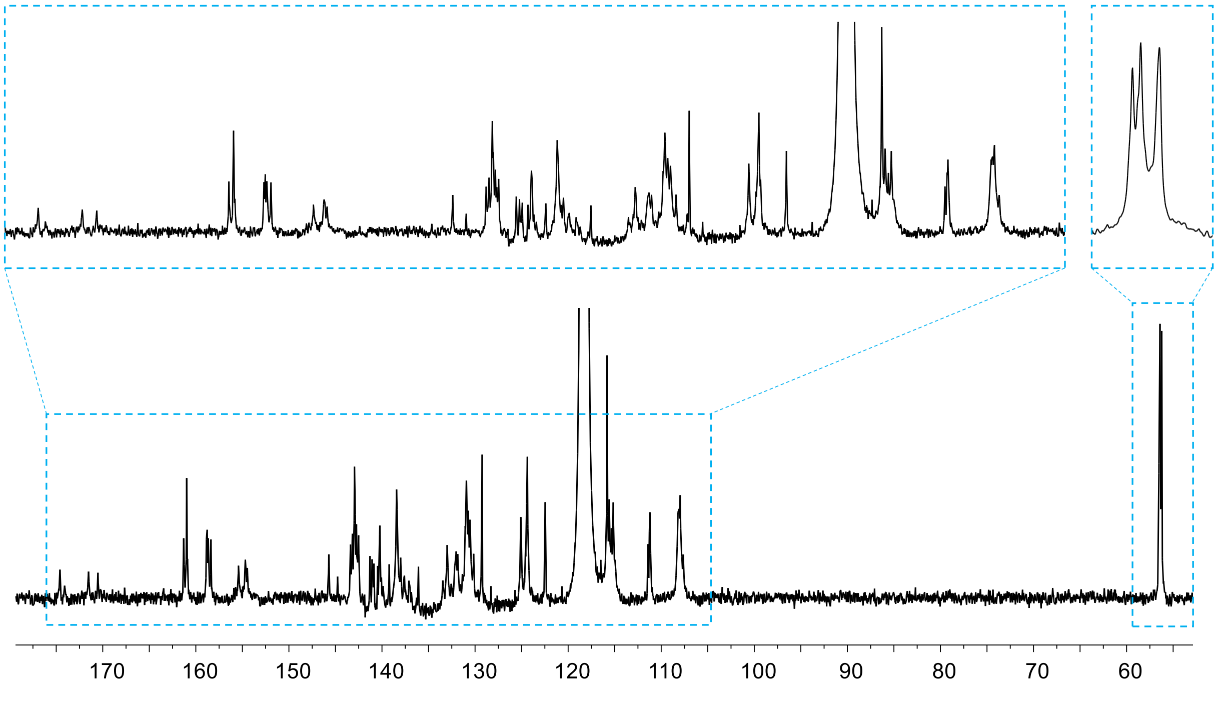

Figure S5. ^{13}C NMR spectrum (125.8 MHz, 298 K, CD_3CN) of pseudo-icosahedron **1**.

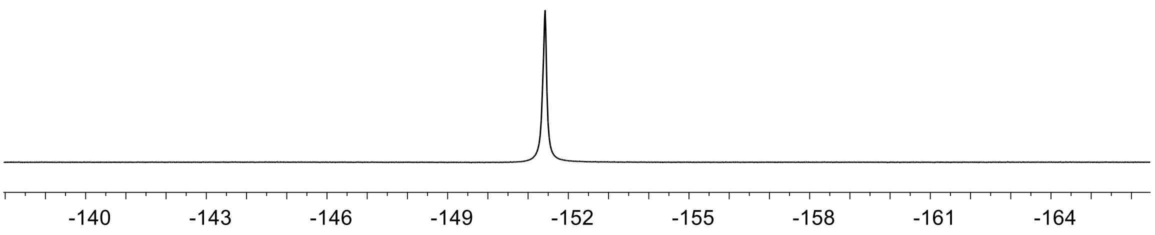

Figure S6. ^{19}F NMR spectrum (470 MHz, 298 K, CD_3CN) of pseudo-icosahedron **1**.

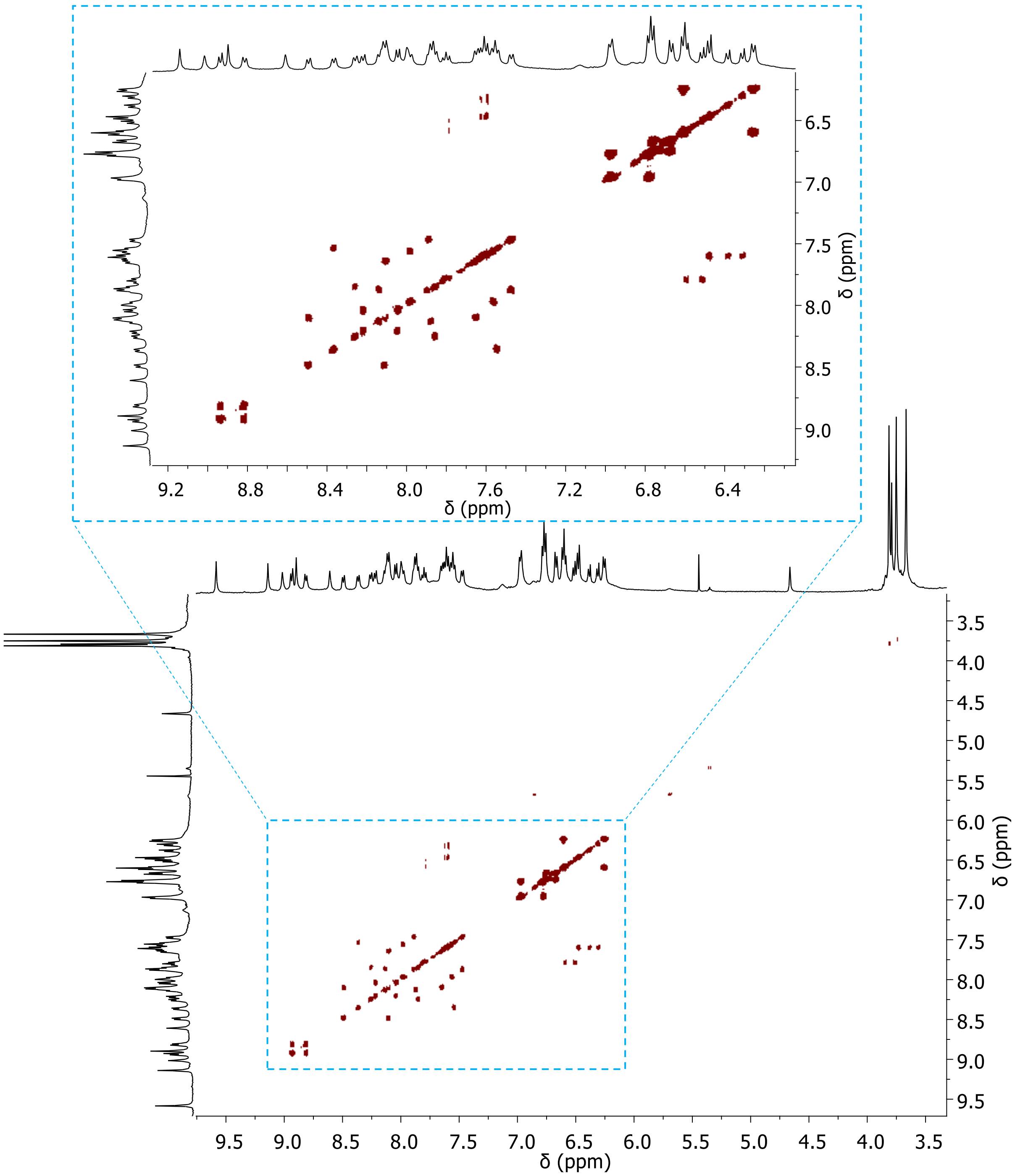

Figure S7. ^{1}H-^{1}H COSY spectrum (500 MHz, 298 K, CD_3CN) of pseudo-icosahedron **1**.

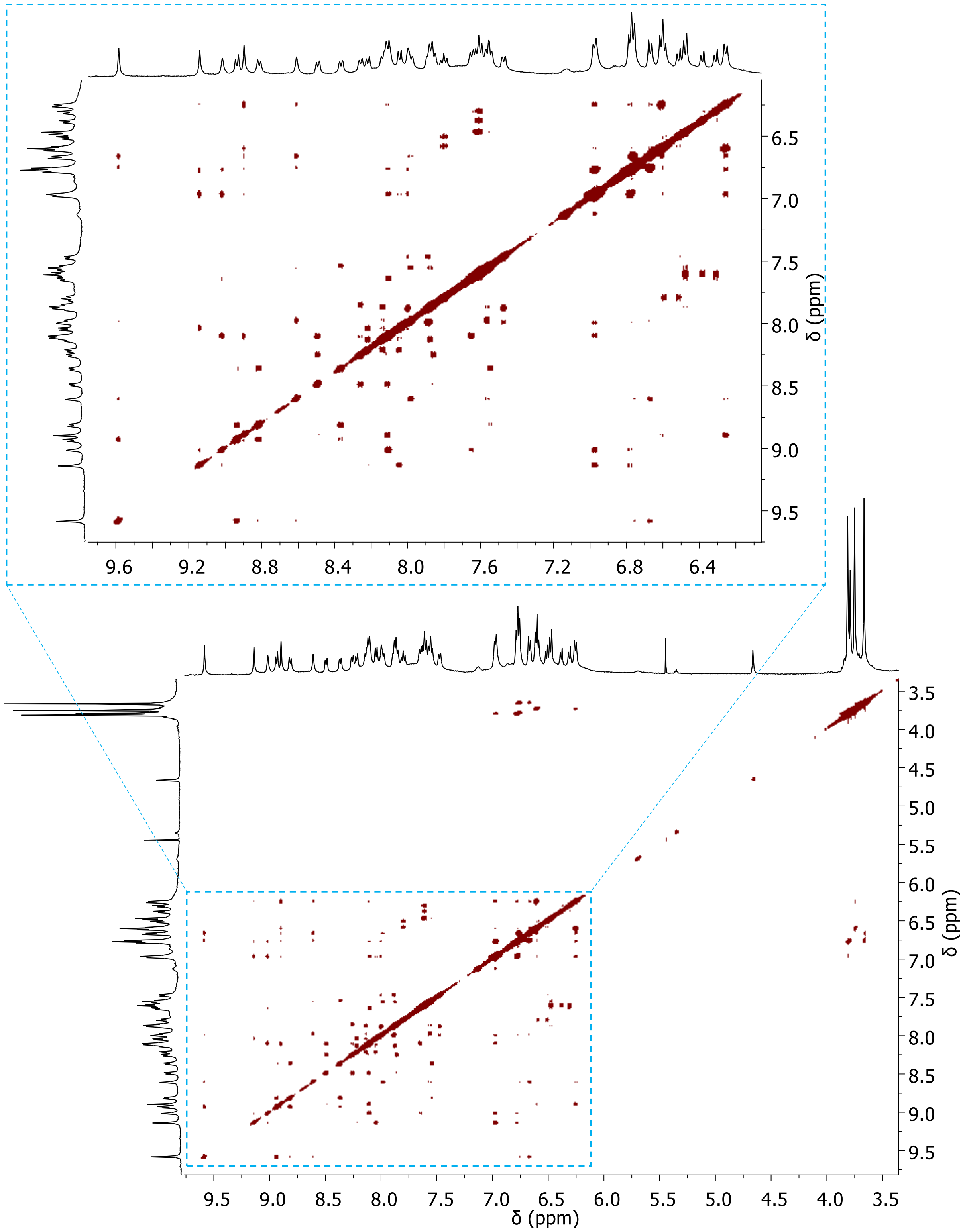

Figure S8. ^{1}H-^{1}H NOESY spectrum (500 MHz, 298 K, CD_3CN) of pseudo-icosahedron **1**.

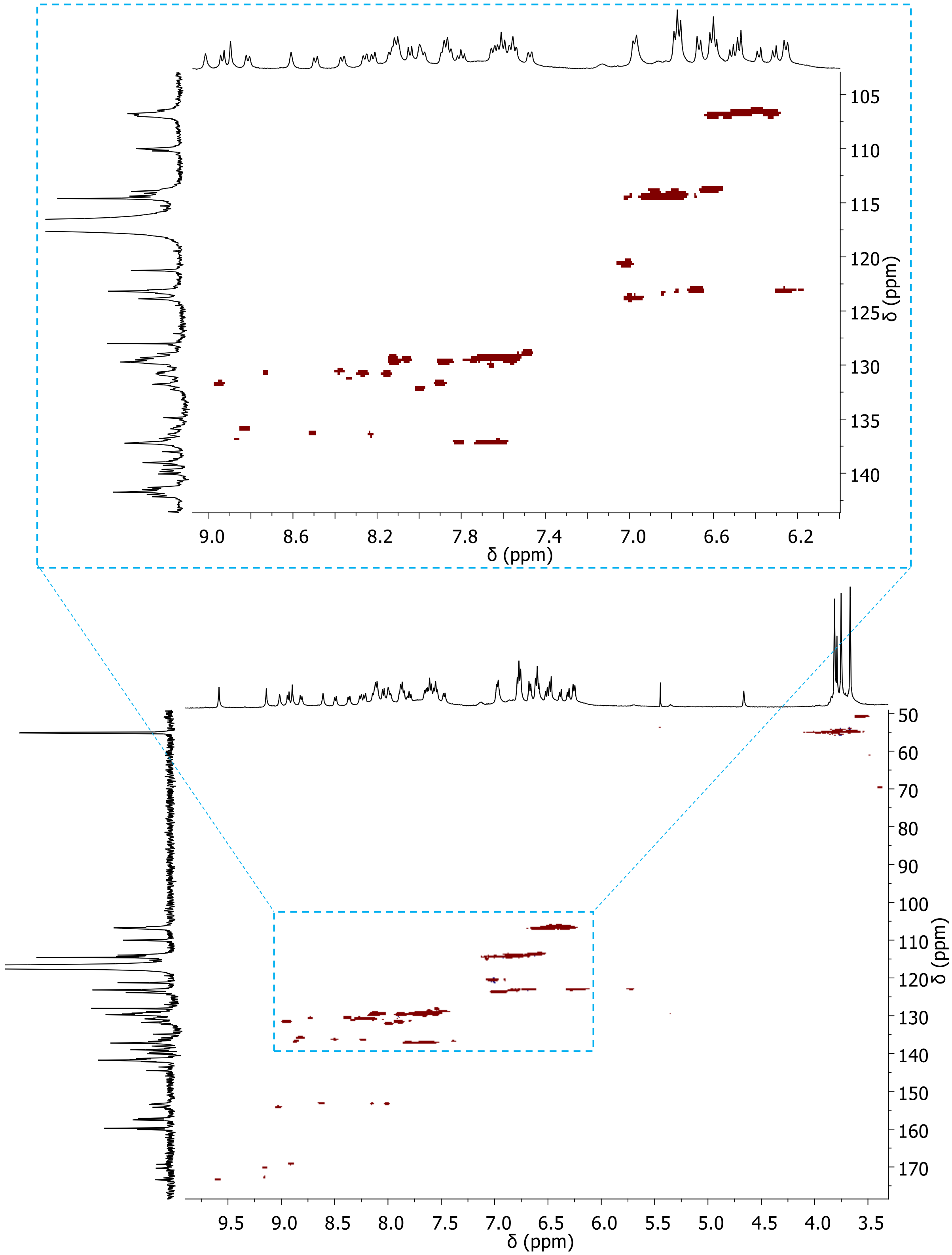

Figure S9. ^{1}H-^{13}C HSQC spectrum (500 MHz, 298 K, CD_3CN) of pseudo-icosahedron **1**.

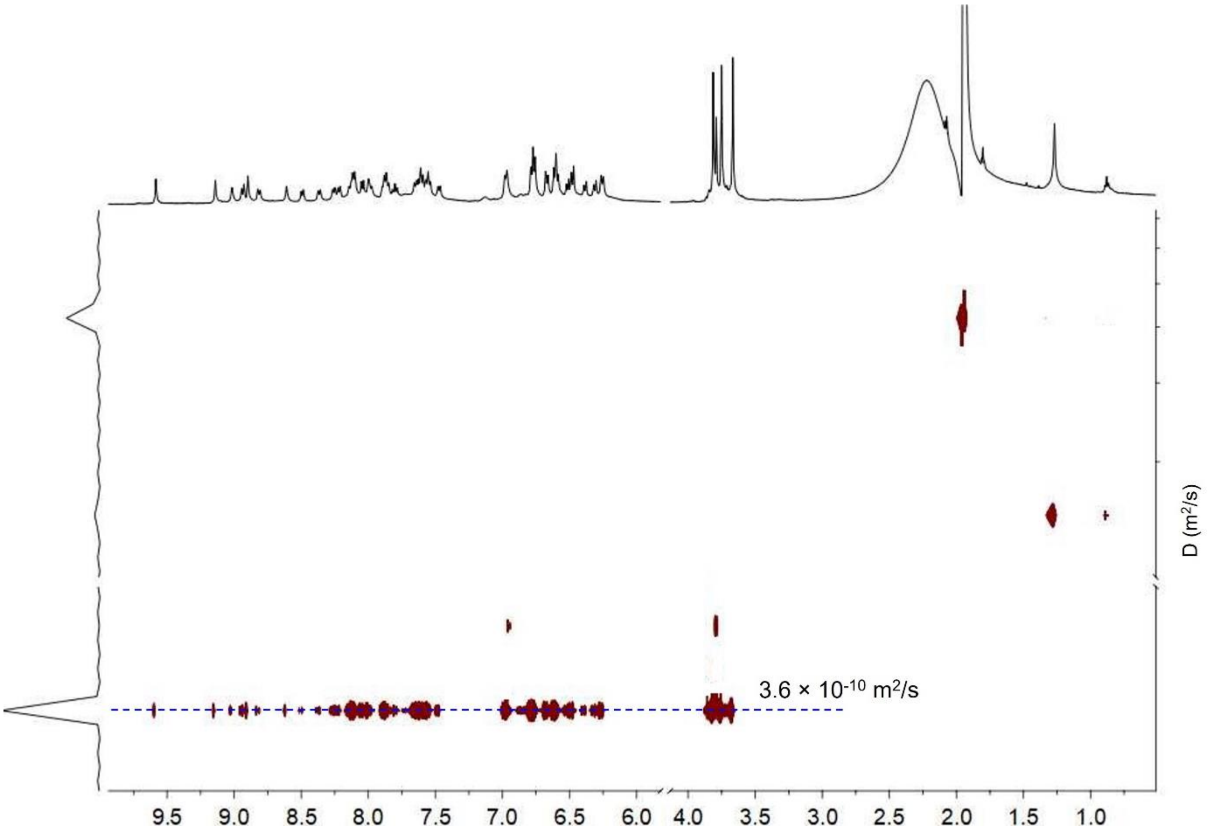

Figure S10. ^{1}H DOSY spectrum (500 MHz, 298 K, CD_3CN) of pseudo-icosahedron **1**. The diffusion coefficient of **1** in CD_3CN was measured to be 3.6×10^{-10} m^2 s^{-1}.

The Stokes-Einstein equation can generally be used to determine the effective radius of a spherical particle moving through a fluid by thermal motion (or diffusion).

$r = \frac{k_B T}{6\pi\eta D}$, where r is the effective radius (m), k_B is the Boltzmann constant (J K^{-1}), T is the temperature (K), η is the fluid viscosity (Pa s), and D is the diffusion coefficient of the particle (m^2 s^{-1}).

Following the Stokes-Einstein equation and with a CD_3CN viscosity of 3.41×10^{-4} Pa s,[3,4] the diffusion coefficient calculated following the DOSY experiment of Figure S10 corresponds to a solvodynamic radius of 1.8 nm for pseudo-icosahedron **1** (assuming perfect sphere behavior). This calculated solvodynamic radius is smaller than the corresponding radius of the PM7-optimized molecular structure of **1** (2.7 nm). We infer that this discrepancy may result from the porous, open-faced structure of pseudo-icosahedron **1**, which leads to a smaller contact surface area compared to the corresponding sphere. Therefore, such porous cage structures are expected to diffuse more rapidly than corresponding closed spheres, as we have observed previously.[4]

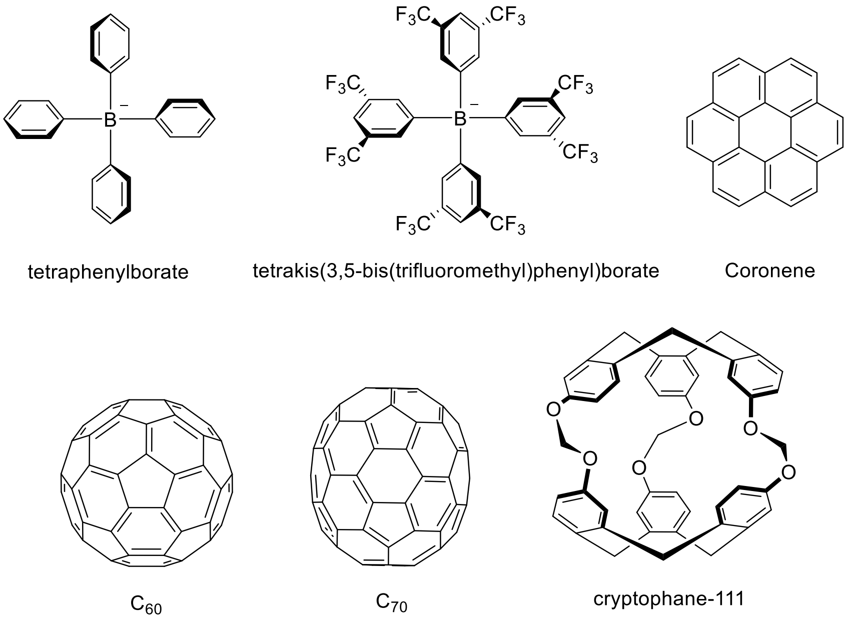

Scheme S3. Guests tested for pseudo-icosahedron **1** in acetonitrile.

4. Self-assembly and characterization of helicate 2

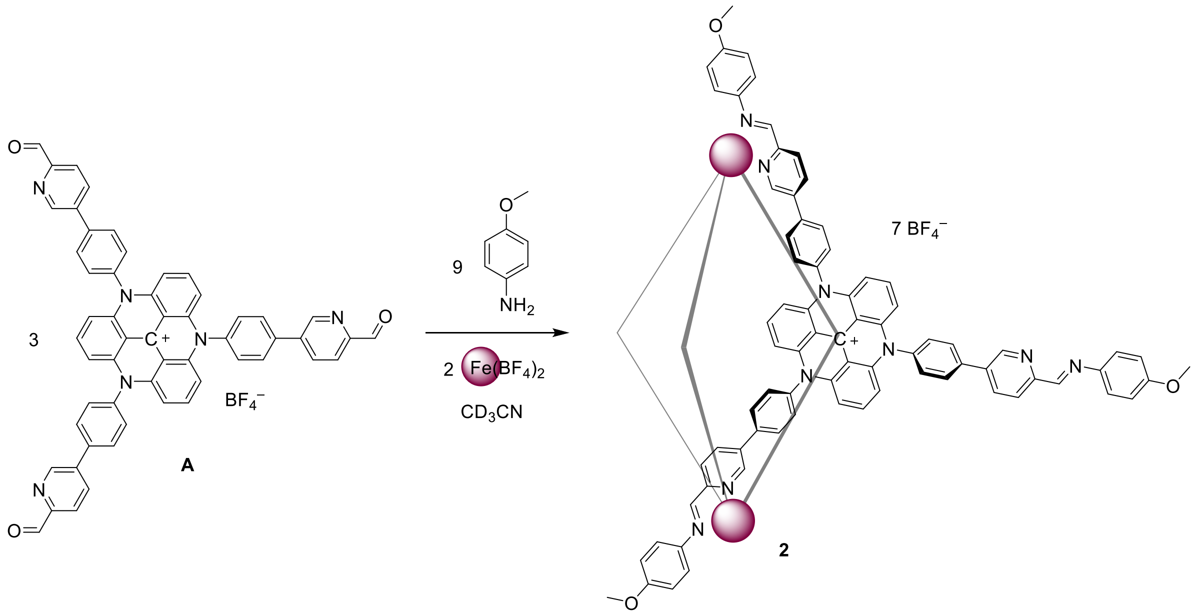

Scheme S4. Subcomponent self-assembly of helicate **2**.

Subcomponent **A** (2.0 mg, 2.2 μmol, 3.0 equiv), *p*-anisidine (0.81 mg, 6.6 μmol, 3.0 equiv), and $Fe(BF_4)_2 \cdot 6H_2O$ (0.39 mg, 1.1 μmol, 2.0 equiv) were combined in CH_3CN (1.0 mL) and stirred at 50 °C for two days. The solvent was partially evaporated and diethyl ether was then added. The precipitate was suspended and then centrifuged, and the diethyl ether was decanted. The residue was then dried in vacuo to afford the solid product as a red powder (2.8 mg, 92%). Note that helicate **2** could be also obtained when the self-assembly reaction was carried out in a microwave reactor at 120 °C for 3 h. **^{1}H NMR** (500 MHz, 298 K, CD_3CN): δ (ppm) 9.18-9.16 (m, 9H), 8.88 (d, J = 8.1 Hz, 6H), 8.76-8.74 (m, 9H), 8.37-8.23 (m, 15H), 8.17 (s, 6H), 7.75-7.63 (m, 27H), 7.56 (d, J = 6.95 Hz, 6H), 7.45-7.39 (m, 9H), 7.05 (d, J = 8.8 Hz, 6H), 6.90 (d, J = 8.9 Hz, 12H), 6.59-6.55 (m, 6H), 6.50 (d, J = 8.5 Hz, 6H), 6.33 (d, J = 8.5 Hz, 6H), 5.74 (d, J = 8.8 Hz, 12H), 3.88-3.87 (m, 27H). **^{13}C NMR** (125.8 MHz, 298 K, CD_3CN): δ (ppm) 174.1, 160.9, 160.2, 158.7, 158.6, 158.4, 155.7, 154.6, 153.2, 149.8, 149.1, 144.8, 143.2, 143.0, 142.6, 141.0, 140.1, 138.5, 138.4, 138.2, 137.0, 136.8, 136.2, 132.5, 132.2, 131.9, 131.8, 131.3, 130.7, 130.5, 130.4, 130.3, 129.2, 124.3, 123.8, 122.7, 122.0, 115.4(2), 115.3(5), 111.1, 108.3, 108.2, 108.1, 107.8, 56.5, 56.1. **^{19}F NMR** (470 MHz, 298 K, CD_3CN): δ (ppm) -152.1. **ESI-MS**: *m/z* 603.8 $[M+BF_4^-]^{6+}$, 742.0 $[M+2BF_4^-]^{5+}$, 949.2 $[M+3BF_4^-]^{4+}$, 1294.6 $[M+4BF_4^-]^{3+}$.

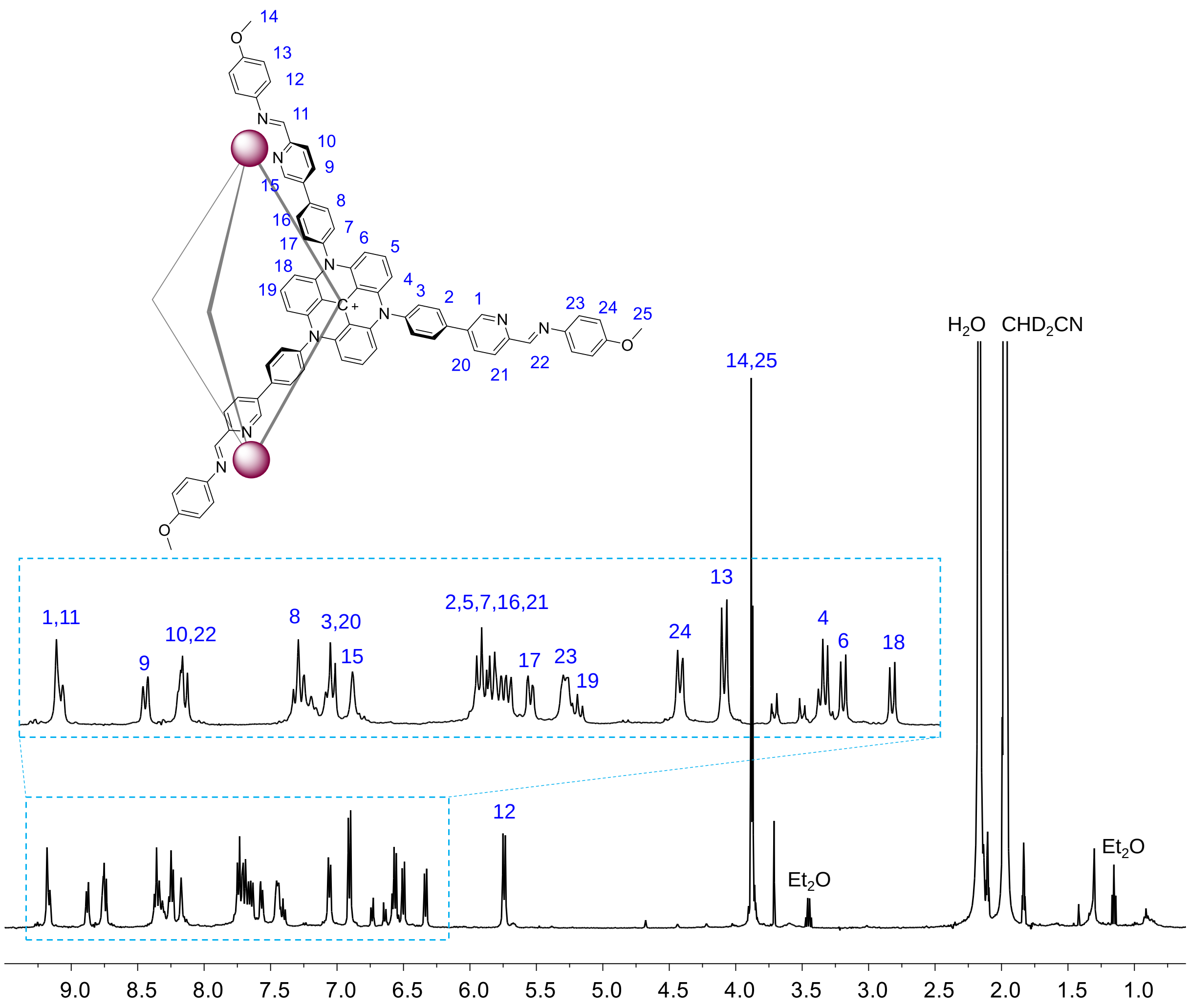

Figure S11. ^{1}H NMR spectrum (500 MHz, 298 K, CD_3CN) of helicate **2**.

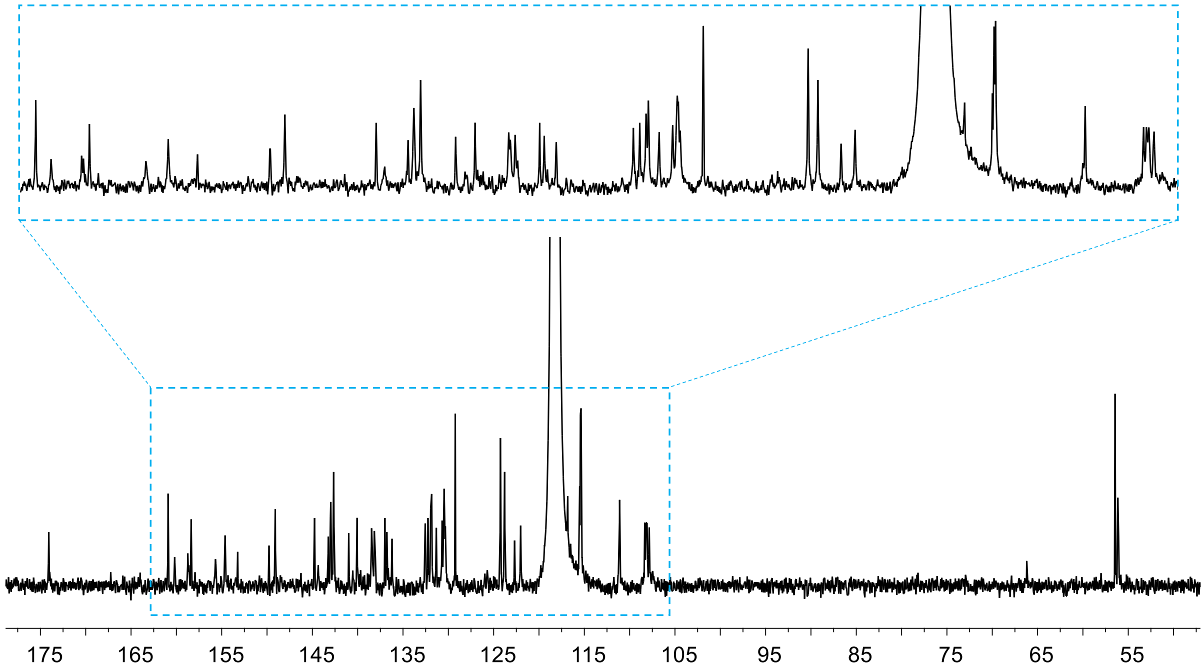

Figure S12. ^{13}C NMR spectrum (125.8 MHz, 298 K, CD_3CN) of helicate **2**.

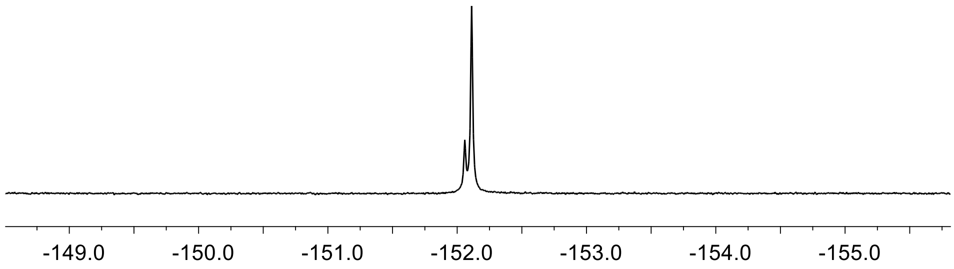

Figure S13. ^{19}F NMR spectrum (470 MHz, 298 K, CD_3CN) of helicate **2**.

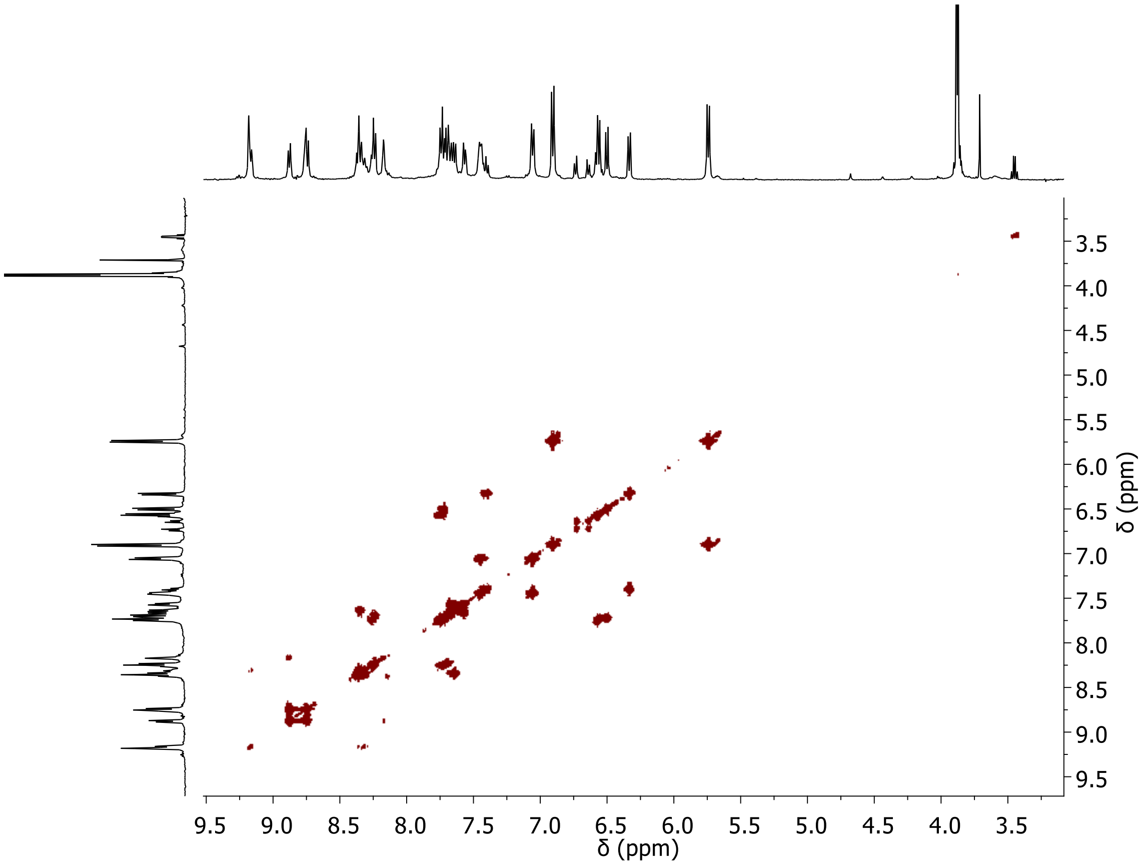

Figure S14. ^{1}H-^{1}H COSY spectrum (500 MHz, 298 K, CD_3CN) of helicate **2**.

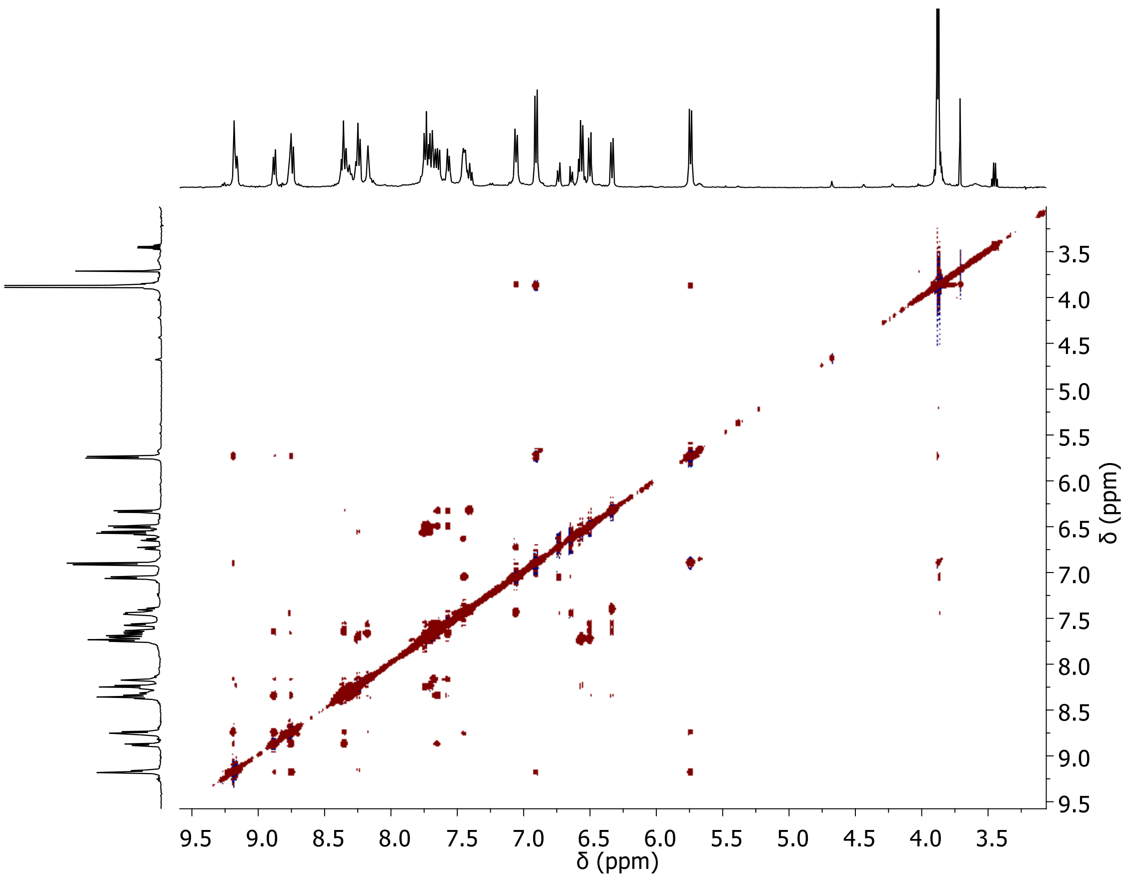

Figure S15. ^{1}H-^{1}H NOESY spectrum (500 MHz, 298 K, CD_3CN) of helicate **2**.

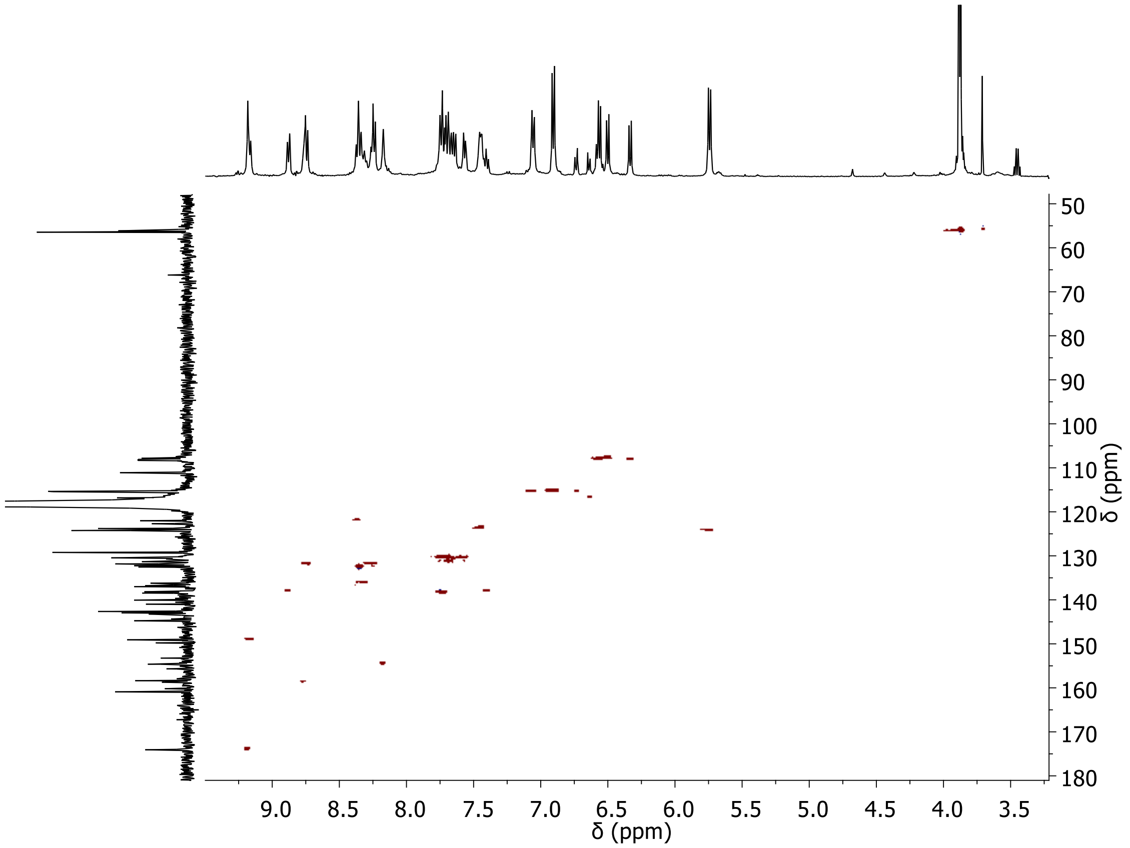

Figure S16. ^{1}H-^{13}C HSQC spectrum (500 MHz, 298 K, CD_3CN) of helicate **2**.

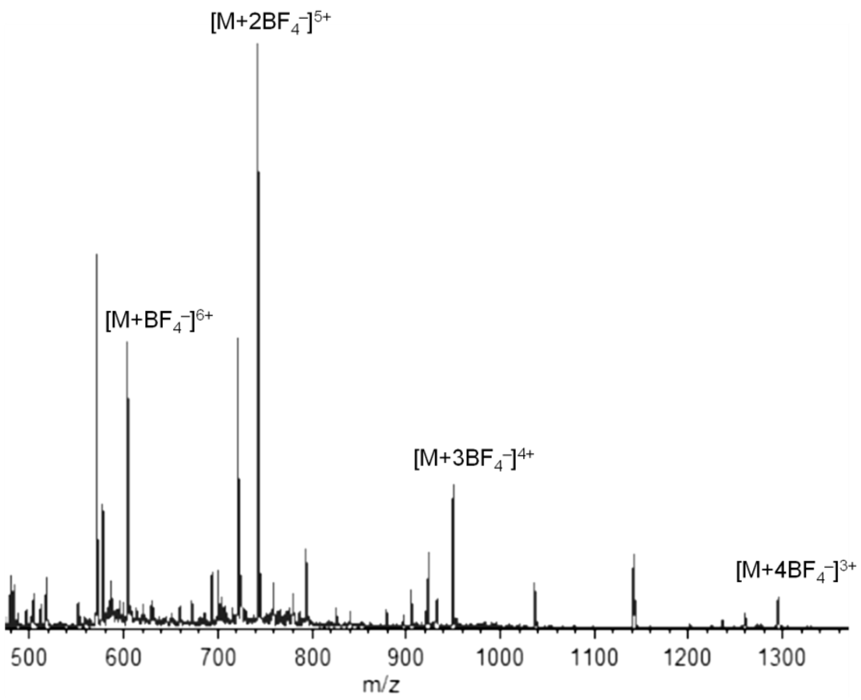

Figure S17. Low-resolution ESI-mass spectrum of helicate **2**.

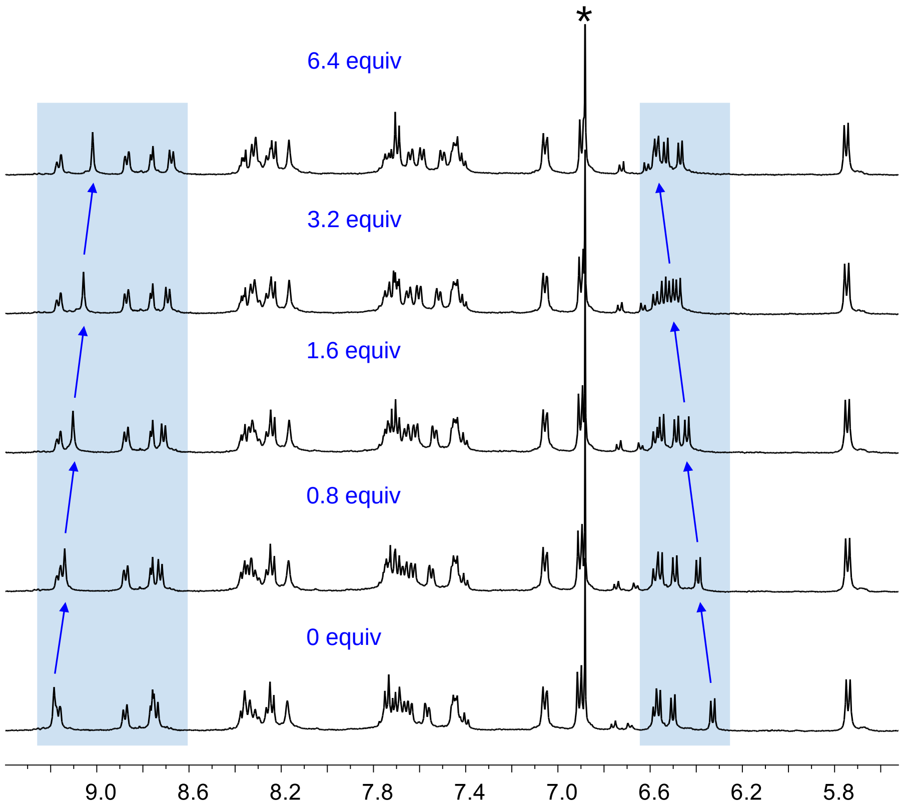

Figure S18. ^{1}H NMR spectra (500 MHz, 298 K, CD_3CN) of helicate **2** (0.073 mM, BF_4^- as the counterions) upon addition of different equivalents of tetrabutylammonium perchlorate ($TBA^+ClO_4^-$). The peak from the 1,4-dimethoxybenzene internal standard is indicated by an asterisk.

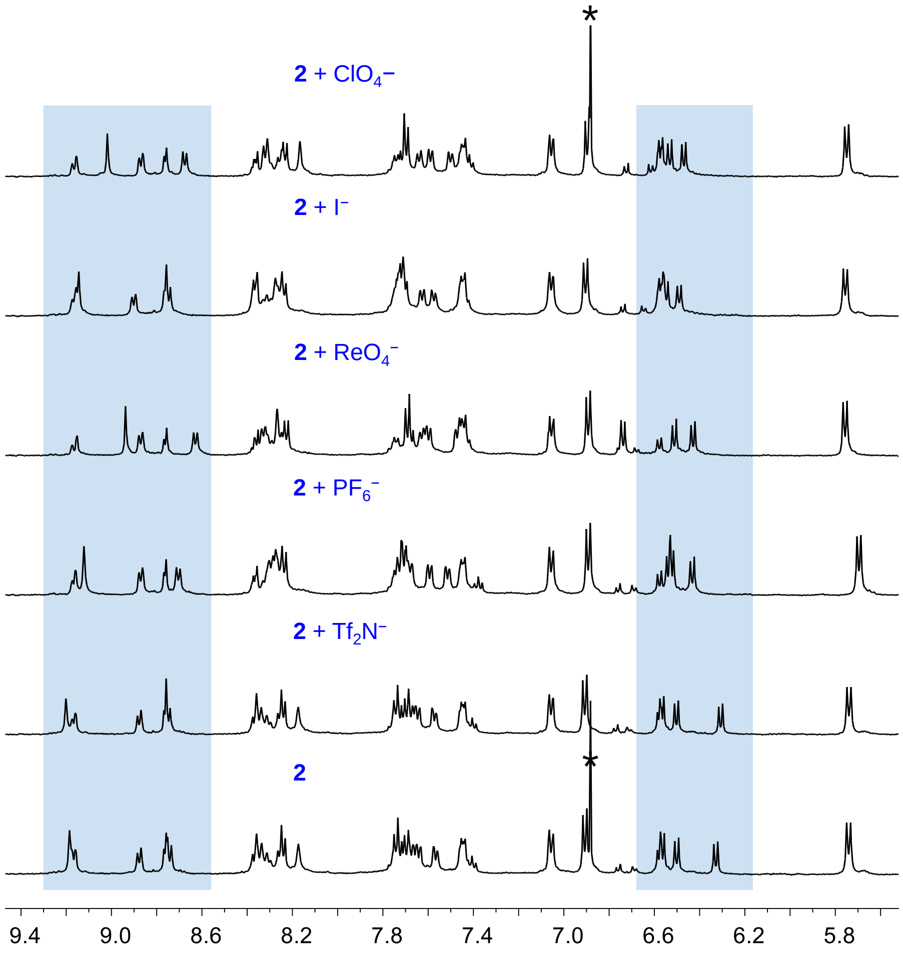

Figure S19. ^{1}H NMR spectra (500 MHz, 298 K, CD_3CN) of helicate **2** (0.073 mM, BF_4^- as the counterions) upon addition of an excess of various anions (> 5 equiv, tetrabutylammonium salts). The peak from the 1,4-dimethoxybenzene internal standard is indicated by an asterisk.

5. Self-assembly and characterization of helicate 2'

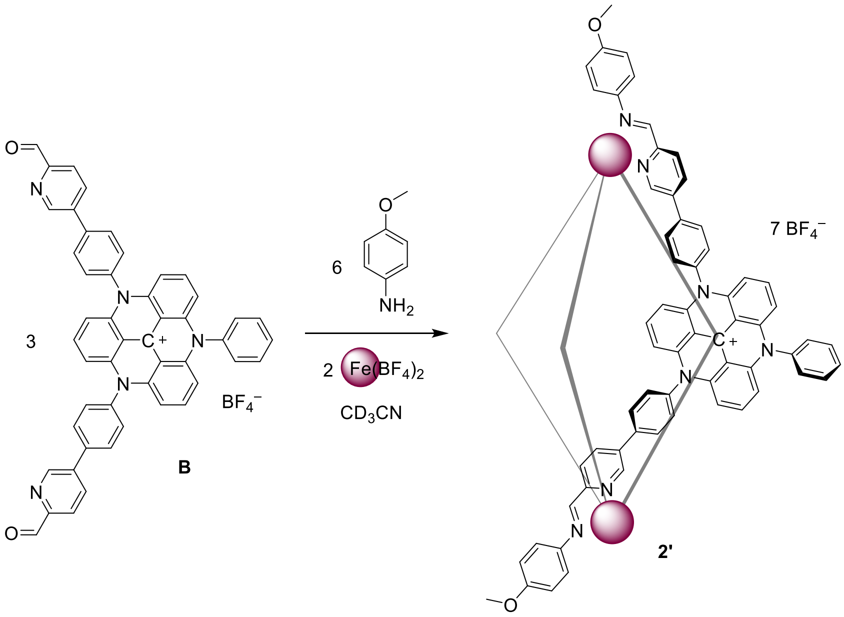

Scheme S5. Subcomponent self-assembly of helicate **2'**.

Subcomponent **B** (2.0 mg, 2.5 μmol, 3.0 equiv), *p*-anisidine (0.64 mg, 5.2 μmol, 6.3 equiv), and $Fe(BF_4)_2 \cdot 6H_2O$ (0.59 mg, 1.7 μmol, 2.1 equiv) were combined in CH_3CN (0.5 mL) and stirred at 50 °C for two days. The solvent was partially evaporated and diethyl ether was then added. The precipitate was suspended and then centrifuged, and the diethyl ether was decanted. This was repeated three times with fresh diethyl ether. The residue was then dried in vacuo to afford the solid product as a red powder (2.6 mg, 90%). **^{1}H NMR** (500 MHz, 298 K, CD_3CN): δ (ppm) 9.15 (s, 6H), 8.85 (d, J = 8.0 Hz, 6H), 8.72 (d, J = 8.1 Hz, 6H), 8.32 (d, J = 8.2 Hz, 6H), 8.14 (s, 6H), 7.86 (d, J = 7.7 Hz, 6H), 7.79 (t, J = 7.4 Hz, 3H), 7.67-7.64 (m, 12H), 7.59 (d, J = 1.7 Hz, 6H), 7.54-7.51 (m, 12H), 7.36 (t, J = 8.4 Hz, 3H), 6.88 (d, J = 8.7 Hz, 12H), 6.45-6.42 (m, 12H), 6.29 (d, J = 8.5 Hz, 6H), 5.72 (d, J = 8.6 Hz, 12H), 3.86 (s, 18H). **^{13}C NMR** (125.8 MHz, 298 K, CD_3CN): δ (ppm) 174.1, 160.9, 158.4, 154.6, 144.8, 143.2, 143.0, 142.6(3), 142.5(9), 141.0, 140.1, 138.8, 138.4, 138.2, 138.0, 137.0, 133.2, 132.5, 132.0, 131.6, 131.3, 130.7, 130.5, 129.4, 124.3, 115.4, 111.1, 111.0, 108.3, 108.2, 107.7, 56.4. **^{19}F NMR** (470 MHz, 298K, CD_3CN): δ (ppm) -151.7. **ESI-MS**: *m/z* 415.0 $[M]^{7+}$, 498.7 $[M+BF_4^-]^{6+}$, 615.8 $[M+2BF_4^-]^{5+}$, 791.5 $[M+3BF_4^-]^{4+}$.

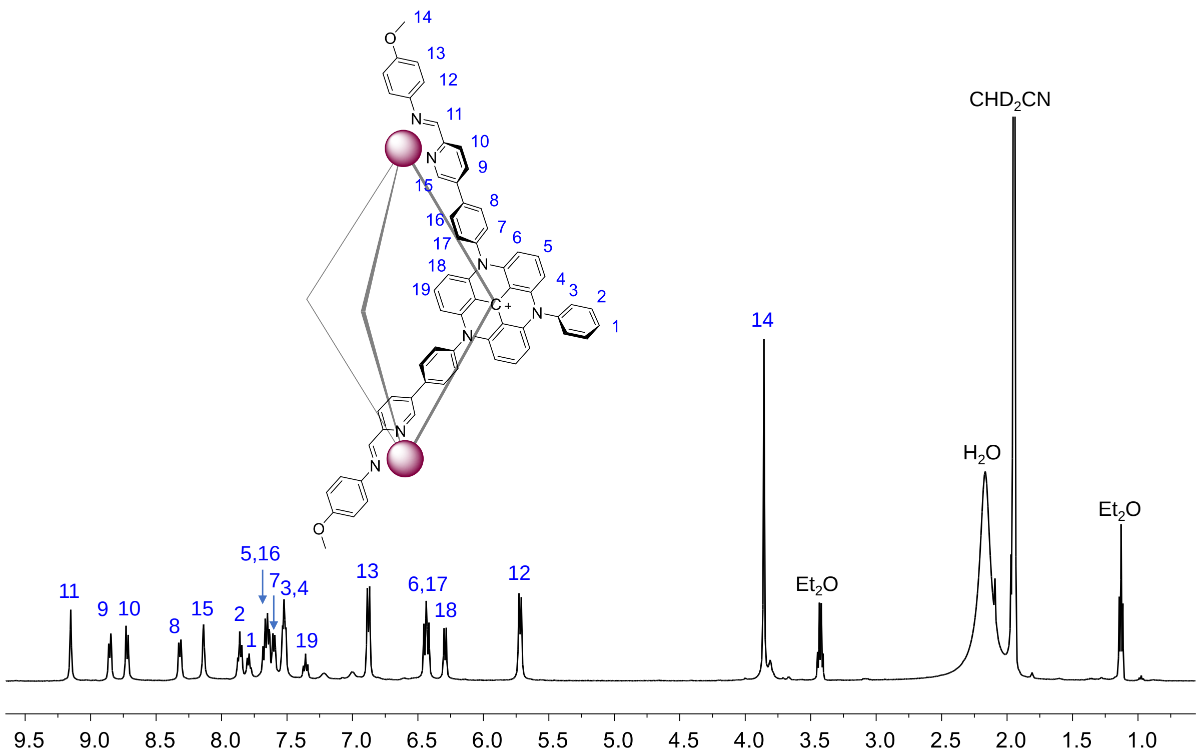

Figure S20. ^{1}H NMR spectrum (500 MHz, 298 K, CD_3CN) of helicate **2'**.

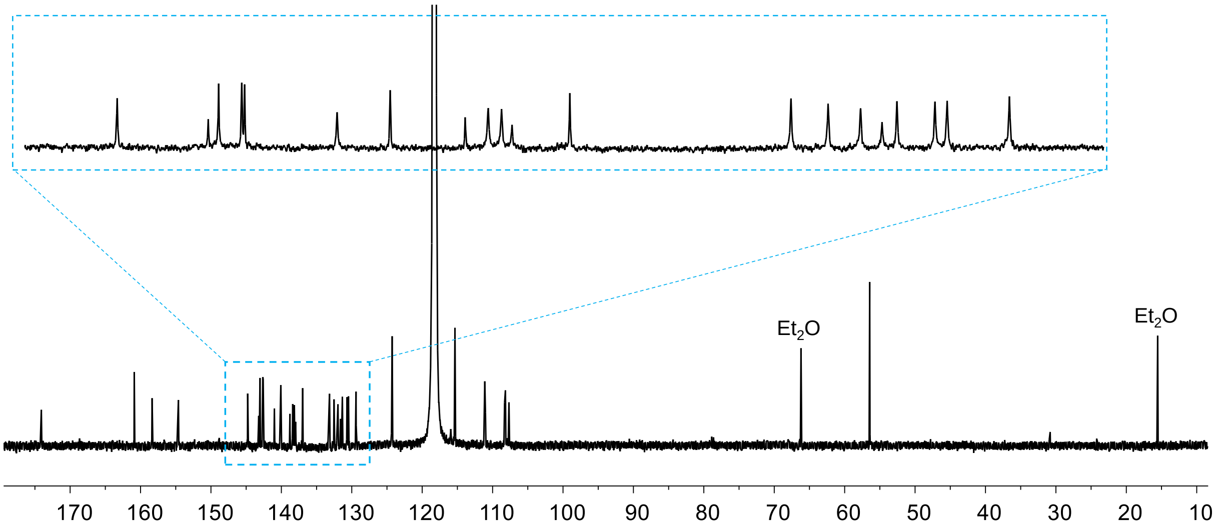

Figure S21. ^{13}C NMR spectrum (125.8 MHz, 298 K, CD_3CN) of helicate **2'**.

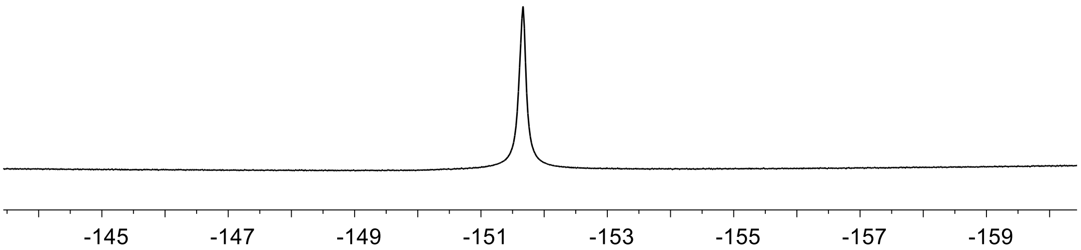

Figure S22. ^{19}F NMR spectrum (470 MHz, 298 K, CD_3CN) of helicate **2'**.

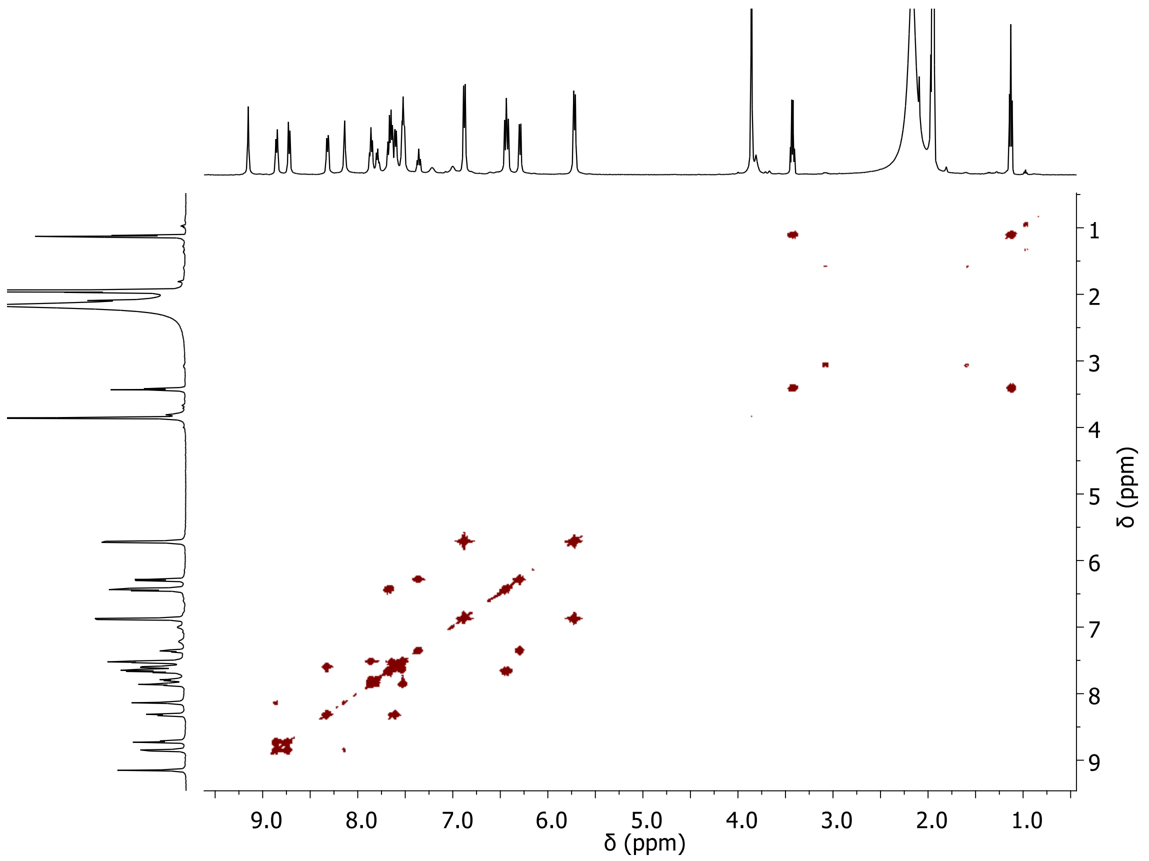

Figure S23. ^{1}H-^{1}H COSY spectrum (500 MHz, 298 K, CD_3CN) of helicate **2'**.

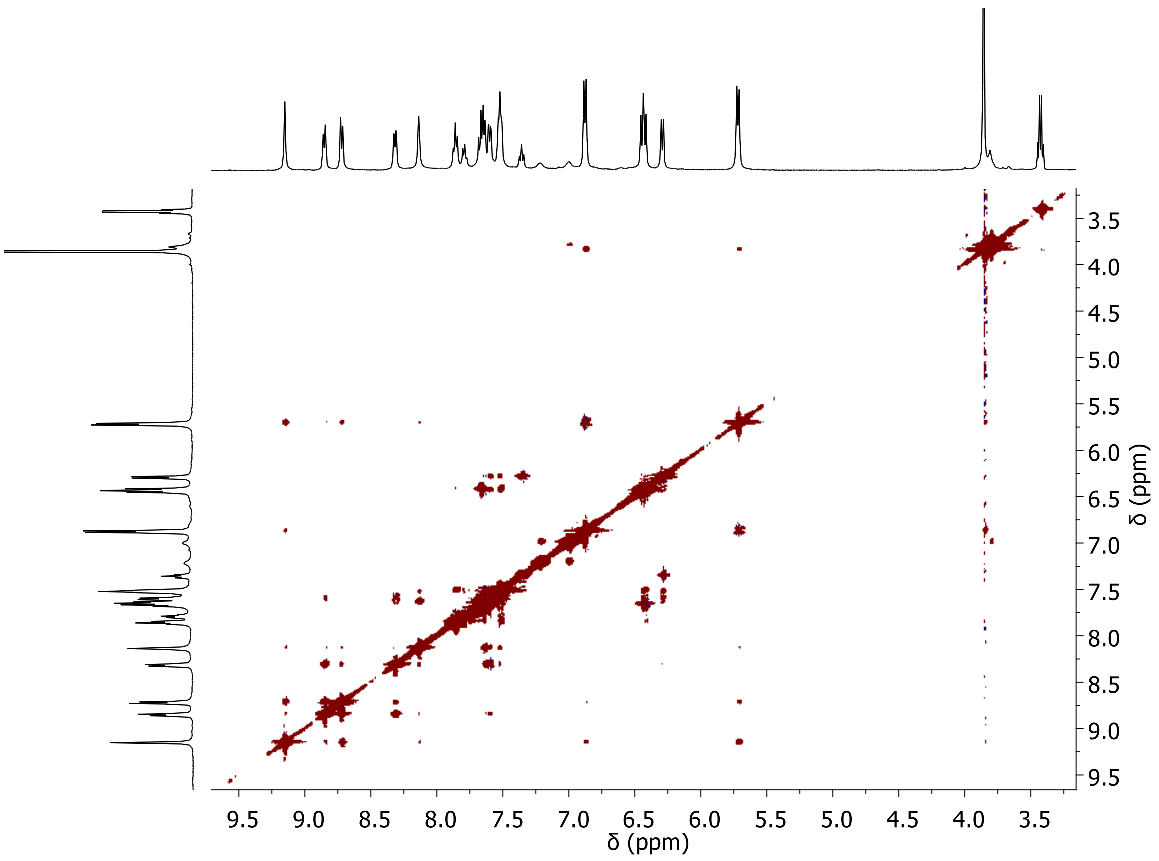

Figure S24. ^{1}H-^{1}H NOESY spectrum (500 MHz, 298 K, CD_3CN) of helicate **2'**.

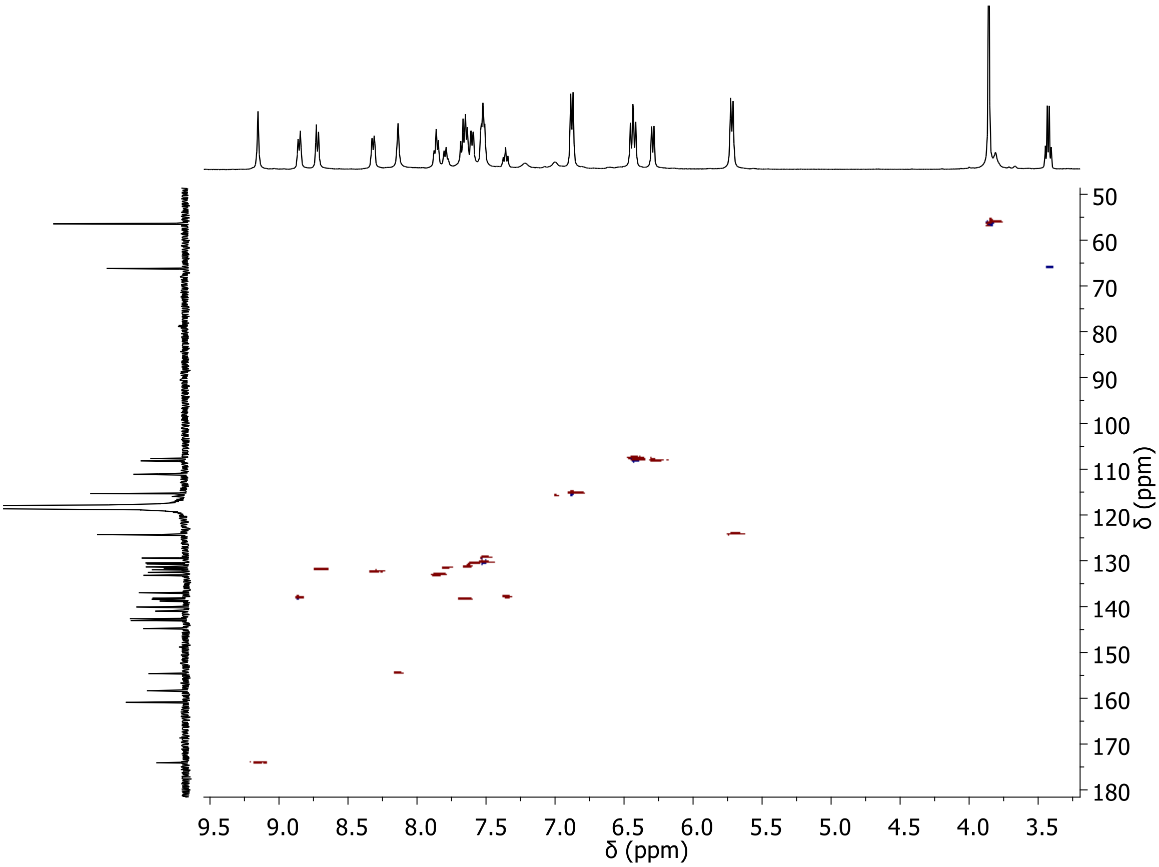

Figure S25. ^{1}H-^{13}C HSQC spectrum (500 MHz, 298 K, CD_3CN) of helicate **2'**.

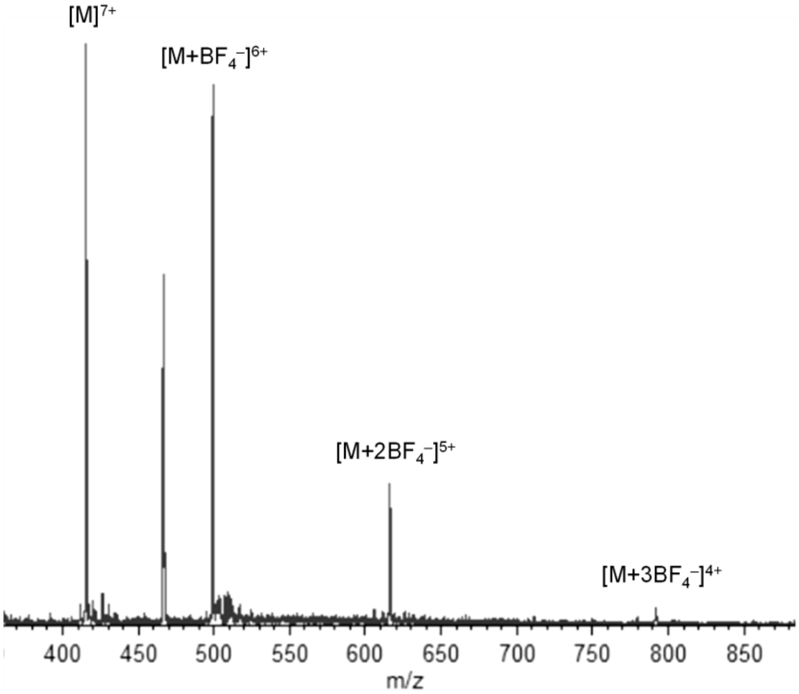

Figure S26. Low-resolution ESI-mass spectrum of helicate **2'**.

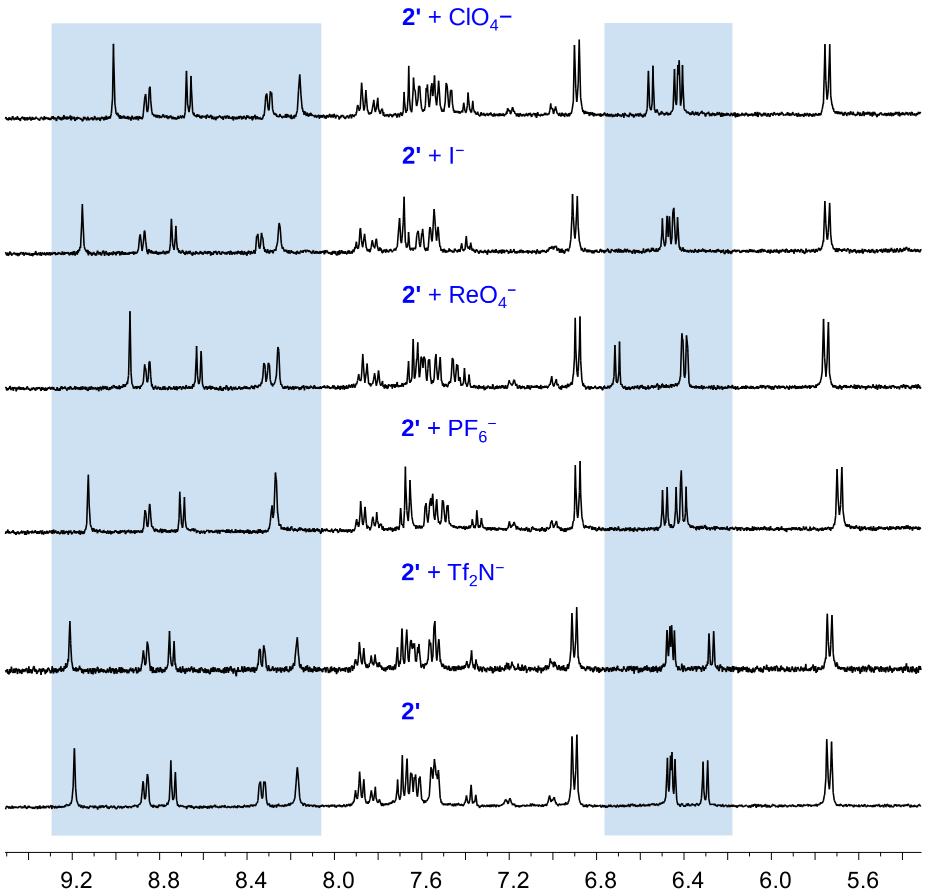

Figure S27. ^{1}H NMR spectra (500 MHz, 298 K, CD_3CN) of helicate **2'** (0.073 mM, BF_4^- as the counterions) upon addition of an excess of various anions (> 5 equiv, tetrabutylammonium salts).

6. Self-assembly and characterization of tetrahedron 3

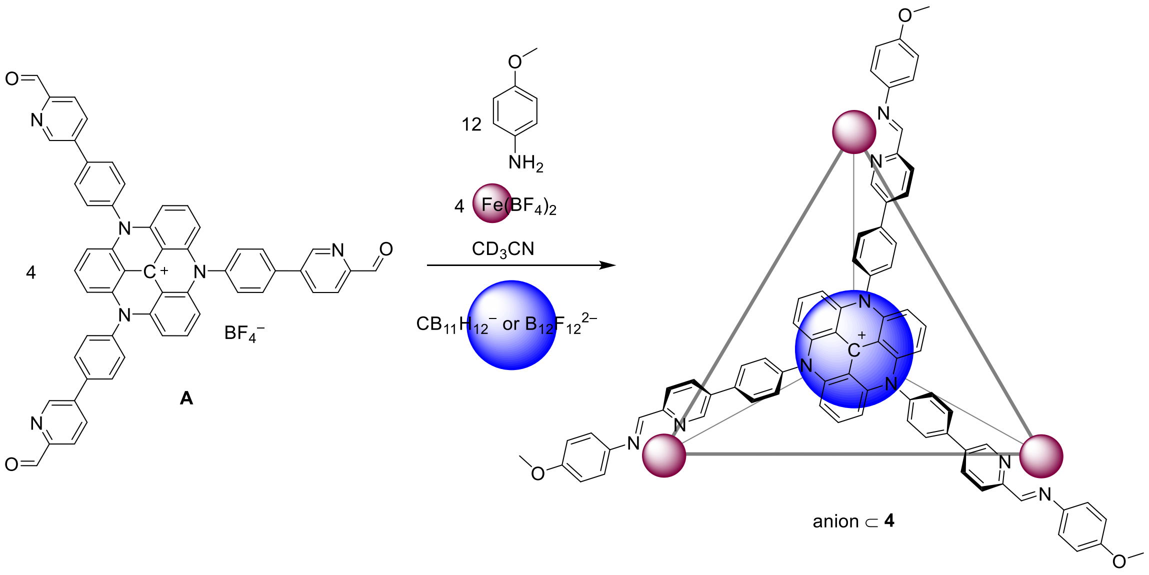

Scheme S6. Subcomponent self-assembly of tetrahedron **3** in the presence of $CB_{11}H_{12}^{-}$ or $B_{12}F_{12}^{2-}$.

To the reaction mixture of pseudo-icosahedron **1** or helicate **2** in CD_3CN, solid $CsCB_{11}H_{12}$ or $K_2B_{12}F_{12}$ (> 2 equiv) were added directly and the solution was stirred at 50 °C overnight. The red reaction mixture produced $CB_{11}H_{12}^{-}\subset$**3** or $B_{12}F_{12}^{2-}\subset$**3** in a clean form in solution without further purification.

$CB_{11}H_{12}^{-}\subset$**3**: **^{1}H NMR** (500 MHz, 298 K, CD_3CN): δ (ppm) 10.59 (bs, 12H), 9.08 (d, J = 7.6 Hz, 12H), 8.89 (bs, 12H), 8.46 (d, J = 7.9 Hz, 12H), 8.04 (d, J = 7.9 Hz, 12H), 7.67 (d, J = 8.4 Hz, 12H), 7.43 (t, J = 8.5 Hz, 12H), 7.23 (d, J = 7.7 Hz, 12H), 7.05-7.02 (m, 36H), 6.39 (d, J = 8.5 Hz, 12H), 6.28 (d, J = 8.4 Hz, 12H), 5.71 (d, J = 7.9 Hz, 24H), 3.92 (s, 36H). **^{19}F NMR** (470 MHz, 298 K, CD_3CN): δ (ppm) -151.4. **ESI-MS**: m/z 940.7 $[M+6BF_4^{-}]^{6+}$, 931.3 $[M+CB_{11}H_{12}^{-}+5BF_4^{-}]^{6+}$, 922.3 $[M+2CB_{11}H_{12}^{-}+4BF_4^{-}]^{6+}$, 913.0 $[M+3CB_{11}H_{12}^{-}+3BF_4^{-}]^{6+}$, 903.7 $[M+4CB_{11}H_{12}^{-}+2BF_4^{-}]^{6+}$, 894.3 $[M+5CB_{11}H_{12}^{-}+1BF_4^{-}]^{6+}$, 883.5 $[M+6CB_{11}H_{12}^{-}]^{6+}$.

$B_{12}F_{12}^{2-}\subset$**3**: **^{1}H NMR** (500 MHz, 298 K, CD_3CN): δ (ppm) 10.31 (bs, 12H), 8.98 (d, J = 7.7 Hz, 12H), 8.44-8.43 (m, 24H), 7.93 (d, J = 8.0 Hz, 12H), 7.64 (d, J = 7.8 Hz, 12H), 7.33-7.27 (m, 24H), 7.14 (d, J = 8.1 Hz, 12H), 7.00 (d, J = 8.2 Hz, 24H), 6.28 (d, J = 8.5 Hz, 12H), 6.12 (d, J = 8.5 Hz, 12H), 5.77 (d, J = 8.0 Hz, 24H), 3.92 (s, 36H). **^{19}F NMR** (470 MHz, 298 K, CD_3CN): δ (ppm) -151.4, -268.8, -275.0. **ESI-MS**: m/z 1116.1 $[M+B_{12}F_{12}^{2-}+5BF_4^{-}]^{5+}$, 915.6 $[M+B_{12}F_{12}^{2-}+4BF_4^{-}]^{6+}$, 772.4 $[M+B_{12}F_{12}^{2-}+3BF_4^{-}]^{7+}$, 664.9 $[M+B_{12}F_{12}^{2-}+2BF_4^{-}]^{8+}$, 581.4 $[M+B_{12}F_{12}^{2-}+BF_4^{-}]^{9+}$.

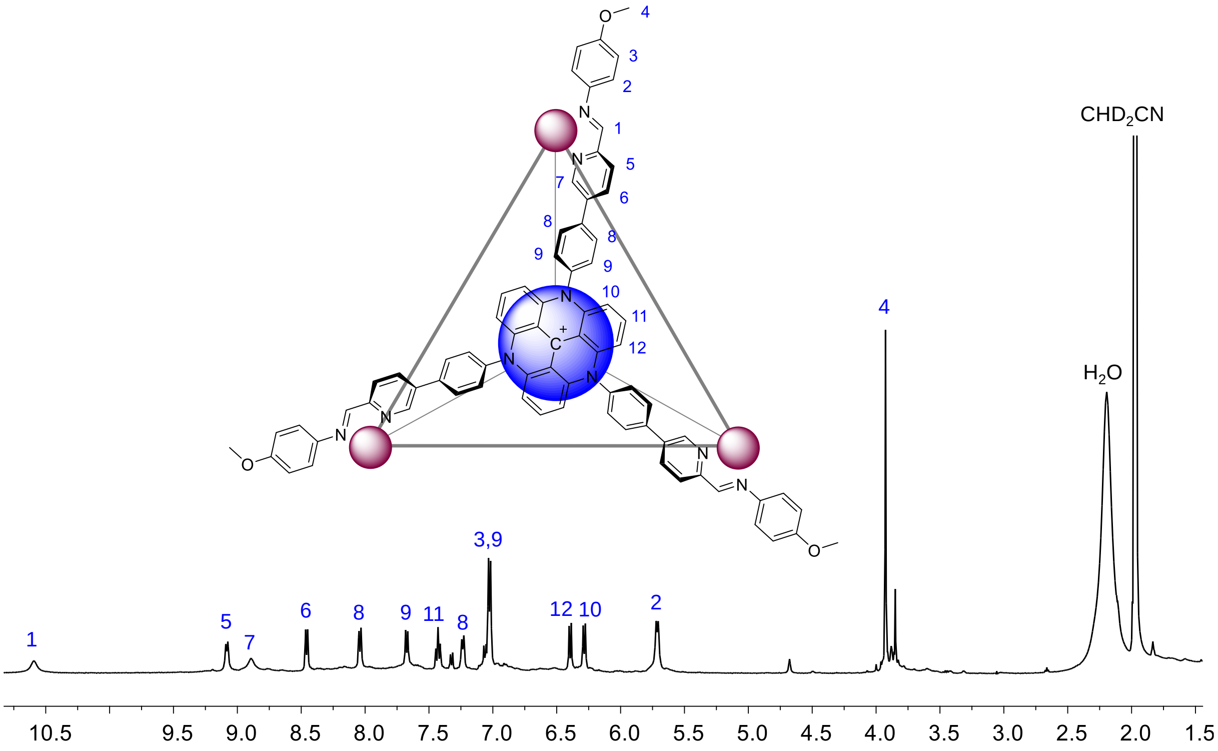

Figure S28. ^{1}H NMR spectrum (500 MHz, 298 K, CD_3CN) of $CB_{11}H_{12}^{-}\subset$**3**.

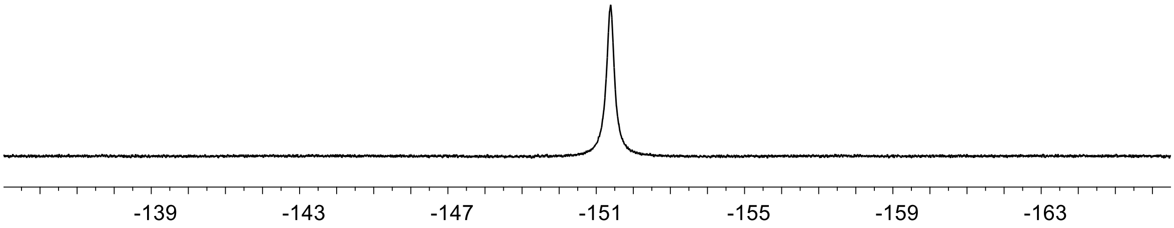

Figure S29. ^{19}F NMR spectrum (470 MHz, 298 K, CD_3CN) of $CB_{11}H_{12}^{-}\subset$**3**.

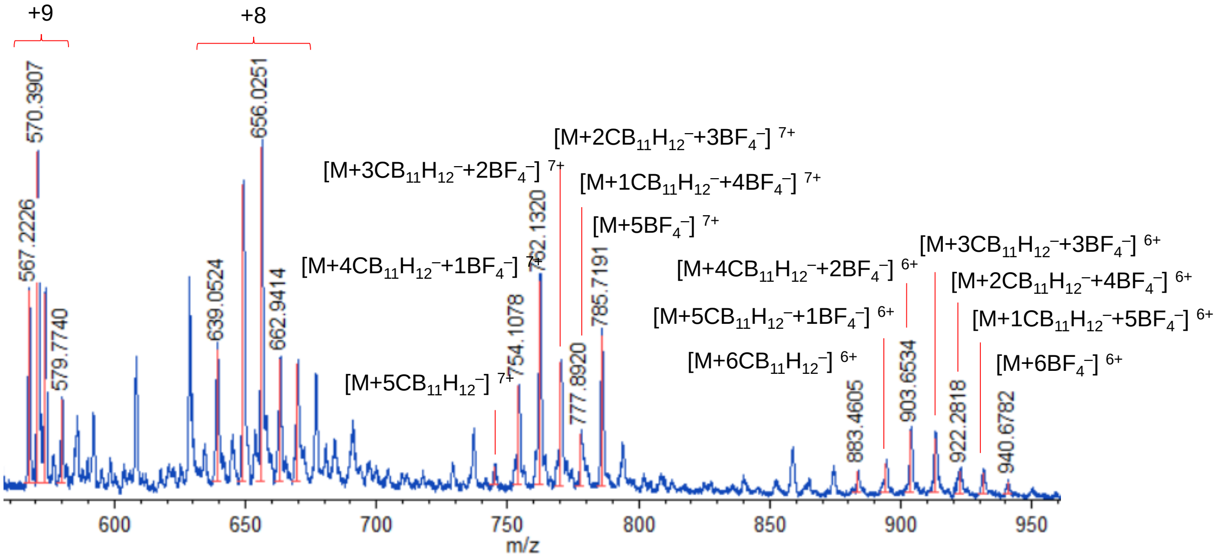

Figure S30. Low-resolution ESI-mass spectrum of $CB_{11}H_{12}^{-}\subset$**3**.

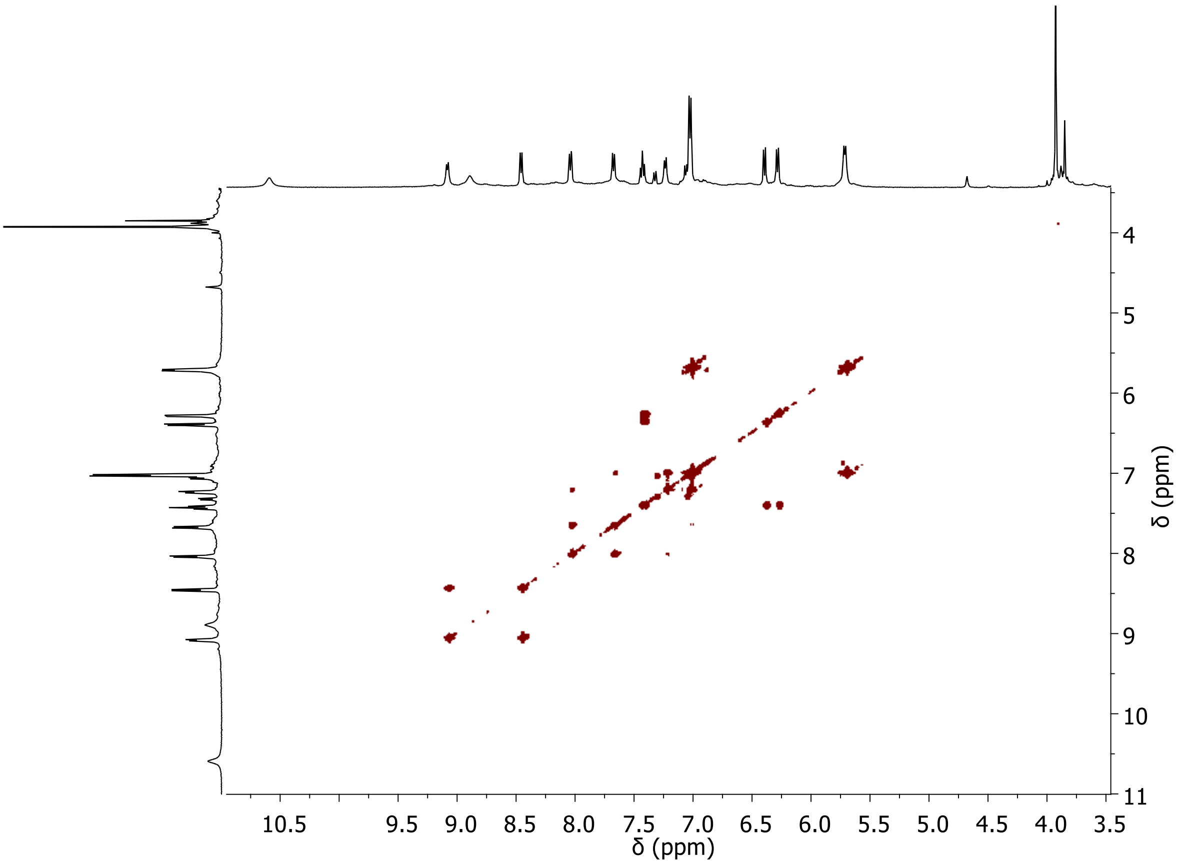

Figure S31. ^{1}H-^{1}H COSY spectrum (500 MHz, 298 K, CD_3CN) of $CB_{11}H_{12}^{-}\subset$**3**.

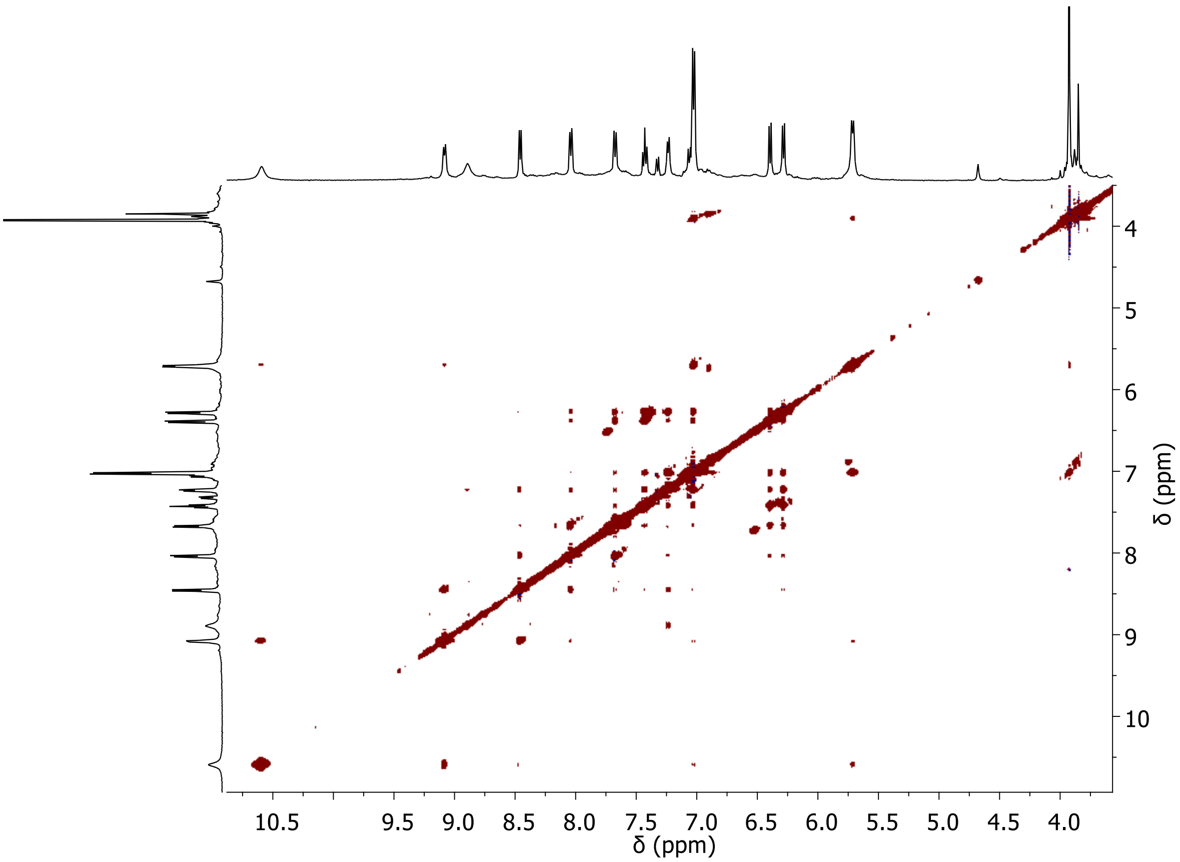

Figure S32. ^{1}H-^{1}H NOESY spectrum (500 MHz, 298 K, CD_3CN) of $CB_{11}H_{12}^{-}\subset$**3**.

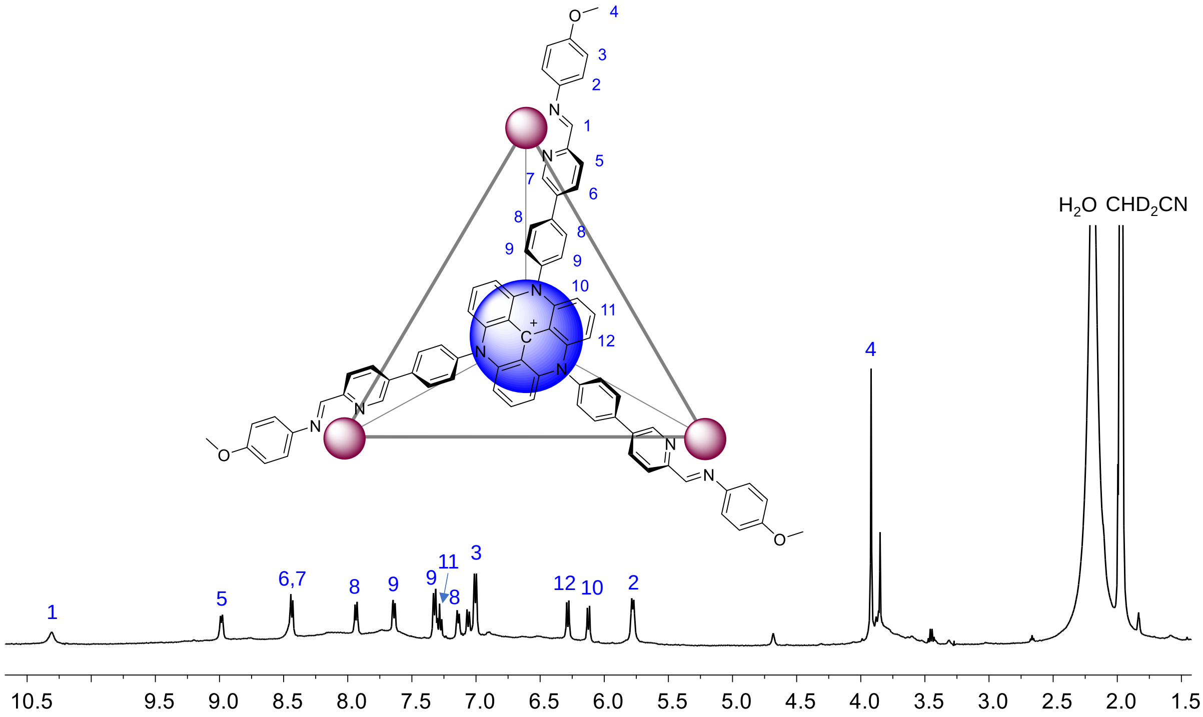

Figure S33. ^{1}H NMR spectrum (500 MHz, 298 K, CD$_3$CN) of $B_{12}F_{12}^{2-}\subset$**3**.

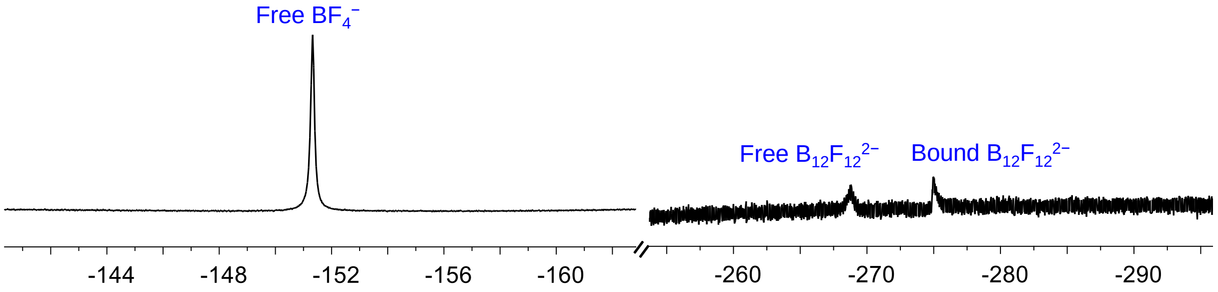

Figure S34. ^{19}F NMR spectrum (470 MHz, 298 K, CD$_3$CN) of $B_{12}F_{12}^{2-}\subset$**3** with the region from -250 to -300 ppm magnified.

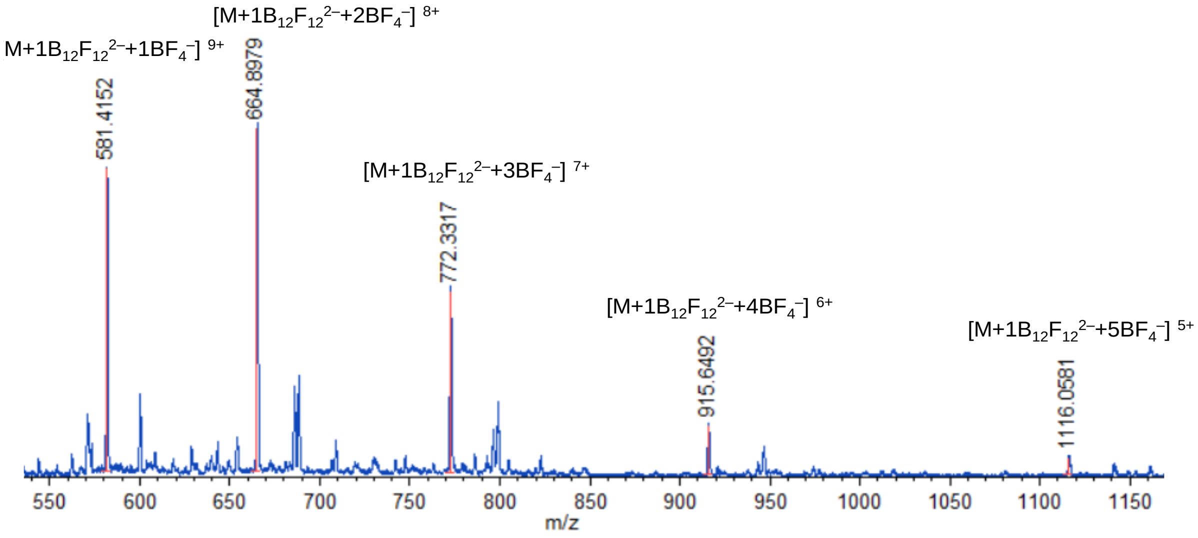

Figure S35. Low-resolution ESI-mass spectrum of $B_{12}F_{12}^{2-}\subset$**3**.

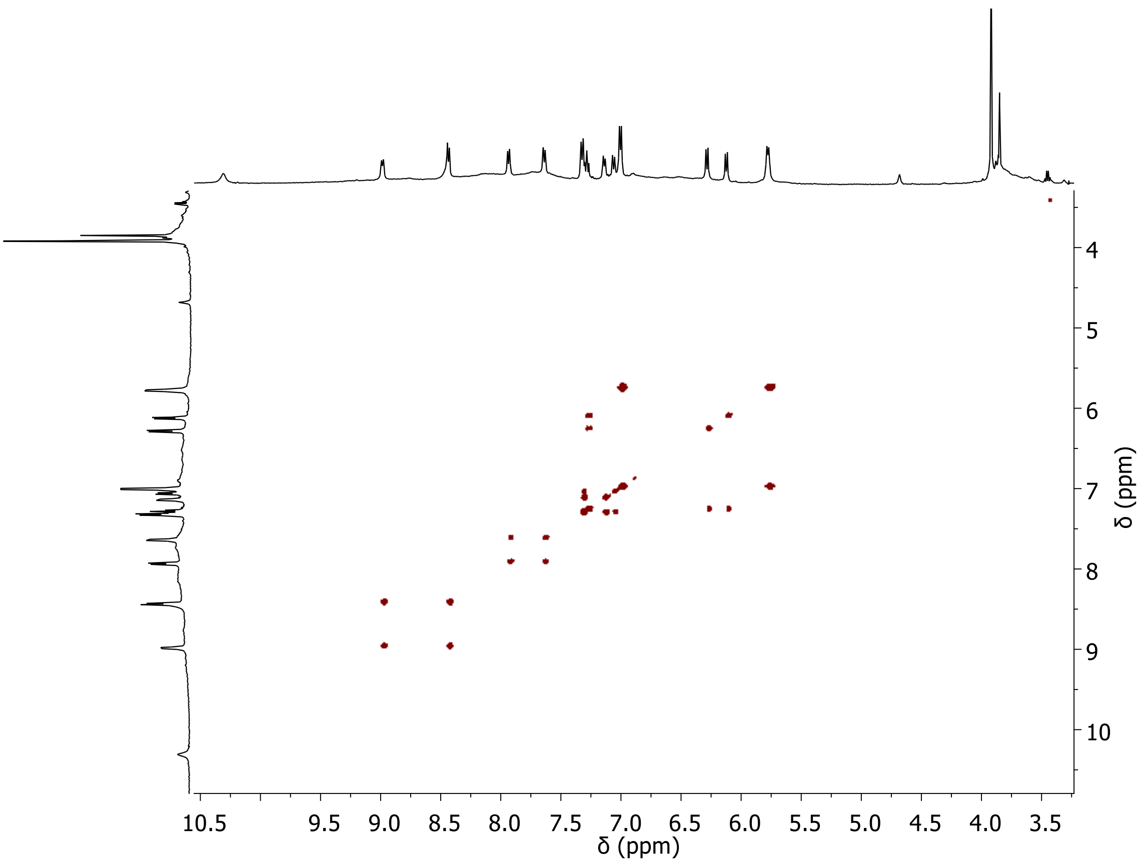

Figure S36. ^1H-^1H COSY spectrum (500 MHz, 298 K, CD_3CN) of $B_{12}F_{12}^{2-}\subset$**3**.

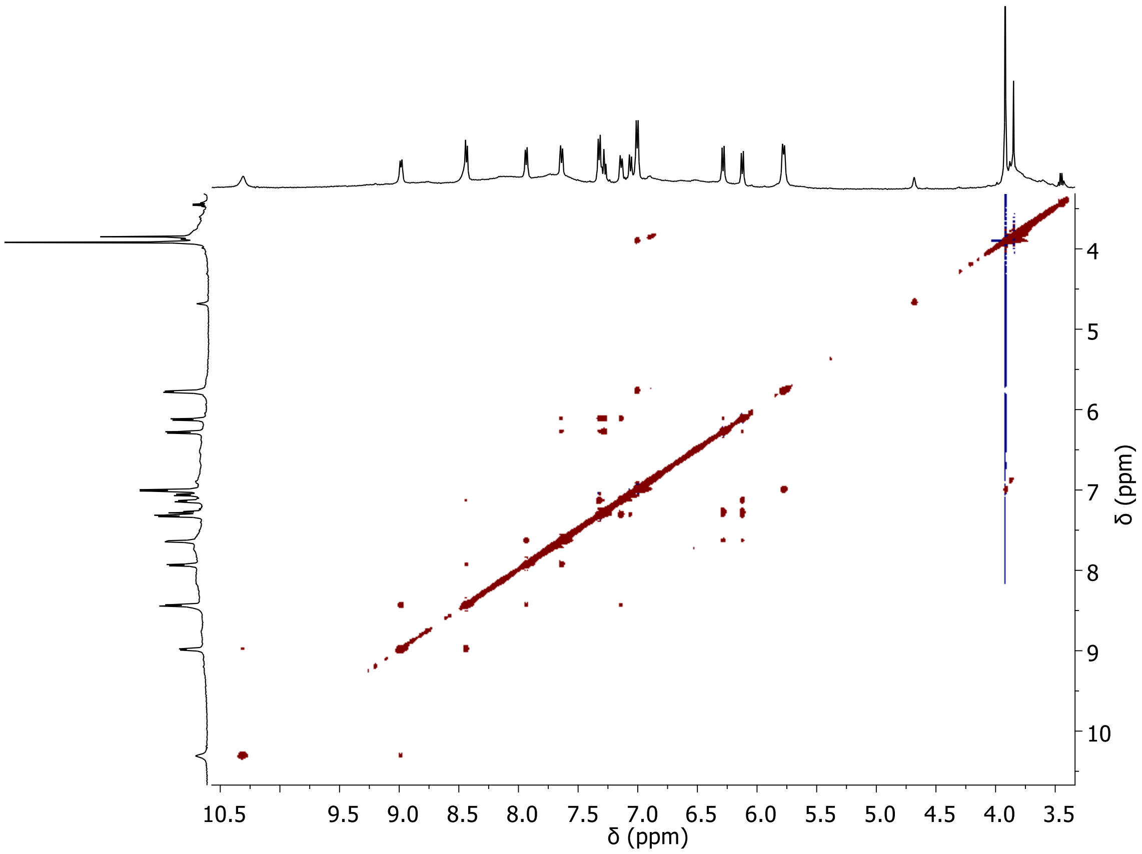

Figure S37. ^1H-^1H NOESY spectrum (500 MHz, 298 K, CD_3CN) of $B_{12}F_{12}^{2-}\subset$**3**.

7. X-ray crystallography

The crystals of **2'**·2.6ReO$_4$·4.4BF$_4$·3MeCN [+ solvent] were grown by diffusion of diethyl ether into an acetonitrile solution of **2'**·7BF$_4$ containing an excess of TBAReO$_4$ (> 10 equiv). Data were collected at Beamline I19 of Diamond Light Source employing silicon double crystal monochromated synchrotron radiation (0.6889 Å) with ω and ψ scans at 100(2) K.[5] Data integration and reduction were undertaken with Xia2.[6,7] Subsequent computations were carried out using the WinGX-32 graphical user interface.[8] A multi-scan empirical absorption correction using spherical harmonics was applied to the data using DIALS.[7] The structure was solved by intrinsic phasing using SHELXT[9] then refined and extended with SHELXL.[10] Carbon-bound hydrogen atoms were included in idealised positions and refined using a riding model. Disorder was modelled using standard crystallographic methods including constraints, restraints and rigid bodies where necessary.

The crystals employed immediately lost solvent after removal from the mother liquor and rapid handling prior to flash cooling in liquid nitrogen was required to collect data. The crystals were very small and weakly diffracting and were subject to rapid beam damage during data collection using synchrotron radiation. Consequently few reflections at greater than 1.2 Å resolution were observed and the quality of the integration is less than ideal. Furthermore there was a significant drop-off in diffraction intensity after around 1.5 Å resolution resulting in a low ratio of observed/unique reflections. Nevertheless, the quality of the data is far more than sufficient to establish the connectivity of the structure. The asymmetric unit was found to contain one complete Fe$_2$L$_3$ assembly as well as associated counterions and solvent molecules.

Due to the limited resolution, bond lengths and angles within pairs of chemically identical organic ligands were restrained to be similar to each other. Additional DFIX and DANG restraints were applied to some areas of the structure displaying high degrees of thermal motion and some phenyl rings were modelled as rigid groups (AFIX 66). Due to the limited data to parameter ratio only iron and rhenium were refined anisotropically and thermal parameter restraints (SIMU) were applied to all the lighter atoms. However even with these restraints some thermal parameters remain higher than ideal indicative of thermal motion or minor unresolved disorder.

The perrhenate and tetrafluoroborate anions were restrained to be approximately tetrahedral. Two peripheral anions were modelled as a disordered mixture of perrhenate and tetrafluoroborate anions. Their occupancies were first refined and then fixed at the obtained

values. Most additional peaks or holes in the electron density map are located close to the anions, indicative of further unresolved disorder or possibly absorption effects related to the presence of rhenium. No satisfactory model for two remaining anions (included as tetrafluoroborate in the formula due to the absence of any large electron density peaks for rhenium) could be found despite numerous attempts at modelling. Consequently the SQUEEZE[11] function of PLATON[12] was employed to remove the contribution of the electron density associated with these anions and further highly disordered solvent, which gave a potential solvent accessible void of 5516 Å^3 per unit cell (a total of approximately 1631 electrons).

CheckCIF gives 2 level A alerts and 3 B level alerts. These alerts all result from the limited resolution of the data (low resolution, low bond precision), isotropic modelling and the thermal motion within the structure (large C Ueq(max)/Ueq(min) range) as described above.

Crystallographic data have been deposited with the CCDC (2071272).

Formula $C_{195}H_{141}B_{4.40}F_{17.60}Fe_2N_{24}O_{16.40}Re_{2.60}$, M 4060.49, Monoclinic, space group P 21/n (#14), a 28.2808(9), b 21.5128(3), c 36.7005(9) Å, β 112.219(2), V 20670.5(9) Å^3, D_c 1.305 g cm^{-3}, Z 4, crystal size 0.015 by 0.010 by 0.010 mm, colour dark pink, habit block, temperature 100(2) Kelvin, λ(Synchrotron) 0.6889 Å, μ(Synchrotron) 1.586 mm^{-1}, T(Analytical)$_{min,max}$ 0.9945875330204204, 1.0, $2\theta_{max}$ 33.36, hkl range -23 23, -17 17, -30 30, N 40706, N_{ind} 12494(R_{merge} 0.0952), N_{obs} 4457(I > 2σ(I)), N_{var} 908, residuals* $R1(F)$ 0.1222, $wR2(F^2)$ 0.3215, GoF(all) 1.026, $\Delta\rho_{min,max}$ -1.433, 1.791 e$^-$ Å^{-3}.

*$R1 = \Sigma||F_o| - |F_c||/\Sigma|F_o|$ for $F_o > 2\sigma(F_o)$; $wR2 = (\Sigma w(F_o^2 - F_c^2)^2/\Sigma(wF_c^2)^2)^{1/2}$ all reflections

$w=1/[\sigma^2(F_o^2)+(0.1400P)^2]$ where $P=(F_o^2+2F_c^2)/3$

8. Volume calculations

In order to determine the available void space within the PM7-optimized molecular models of **1**, **2**, $(BF_4^-)_2\subset$**2'** and **3** and the X-ray structure of $(ReO_4^-)_2\subset$**2'**, VOIDOO calculations[13] were performed. In each case a virtual probe with the minimum size such that it would not exit the host cavity during calculations was selected. The standard parameters given below were used, following the previously published procedure[14] and the results are given in Table S1. The larger volume of the cavities in $(BF_4^-)_2\subset$**2'** and $(ReO_4^-)_2\subset$**2'** compared to **2** modelled without any bound anions can be attributed to guest induced expansion of the cavities.

Maximum number of volume-refinement cycles: 30

Minimum size of secondary grid: 3

Primary grid spacing: 0.1

Table S1. Calculated cavity volumes of the structures.

Complex	Probe radius (Å)	Plot grid spacing	Cavity volume(s) (Å^3)
**1**	9.0	0.7	15095.0
**2**	1.6	0.1	54.2, 55.2
$(BF_4^-)_2\subset$**2'**	1.6	0.1	69.8, 65.7
$(ReO_4^-)_2\subset$**2'**	1.9	0.1	75.9, 76.7
**3**	1.4	0.2	370.9

9. Molecular modelling

Geometry optimized structures were modelled at the PM7 level of theory[15] using the program MOPAC2016 (version 21.041W)[16] with the following parameters: RHF (restricted Hartree-Fock Hamiltonian), LBFGS (low memory Broyden-Fletcher-Goldfarb-Shanno procedure), a maximum of 2000 SCF (self-consistent field) iterations and a SCF criterion at 10^{-4} kcal/mol. The cartesian coordinates of these models are given in Tables S2-S4 below.

Table S2. Cartesian coordinates (in Å) for the PM7 model of pseudo-icosahedron **1** (all Δ handedness).

C	23.6811	40.3936	46.9716	H	24.5820	44.9194	46.6510	H	22.8837	53.4374	38.8852
C	24.1649	41.6122	47.4914	H	24.5407	38.1384	50.9133	H	20.9887	57.8079	39.4308
C	24.6823	41.6592	48.8230	H	23.5897	36.0565	50.0049	H	21.4692	57.4550	41.1446
C	25.1236	42.8870	49.3376	H	22.7733	35.9184	47.6847	H	19.9358	58.3578	40.7956
C	25.0650	44.0285	48.5459	H	22.2886	42.4128	43.0556	C	25.2444	52.8687	41.0575
C	24.5963	43.9990	47.2329	H	21.3888	40.2915	42.1989	C	24.5698	53.1652	42.2492
C	24.1250	42.7989	46.6923	H	21.6355	38.1930	43.4519	C	23.3582	52.5414	42.5357
C	23.7164	39.2252	47.7574	H	22.2706	45.1246	45.3742	C	22.8322	51.6300	41.6191
C	24.2231	39.2782	49.0947	H	22.5992	46.9425	43.7309	N	23.4620	51.3469	40.4316
C	24.1852	38.1225	49.8823	H	25.5151	44.5354	41.6510	C	24.6452	51.9597	40.1667
C	23.6544	36.9450	49.3587	H	25.2654	42.7632	43.3348	C	21.5528	50.9324	41.7880
C	23.1863	36.8609	48.0493	H	27.3228	40.1596	50.1930	N	21.1838	50.1372	40.8079
C	23.2104	37.9948	47.2300	H	28.3612	40.3809	52.4213	C	19.8880	49.5602	40.8240
C	23.1334	40.3509	45.6718	H	24.4819	41.0992	54.1649	C	18.7760	50.3045	41.2736
C	23.0553	41.5423	44.8904	H	23.4322	40.8903	51.9334	C	17.5018	49.7834	41.1868
C	22.4204	41.5054	43.6440	H	20.1949	37.0815	45.6163	C	17.3191	48.4848	40.6712
C	21.9121	40.3005	43.1635	H	19.4970	34.9907	44.4959	C	18.4403	47.7156	40.2584
C	22.0346	39.1141	43.8765	H	23.6362	33.9506	43.8369	C	19.7057	48.2505	40.3376
C	22.6307	39.1228	45.1470	H	24.3386	36.0655	44.8981	O	16.1620	47.8575	40.5143
N	24.7299	40.4874	49.5823	C	21.0990	47.0059	36.4344	C	14.9485	48.5229	40.9032
N	23.6036	42.7279	45.3958	C	20.0874	47.7065	35.7594	H	24.9905	53.8919	42.9572
N	22.7359	37.9630	45.9148	C	19.7463	48.9894	36.1681	H	22.8127	52.7775	43.4611
C	23.7638	43.8327	44.4728	C	20.4459	49.5600	37.2330	H	25.1205	51.7331	39.2021
C	23.0095	45.0040	44.5705	N	21.3970	48.8746	37.9495	H	20.9567	51.1243	42.6933
C	23.1875	46.0251	43.6297	C	21.7010	47.6094	37.5527	H	18.9172	51.3165	41.6708
C	24.0971	45.8875	42.5771	C	20.2629	50.9496	37.6550	H	16.6379	50.3743	41.5229
C	24.8262	44.6887	42.4817	N	21.1169	51.4097	38.5398	H	18.2708	46.7034	39.8919
C	24.6800	43.6783	43.4217	C	20.8557	52.7093	39.0709	H	20.5676	47.6551	40.0404
C	25.3177	40.5410	50.9110	C	19.5253	53.0284	39.4448	H	14.7833	49.4279	40.3075
C	26.7002	40.3737	51.0601	C	19.2199	54.2769	39.9223	H	14.9341	48.7237	41.9806
C	27.2833	40.4965	52.3212	C	20.2424	55.2611	40.0061	H	14.1911	47.7542	40.6475
C	26.5061	40.7917	53.4482	C	21.5650	54.9450	39.6486	C	24.3460	46.9281	41.5652
C	25.1182	40.9053	53.2909	C	21.8643	53.6712	39.1876	C	25.6554	47.0711	41.0543
C	24.5210	40.7836	52.0348	O	19.7885	56.4280	40.4461	C	25.9032	47.9026	39.9799
C	22.3161	36.7077	45.3203	C	20.6442	57.5717	40.4422	C	24.8398	48.6375	39.4323
C	20.9558	36.3997	45.2120	H	19.5750	47.2563	34.8961	N	23.5928	48.6350	40.0229
C	20.5694	35.2140	44.5900	H	18.9523	49.5539	35.6522	C	23.3543	47.7684	41.0501
C	21.5217	34.3113	44.0944	H	22.4531	47.0782	38.1460	C	24.8769	49.3854	38.1987
C	22.8784	34.6381	44.2081	H	19.4358	51.5393	37.2185	N	23.7452	50.0352	37.9060
C	23.2797	35.8281	44.8144	H	18.7373	52.2743	39.3519	C	23.5494	50.5275	36.5815
H	25.4917	42.9619	50.3611	H	18.1930	54.5551	40.2126	C	23.7003	49.6527	35.4883
H	25.3876	44.9886	48.9718	H	22.3421	55.7034	39.7053	C	23.3240	50.0622	34.2272

C	22.8143	51.3703	34.0391	H	55.4391	35.0855	46.6519	C	60.3027	31.7481	40.3315
C	22.7395	52.2696	35.1159	H	55.4759	41.8642	50.9180	O	63.8482	32.1292	40.5021
C	23.1153	51.8455	36.3786	H	56.4254	43.9471	50.0105	C	65.0597	31.4606	40.8920
O	22.4436	51.6150	32.7819	H	57.2376	44.0882	47.6887	H	55.0075	26.1184	42.9577
C	21.8244	52.8715	32.4803	H	57.7202	37.5983	43.0533	H	57.1862	27.2311	43.4612
H	26.4765	46.5149	41.5114	H	58.6166	39.7211	42.1965	H	54.8794	28.2757	39.2017
H	26.9000	47.9969	39.5534	H	58.3714	41.8181	43.4525	H	59.0447	28.8803	42.6917
H	22.3260	47.7640	41.4507	H	57.7469	34.8843	45.3683	H	61.0827	28.6822	41.6701
H	25.7408	49.3483	37.5437	H	57.4158	33.0675	43.7240	H	63.3645	29.6171	41.5196
H	24.0832	48.6464	35.6474	H	54.4912	35.4737	41.6551	H	61.7424	33.2891	39.8803
H	23.4012	49.3987	33.3658	H	54.7427	37.2434	43.3405	H	59.4423	32.3458	40.0349
H	22.3898	53.2956	34.9634	H	52.6924	39.8418	50.1931	H	65.2193	30.5510	40.3020
H	23.0738	52.5437	37.2202	H	51.6515	39.6175	52.4196	H	65.0757	31.2671	41.9707
H	20.8979	53.0062	33.0502	H	55.5292	38.9003	54.1673	H	65.8201	32.2241	40.6294
H	21.5939	52.7469	31.4042	H	56.5814	39.1122	51.9372	C	55.6631	33.0823	41.5631
H	22.5230	53.7009	32.6252	H	59.8129	42.9323	45.6186	C	54.3538	32.9430	41.0511
Fe	22.4113	50.0649	39.2550	H	60.5062	45.0237	44.4960	C	54.1043	32.1122	39.9766
C	56.3323	39.6129	46.9732	H	56.3644	46.0542	43.8369	C	55.1658	31.3740	39.4298
C	55.8507	38.3933	47.4927	H	55.6671	43.9383	44.8991	N	56.4121	31.3726	40.0215
C	55.3345	38.3450	48.8247	C	58.9063	33.0005	36.4355	C	56.6525	32.2385	41.0490
C	54.8958	37.1163	49.3392	C	59.9178	32.2999	35.7604	C	55.1271	30.6258	38.1964
C	54.9561	35.9749	48.5475	C	60.2570	31.0159	36.1673	N	56.2567	29.9723	37.9047
C	55.4234	36.0057	47.2339	C	59.5564	30.4445	37.2311	C	56.4516	29.4770	36.5812
C	55.8916	37.2070	46.6932	N	58.6059	31.1302	37.9482	C	56.3101	30.3517	35.4866
C	56.2968	40.7806	47.7601	C	58.3030	32.3960	37.5526	C	56.6860	29.9377	34.2269
C	55.7916	40.7263	49.0978	C	59.7371	29.0541	37.6512	C	57.1849	28.6250	34.0415
C	55.8300	41.8812	49.8866	N	58.8830	28.5945	38.5363	C	57.2500	27.7264	35.1194
C	56.3598	43.0593	49.3633	C	59.1428	27.2942	39.0664	C	56.8756	28.1554	36.3811
C	56.8258	43.1451	48.0531	C	60.4731	26.9731	39.4391	O	57.5560	28.3756	32.7854
C	56.8012	42.0118	47.2330	C	60.7773	25.7239	39.9156	C	58.1628	27.1125	32.4861
C	56.8781	39.6571	45.6726	C	59.7535	24.7412	40.0001	H	53.5335	33.5009	41.5076
C	56.9564	38.4663	44.8904	C	58.4308	25.0595	39.6446	H	53.1075	32.0211	39.5494
C	57.5887	38.5051	43.6426	C	58.1330	26.3339	39.1841	H	57.6804	32.2395	41.4505
C	58.0952	39.7108	43.1622	O	60.2059	23.5732	40.4388	H	54.2635	30.6650	37.5411
C	57.9733	40.8964	43.8768	C	59.3482	22.4311	40.4353	H	55.9345	31.3611	35.6438
C	57.3790	40.8861	45.1480	H	60.4313	32.7510	34.8982	H	56.6164	30.6007	33.3643
N	55.2860	39.5163	49.5848	H	61.0501	30.4508	35.6506	H	57.5914	26.6974	34.9690
N	56.4110	37.2796	45.3959	H	57.5506	32.9270	38.1458	H	56.9108	27.4583	37.2238
N	57.2738	42.0452	45.9171	H	60.5625	28.4632	37.2132	H	59.0858	26.9679	33.0592
C	56.2500	36.1755	44.4723	H	61.2621	27.7261	39.3460	H	58.3983	27.2343	31.4108
C	57.0059	35.0049	44.5666	H	61.8040	25.4439	40.2046	H	57.4548	26.2908	32.6287
C	56.8265	33.9846	43.6252	H	57.6527	24.3023	39.7021	Fe	57.5910	29.9405	39.2533
C	55.9139	34.1222	42.5752	H	57.1134	26.5692	38.8832	C	56.3319	40.3913	33.0249
C	55.1829	35.3201	42.4834	H	59.0049	22.1945	39.4236	C	55.8487	41.6100	32.5049
C	55.3303	36.3295	43.4243	H	58.5222	22.5501	41.1361	C	55.3316	41.6574	31.1733
C	54.6970	39.4614	50.9128	H	60.0545	21.6441	40.7906	C	54.8908	42.8852	30.6587
C	53.3142	39.6275	51.0606	C	54.7545	27.1408	41.0575	C	54.9503	44.0270	31.4500
C	52.7297	39.5030	52.3208	C	55.4288	26.8442	42.2494	C	55.4193	43.9974	32.7630
C	53.5056	39.2072	53.4486	C	56.6411	27.4670	42.5356	C	55.8891	42.7968	33.3038
C	54.8938	39.0947	53.2928	C	57.1684	28.3769	41.6181	C	56.2968	39.2232	32.2387
C	55.4923	39.2181	52.0374	N	56.5388	28.6600	40.4304	C	55.7909	39.2765	30.9013
C	57.6910	43.3013	45.3223	C	55.3548	28.0487	40.1661	C	55.8292	38.1212	30.1132
C	59.0506	43.6124	45.2138	C	58.4488	29.0726	41.7864	C	56.3595	36.9433	30.6367
C	59.4343	44.7983	44.5907	N	58.8193	29.8662	40.8054	C	56.8269	36.8587	31.9465
C	58.4799	45.6985	44.0944	C	60.1166	30.4398	40.8205	C	56.8024	37.9925	32.7660
C	57.1240	45.3688	44.2086	C	61.2267	29.6930	41.2710	C	56.8788	40.3482	34.3251
C	56.7254	44.1782	44.8156	C	62.5024	30.2100	41.1823	C	56.9564	41.5395	35.1068
H	54.5286	37.0404	50.3629	C	62.6891	31.5065	40.6627	C	57.5888	41.5018	36.3545
H	54.6361	35.0140	48.9734	C	61.5699	32.2783	40.2494	C	58.0956	40.2964	36.8354

C	57.9741	39.1104	36.1216	C	59.3497	57.5641	39.5280	H	55.9283	48.6444	44.3481
C	57.3805	39.1197	34.8501	H	60.4212	47.2575	45.1016	H	56.6127	49.4016	46.6275
N	55.2841	40.4857	30.4138	H	61.0424	49.5562	44.3468	H	57.5956	53.3041	45.0252
N	56.4099	42.7254	34.6006	H	57.5486	47.0767	41.8469	H	56.9128	52.5458	42.7700
N	57.2764	37.9603	34.0815	H	60.5604	51.5407	42.7790	H	59.0889	53.0313	46.9309
C	56.2477	43.8300	35.5234	H	61.2608	52.2733	40.6435	H	58.4083	52.7611	48.5815
C	57.0068	44.9987	35.4315	H	61.8039	54.5515	39.7743	H	57.4601	53.7083	47.3697
C	56.8258	46.0198	36.3717	H	57.6550	55.7002	40.2808	Fe	57.5879	50.0642	40.7395
C	55.9082	45.8848	37.4177	H	57.1144	53.4372	41.1092	C	23.6782	39.6159	33.0260
C	55.1737	44.6888	37.5068	H	59.0089	57.8086	40.5388	C	24.1619	38.3967	32.5077
C	55.3230	43.6785	36.5673	H	58.5221	57.4405	38.8299	C	24.6804	38.3484	31.1766
C	54.6952	40.5395	29.0858	H	60.0557	58.3481	39.1653	C	25.1208	37.1200	30.6630
C	53.3121	40.3757	28.9386	C	54.7547	52.8708	38.9401	C	25.0601	35.9787	31.4548
C	52.7276	40.4989	27.6783	C	55.4301	53.1694	37.7493	C	24.5899	36.0093	32.7674
C	53.5039	40.7912	26.5499	C	56.6414	52.5454	37.4620	C	24.1198	37.2103	33.3071
C	54.8923	40.9017	26.7052	C	57.1668	51.6320	38.3771	C	23.7145	40.7835	32.2390
C	55.4909	40.7793	27.9607	N	56.5363	51.3467	39.5637	C	24.2224	40.7293	30.9023
C	57.6947	36.7046	34.6760	C	55.3530	51.9591	39.8290	C	24.1852	41.8841	30.1134
C	59.0547	36.3969	34.7889	C	58.4460	50.9343	38.2074	C	23.6535	43.0620	30.6354
C	59.4393	35.2107	35.4109	N	58.8155	50.1383	39.1867	C	23.1838	43.1474	31.9442
C	58.4854	34.3073	35.9022	C	60.1114	49.5617	39.1696	C	23.2073	42.0142	32.7645
C	57.1291	34.6336	35.7838	C	61.2227	50.3063	38.7183	C	23.1280	39.6596	34.3247
C	56.7295	35.8239	35.1772	C	62.4972	49.7857	38.8041	C	23.0482	38.4686	35.1066
H	54.5229	42.9603	29.6352	C	62.6811	48.4877	39.3206	C	22.4108	38.5063	36.3518
H	54.6284	44.9872	31.0240	C	61.5606	47.7183	39.7350	C	21.9012	39.7115	36.8305
H	55.4351	44.9180	33.3446	C	60.2948	48.2523	39.6566	C	22.0252	40.8973	36.1168
H	55.4745	38.1374	29.0819	O	63.8387	47.8611	39.4773	C	22.6240	40.8880	34.8476
H	56.4245	36.0552	29.9902	C	65.0513	48.5264	39.0855	N	24.7290	39.5194	30.4161
H	57.2396	35.9160	32.3109	H	55.0102	53.8975	37.0425	N	23.5966	37.2824	34.6028
H	57.7197	42.4090	36.9434	H	57.1870	52.7826	36.5369	N	22.7305	42.0471	34.0789
H	58.6170	40.2868	37.8011	H	54.8765	51.7299	40.7924	C	23.7573	36.1789	35.5272
H	58.3721	38.1890	36.5467	H	59.0415	51.1266	37.3017	C	23.0025	35.0077	35.4324
H	57.7509	45.1173	34.6323	H	61.0808	51.3180	38.3205	C	23.1817	33.9879	36.3743
H	57.4179	46.9354	36.2750	H	63.3605	50.3768	38.4665	C	24.0925	34.1270	37.4258
H	54.4777	44.5374	38.3319	H	61.7311	46.7064	40.1019	C	24.8220	35.3258	37.5184
H	54.7333	42.7658	36.6492	H	59.4335	47.6566	39.9547	C	24.6751	36.3345	36.5768
H	52.6900	40.1639	29.8065	H	65.2161	49.4339	39.6775	C	25.3178	39.4643	29.0880
H	51.6492	40.3860	27.5798	H	65.0650	48.7227	38.0072	C	26.7006	39.6299	28.9400
H	55.5276	41.0935	25.8301	H	65.8097	47.7594	39.3439	C	27.2848	39.5055	27.6796
H	56.5801	40.8836	28.0605	C	55.6570	46.9255	38.4289	C	26.5086	39.2102	26.5520
H	59.8167	37.0796	34.3880	C	54.3472	47.0681	38.9387	C	25.1204	39.0982	26.7081
H	60.5114	34.9874	35.5085	C	54.0982	47.8995	40.0130	C	24.5220	39.2218	27.9635
H	56.3700	33.9453	36.1513	C	55.1608	48.6347	40.5617	C	22.3119	43.3030	34.6728
H	55.6709	36.0609	35.0897	N	56.4078	48.6335	39.9714	C	20.9519	43.6122	34.7817
C	58.9000	47.0054	43.5607	C	56.6476	47.7671	38.9444	C	20.5668	44.7979	35.4046
C	59.9097	47.7073	44.2375	C	55.1228	49.3827	41.7953	C	21.5203	45.6995	35.9002
C	60.2500	48.9906	43.8294	N	56.2537	50.0336	42.0881	C	22.8766	45.3719	35.7851
C	59.5517	49.5605	42.7633	C	56.4492	50.5279	43.4118	C	23.2765	44.1818	35.1783
N	58.6025	48.8742	42.0454	C	56.3059	49.6528	44.5058	H	25.4894	37.0442	29.6398
C	58.2992	47.6084	42.4415	C	56.6833	50.0654	45.7656	H	25.3816	35.0180	31.0296
C	59.7342	50.9503	42.3417	C	57.1854	51.3767	45.9517	H	24.5733	35.0890	33.3493
N	58.8810	51.4094	41.4557	C	57.2519	52.2758	44.8743	H	24.5415	41.8673	29.0827
C	59.1422	52.7084	40.9232	C	56.8763	51.8485	43.6126	H	23.5891	43.9497	29.9879
C	60.4725	53.0268	40.5481	O	57.5587	51.6242	47.2077	H	22.7697	44.0901	32.3073
C	60.7773	54.2736	40.0658	C	58.1683	52.8859	47.5075	H	22.2777	37.5991	36.9401
C	59.7542	55.2568	39.9782	H	53.5263	46.5124	38.4808	H	21.3753	39.7210	37.7937
C	58.4321	54.9420	40.3387	H	53.1008	47.9934	40.4384	H	21.6247	41.8186	36.5399
C	58.1336	53.6698	40.8047	H	57.6760	47.7640	38.5440	H	22.2628	34.8861	34.6297
O	60.2068	56.4215	39.5308	H	54.2586	49.3450	42.4500	H	22.5935	33.0702	36.2749

H	25.5120	35.4805	38.3480	H	18.2581	33.2936	40.1098	C	42.4813	24.8261	44.6886
H	25.2614	37.2492	36.6611	H	20.5584	32.3502	39.9588	C	43.4213	24.6801	43.6783
H	27.3228	39.8440	29.8074	H	14.7829	30.5542	39.6769	C	50.9107	25.3185	40.5412
H	28.3630	39.6199	27.5805	H	14.9277	31.2761	38.0108	C	51.0598	26.7009	40.3741
H	24.4850	38.9043	25.8336	H	14.1816	32.2281	39.3548	C	52.3209	27.2840	40.4969
H	23.4330	39.1165	28.0638	C	24.3419	33.0875	38.4387	C	53.4480	26.5068	40.7918
H	20.1903	42.9312	34.3774	C	25.6495	32.9498	38.9554	C	53.2906	25.1189	40.9052
H	19.4947	45.0219	35.4996	C	25.8965	32.1180	40.0299	C	52.0345	24.5217	40.7835
H	23.6354	46.0588	36.1558	C	24.8341	31.3779	40.5722	C	45.3203	22.3165	36.7078
H	24.3351	43.9434	35.0939	N	23.5896	31.3761	39.9768	C	45.2120	20.9562	36.3997
C	21.0995	33.0048	43.5616	C	23.3517	32.2424	38.9492	C	44.5901	20.5698	35.2141
C	20.0895	32.3041	44.2392	C	24.8702	30.6271	41.8043	C	44.0943	21.5220	34.3114
C	19.7485	31.0207	43.8322	N	23.7404	29.9727	42.0921	C	44.2079	22.8787	34.6382
C	20.4455	30.4500	42.7656	C	23.5438	29.4707	43.4128	C	44.8143	23.2800	35.8281
N	21.3944	31.1356	42.0464	C	23.6838	30.3395	44.5123	H	50.3608	25.4923	42.9621
C	21.6989	32.4011	42.4420	C	23.3058	29.9185	45.7691	H	48.9714	25.3882	44.9888
C	20.2613	29.0607	42.3437	C	22.8062	28.6050	45.9466	H	46.6507	24.5823	44.9195
N	21.1124	28.6006	41.4559	C	22.7431	27.7123	44.8638	H	50.9131	24.5415	38.1386
C	20.8464	27.3027	40.9232	C	23.1196	28.1479	43.6051	H	50.0049	23.5905	36.0567
C	19.5136	26.9876	40.5540	O	22.4322	28.3490	47.2005	H	47.6847	22.7740	35.9184
C	19.2026	25.7405	40.0769	C	21.8222	27.0855	47.4914	H	43.0555	22.2887	42.4128
C	20.2218	24.7533	39.9883	H	26.4705	33.5096	38.5025	H	42.1989	21.3890	40.2915
C	21.5471	25.0659	40.3391	H	26.8920	32.0277	40.4605	H	43.4519	21.6358	38.1930
C	21.8518	26.3384	40.8002	H	22.3250	32.2427	38.5447	H	45.3741	22.2710	45.1246
O	19.7624	23.5875	39.5514	H	25.7326	30.6649	42.4614	H	43.7306	22.5993	46.9425
C	20.6156	22.4419	39.5482	H	24.0595	31.3497	44.3612	H	41.6504	25.5149	44.5354
H	19.5785	32.7548	45.1030	H	23.3739	30.5770	46.6354	H	43.3345	25.2655	42.7632
H	18.9563	30.4557	44.3503	H	22.4013	26.6825	45.0080	H	50.1927	27.3236	40.1602
H	22.4492	32.9325	41.8466	H	23.0858	27.4556	42.7585	H	52.4210	28.3620	40.3815
H	19.4349	28.4713	42.7818	H	20.9003	26.9457	46.9154	H	54.1646	24.4826	41.0989
H	18.7281	27.7439	40.6502	H	21.5843	27.2022	48.5668	H	51.9332	23.4328	40.8900
H	18.1738	25.4653	39.7906	H	22.5289	26.2631	47.3460	H	45.6164	20.1953	37.0816
H	22.3218	24.3054	40.2774	Fe	22.4079	29.9445	40.7410	H	44.4960	19.4974	34.9908
H	22.8734	26.5690	41.0979	C	46.9714	23.6816	40.3937	H	43.8366	23.6365	33.9507
H	20.9708	22.2061	40.5559	C	47.4912	24.1654	41.6123	H	44.8978	24.3389	36.0656
H	21.4330	22.5562	38.8366	C	48.8227	24.6829	41.6594	C	36.4341	21.0982	47.0060
H	19.9016	21.6570	39.2035	C	49.3373	25.1242	42.8871	C	35.7591	20.0865	47.7067
C	25.2466	27.1436	38.9421	C	48.5456	25.0655	44.0287	C	36.1680	19.7455	48.9896
C	24.5754	26.8490	37.7480	C	47.2326	24.5967	43.9992	C	37.2329	20.4452	49.5602
C	23.3640	27.4722	37.4595	C	46.6921	24.1254	42.7990	N	37.9493	21.3963	48.8747
C	22.8346	28.3816	38.3764	C	47.7573	23.7169	39.2254	C	37.5525	21.7003	47.6095
N	23.4615	28.6635	39.5658	C	49.0944	24.2237	39.2783	C	37.6550	20.2622	50.9497
C	24.6445	28.0510	39.8326	C	49.8822	24.1859	38.1227	N	38.5398	21.1164	51.4097
C	21.5549	29.0778	38.2060	C	49.3586	23.6551	36.9452	C	39.0710	20.8552	52.7093
N	21.1828	29.8715	39.1863	C	48.0492	23.1869	36.8610	C	39.4449	19.5248	53.0284
C	19.8857	30.4452	39.1690	C	47.2299	23.2110	37.9949	C	39.9225	19.2193	54.2768
C	18.7765	29.6991	38.7154	C	45.6717	23.1338	40.3510	C	40.0063	20.2418	55.2611
C	17.5006	30.2159	38.8025	C	44.8903	23.0556	41.5424	C	39.6489	21.5645	54.9450
C	17.3130	31.5117	39.3235	C	43.6439	22.4206	41.5054	C	39.1878	21.8637	53.6712
C	18.4314	32.2832	39.7398	C	43.1635	21.9123	40.3005	O	40.4464	19.7878	56.4280
C	19.6986	31.7529	39.6596	C	43.8765	22.0349	39.1141	C	40.4426	20.6435	57.5717
O	16.1535	32.1339	39.4831	C	45.1469	22.6311	39.1228	H	34.8959	19.5741	47.2565
C	14.9428	31.4659	39.0902	N	49.5820	24.7306	40.4876	H	35.6522	18.9516	49.5543
H	24.9983	26.1240	37.0397	N	45.3956	23.6040	42.7280	H	38.1457	22.4524	47.0782
H	22.8213	27.2376	36.5322	N	45.9147	22.7364	37.9630	H	37.2185	19.4352	51.5395
H	25.1175	28.2768	40.7985	C	44.4726	23.7641	43.8328	H	39.3520	18.7367	52.2744
H	20.9607	28.8860	37.2994	C	44.5703	23.0097	45.0041	H	40.2127	18.1924	54.5551
H	18.9210	28.6888	38.3152	C	43.6294	23.1876	46.0251	H	39.7057	22.3415	55.7035
H	16.6390	29.6238	38.4628	C	42.5767	24.0970	45.8875	H	38.8853	22.8832	53.4374

H	39.4312	20.9882	57.8079	C	47.4924	55.8497	38.3930	H	44.8986	55.6665	43.9379
H	41.1451	21.4684	57.4548	C	48.8244	55.3334	38.3446	C	36.4357	58.9063	32.9998
H	40.7958	19.9351	58.3577	C	49.3388	54.8947	37.1159	C	35.7606	59.9179	32.2992
C	41.0572	25.2441	52.8686	C	48.5472	54.9550	35.9746	C	36.1676	60.2570	31.0151
C	42.2490	24.5696	53.1650	C	47.2336	55.4224	36.0054	C	37.2313	59.5563	30.4438
C	42.5356	23.3580	52.5413	C	46.6929	55.8907	37.2066	N	37.9483	58.6058	31.1295
C	41.6190	22.8319	51.6299	C	47.7598	56.2958	40.7802	C	37.5528	58.3030	32.3953
N	40.4314	23.4616	51.3468	C	49.0974	55.7904	40.7259	C	37.6515	59.7371	29.0533
C	40.1664	24.6448	51.9596	C	49.8862	55.8287	41.8809	N	38.5365	58.8829	28.5938
C	41.7879	21.5526	50.9322	C	49.3628	56.3585	43.0590	C	39.0667	59.1427	27.2935
N	40.8078	21.1835	50.1371	C	48.0527	56.8247	43.1447	C	39.4394	60.4730	26.9723
C	40.8240	19.8877	49.5600	C	47.2327	56.8002	42.0114	C	39.9160	60.7772	25.7232
C	41.2736	18.7756	50.3043	C	45.6724	56.8773	39.6567	C	40.0005	59.7534	24.7405
C	41.1869	17.5015	49.7831	C	44.8902	56.9557	38.4659	C	39.6450	58.4307	25.0589
C	40.6712	17.3188	48.4846	C	43.6424	57.5881	38.5046	C	39.1845	58.1329	26.3332
C	40.2583	18.4400	47.7154	C	43.1621	58.0948	39.7103	O	40.4393	60.2058	23.5725
C	40.3375	19.7054	48.2504	C	43.8767	57.9727	40.8959	C	40.4359	59.3481	22.4305
O	40.5143	16.1617	47.8573	C	45.1478	57.3783	40.8857	H	34.8985	60.4314	32.7502
C	40.9032	14.9482	48.5226	N	49.5844	55.2848	39.5159	H	35.6508	61.0501	30.4501
H	42.9571	24.9903	53.8917	N	45.3956	56.4102	37.2791	H	38.1458	57.5506	32.9264
H	43.4610	22.8125	52.7773	N	45.9168	57.2730	42.0447	H	37.2135	60.5625	28.4625
H	39.2018	25.1200	51.7329	C	44.4721	56.2493	36.1750	H	39.3462	61.2620	27.7254
H	42.6932	20.9565	51.1241	C	44.5665	57.0052	35.0044	H	40.2050	61.8040	25.4432
H	41.6709	18.9168	51.3163	C	43.6252	56.8259	33.9841	H	39.7025	57.6525	24.3017
H	41.5231	16.6376	50.3740	C	42.5750	55.9135	34.1217	H	38.8835	57.1134	26.5685
H	39.8919	18.2704	46.7032	C	42.4831	55.1824	35.3196	H	39.4242	59.0046	22.1938
H	40.0403	20.5673	47.6550	C	43.4239	55.3297	36.3290	H	41.1367	58.5222	22.5495
H	40.3075	14.7828	49.4275	C	50.9126	54.6961	39.4610	H	40.7912	60.0544	21.6435
H	41.9807	14.9337	48.7234	C	51.0608	53.3133	39.6264	C	41.0576	54.7542	27.1403
H	40.6476	14.1907	47.7539	C	52.3212	52.7293	39.5021	C	42.2495	55.4285	26.8437
C	41.5647	24.3458	46.9280	C	53.4489	53.5057	39.2071	C	42.5357	56.6408	27.4664
C	41.0535	25.6550	47.0709	C	53.2926	54.8939	39.0951	C	41.6182	57.1681	28.3763
C	39.9791	25.9027	47.9024	C	52.0370	55.4920	39.2184	N	40.4305	56.5387	28.6593
C	39.4317	24.8392	48.6374	C	45.3220	57.6902	43.3008	C	40.1662	55.3546	28.0481
N	40.0225	23.5924	48.6350	C	45.2137	59.0499	43.6119	C	41.7865	58.4486	29.0720
C	41.0497	23.3540	47.7683	C	44.5906	59.4337	44.7978	N	40.8056	58.8191	29.8656
C	38.1981	24.8762	49.3854	C	44.0943	58.4794	45.6980	C	40.8208	60.1164	30.4392
N	37.9056	23.7446	50.0353	C	44.2083	57.1235	45.3683	C	41.2713	61.2264	29.6923
C	36.5813	23.5487	50.5279	C	44.8152	56.7248	44.1778	C	41.1827	62.5022	30.2094
C	35.4879	23.6994	49.6532	H	50.3626	54.5274	37.0401	C	40.6631	62.6889	31.5058
C	34.2269	23.3231	50.0631	H	48.9730	54.6349	35.0136	C	40.2497	61.5697	32.2777
C	34.0391	22.8135	51.3713	H	46.6516	55.4381	35.0851	C	40.3317	60.3026	31.7475
C	35.1160	22.7389	52.2703	H	50.9175	55.4745	41.8640	O	40.5025	63.8480	32.1285
C	36.3787	23.1147	51.8460	H	50.0100	56.4239	43.9468	C	40.8925	65.0594	31.4598
O	32.7820	22.4429	51.6163	H	47.6883	57.2365	44.0878	H	42.9578	55.0071	26.1180
C	32.4806	21.8238	52.8730	H	43.0532	57.7197	37.5977	H	43.4615	57.1857	27.2306
H	41.5104	26.4763	46.5148	H	42.1965	58.6162	39.7205	H	39.2017	54.8792	28.2750
H	39.5523	26.8994	47.9966	H	43.4524	58.3710	41.8176	H	42.6920	59.0444	28.8797
H	41.4505	22.3258	47.7640	H	45.3684	57.7461	34.8838	H	41.6704	61.0823	28.6815
H	37.5429	25.7400	49.3482	H	43.7241	57.4152	33.0670	H	41.5201	63.3642	29.6164
H	35.6468	24.0821	48.6468	H	41.6546	54.4909	35.4731	H	39.8806	61.7423	33.2885
H	33.3653	23.4001	49.3998	H	43.3400	54.7422	37.2430	H	40.0351	59.4422	32.3452
H	34.9638	22.3892	53.2964	H	50.1934	52.6911	39.8401	H	40.3026	65.2191	30.5502
H	37.2204	23.0733	52.5440	H	52.4203	51.6510	39.6161	H	41.9712	65.0754	31.2662
H	33.0505	20.8974	53.0077	H	54.1670	55.5296	38.9013	H	40.6299	65.8198	32.2234
H	31.4045	21.5934	52.7486	H	51.9366	56.5810	39.1128	C	41.5629	55.6628	33.0817
H	32.6257	22.5226	53.7022	H	45.6185	59.8121	42.9318	C	41.0508	54.3535	32.9424
Fe	39.2547	22.4108	50.0649	H	44.4961	60.5056	45.0232	C	39.9763	54.1042	32.1117
C	46.9729	56.3314	39.6125	H	43.8365	56.3639	46.0538	C	39.4296	55.1656	31.3733

N	40.0215	56.4119	31.3719	C	34.7891	59.0549	36.3962	C	38.2073	58.4459	50.9339
C	41.0489	56.6522	32.2379	C	35.4111	59.4395	35.2099	N	39.1866	58.8154	50.1379
C	38.1963	55.1271	30.6250	C	35.9024	58.4856	34.3066	C	39.1696	60.1113	49.5613
N	37.9047	56.2567	29.9715	C	35.7838	57.1292	34.6329	C	38.7183	61.2227	50.3058
C	36.5813	56.4516	29.4761	C	35.1772	56.7297	35.8232	C	38.8041	62.4972	49.7853
C	35.4866	56.3103	30.3508	H	29.6353	54.5232	42.9597	C	39.3207	62.6811	48.4873
C	34.2270	56.6864	29.9366	H	31.0241	54.6286	44.9866	C	39.7350	61.5606	47.7178
C	34.0418	57.1851	28.6239	H	33.3448	55.4353	44.9173	C	39.6566	60.2947	48.2519
C	35.1197	57.2501	27.7254	H	29.0820	55.4749	38.1368	O	39.4774	63.8386	47.8606
C	36.3812	56.8756	28.1544	H	29.9902	56.4249	36.0546	C	39.0856	65.0513	48.5260
O	32.7856	57.5564	28.3744	H	32.3110	57.2400	35.9154	H	37.0423	55.0101	53.8971
C	32.4865	58.1631	27.1111	H	36.9436	57.7199	42.4083	H	36.5369	57.1869	52.7823
H	41.5071	53.5332	33.5004	H	37.8012	58.6172	40.2861	H	40.7922	54.8763	51.7296
H	39.5490	53.1073	32.0205	H	36.5468	58.3723	38.1882	H	37.3016	59.0414	51.1262
H	41.4505	57.6801	32.2388	H	34.6325	57.7510	45.1167	H	38.3206	61.0807	51.3174
H	37.5409	54.2636	30.6642	H	36.2750	57.4178	46.9348	H	38.4665	63.3603	50.3765
H	35.6437	55.9349	31.3602	H	38.3321	54.4778	44.5368	H	40.1019	61.7310	46.7059
H	33.3643	56.6169	30.5996	H	36.6493	54.7335	42.7651	H	39.9546	59.4334	47.6563
H	34.9694	57.5915	26.6963	H	29.8066	52.6904	40.1629	H	39.6776	65.2159	49.4335
H	37.2240	56.9106	27.4574	H	27.5800	51.6495	40.3853	H	38.0074	65.0649	48.7224
H	33.0598	59.0860	26.9665	H	25.8302	55.5277	41.0937	H	39.3439	65.8097	47.7590
H	31.4112	58.3986	27.2329	H	28.0605	56.5804	40.8834	C	38.4290	55.6570	46.9249
H	32.6292	57.4550	26.2896	H	34.3882	59.8169	37.0788	C	38.9388	54.3472	47.0674
Fe	39.2535	57.5908	29.9398	H	35.5087	60.5116	34.9867	C	40.0130	54.0981	47.8989
C	33.0250	56.3322	40.3906	H	36.1513	56.3702	33.9446	C	40.5616	55.1607	48.6343
C	32.5050	55.8490	41.6093	H	35.0897	55.6711	36.0603	N	39.9713	56.4077	48.6331
C	31.1734	55.3320	41.6568	C	43.5606	58.8996	47.0049	C	38.9443	56.6475	47.7666
C	30.6588	54.8912	42.8846	C	44.2375	59.9093	47.7068	C	41.7952	55.1226	49.3822
C	31.4502	54.9506	44.0264	C	43.8295	60.2497	48.9901	N	42.0879	56.2535	50.0332
C	32.7632	55.4196	43.9968	C	42.7633	59.5514	49.5599	C	43.4116	56.4490	50.5277
C	33.3040	55.8894	42.7961	N	42.0453	58.6022	48.8737	C	44.5057	56.3057	49.6526
C	32.2388	56.2972	39.2226	C	42.4413	58.2989	47.6080	C	45.7655	56.6831	50.0653
C	30.9013	55.7913	39.2759	C	42.3417	59.7340	50.9498	C	45.9514	57.1852	51.3767
C	30.1132	55.8296	38.1206	N	41.4556	58.8809	51.4090	C	44.8739	57.2516	52.2757
C	30.6368	56.3599	36.9427	C	40.9231	59.1423	52.7080	C	43.6123	56.8761	51.8483
C	31.9465	56.8273	36.8581	C	40.5481	60.4726	53.0262	O	47.2073	57.5584	51.6243
C	32.7660	56.8028	37.9919	C	40.0658	60.7776	54.2730	C	47.5071	58.1679	52.8861
C	34.3252	56.8790	40.3475	C	39.9781	59.7545	55.2562	H	38.4810	53.5263	46.5116
C	35.1069	56.9566	41.5388	C	40.3385	58.4324	54.9416	H	40.4384	53.1007	47.9927
C	36.3546	57.5890	41.5011	C	40.8044	58.1337	53.6694	H	38.5439	57.6759	47.7636
C	36.8356	58.0958	40.2957	O	39.5307	60.2073	56.4209	H	42.4499	54.2585	49.3446
C	36.1217	57.9744	39.1097	C	39.5278	59.3503	57.5636	H	44.3481	55.9281	48.6442
C	34.8502	57.3807	39.1190	H	45.1015	60.4207	47.2570	H	46.6275	56.6125	49.4017
N	30.4139	55.2845	40.4851	H	44.3469	61.0420	49.5556	H	45.0249	57.5953	53.3039
N	34.6007	56.4101	42.7247	H	41.8467	57.5483	47.0763	H	42.7697	56.9126	52.5455
N	34.0816	57.2766	37.9596	H	42.7790	60.5602	51.5401	H	46.9305	59.0886	53.0315
C	35.5235	56.2478	43.8293	H	40.6437	61.2609	52.2726	H	48.5811	58.4079	52.7613
C	35.4316	57.0069	44.9981	H	39.7745	61.8043	54.5508	H	47.3694	57.4597	53.7084
C	36.3718	56.8258	46.0192	H	40.2804	57.6553	55.6999	Fe	40.7394	57.5877	50.0638
C	37.4178	55.9082	45.8842	H	41.1088	57.1145	53.4369	C	33.0255	23.6769	39.6161
C	37.5070	55.1738	44.6881	H	40.5385	59.0093	57.8080	C	32.5071	24.1607	38.3969
C	36.5675	55.3232	43.6778	H	38.8296	58.5228	57.4400	C	31.1760	24.6791	38.3486
C	29.0858	54.6956	40.5390	H	39.1652	60.0564	58.3474	C	30.6625	25.1195	37.1202
C	28.9387	53.3125	40.3749	C	38.9400	54.7546	52.8704	C	31.4543	25.0589	35.9789
C	27.6785	52.7279	40.4983	C	37.7492	55.4300	53.1690	C	32.7669	24.5888	36.0095
C	26.5501	53.5041	40.7910	C	37.4619	56.6413	52.5450	C	33.3065	24.1187	37.2105
C	26.7053	54.8924	40.9016	C	38.3770	57.1667	51.6316	C	32.2384	23.7132	40.7836
C	27.9607	55.4911	40.7791	N	39.5636	56.5362	51.3463	C	30.9017	24.2209	40.7295
C	34.6761	57.6949	36.7039	C	39.8289	55.3529	51.9587	C	30.1127	24.1836	41.8843

C	30.6348	23.6519	43.0622	C	40.9230	20.8466	27.3029	C	43.6048	23.1200	28.1482
C	31.9437	23.1822	43.1476	C	40.5539	19.5138	26.9877	O	47.2002	22.4330	28.3493
C	32.7640	23.2060	42.0144	C	40.0767	19.2028	25.7406	C	47.4913	21.8232	27.0858
C	34.3243	23.1269	39.6598	C	39.9880	20.2220	24.7534	H	38.5018	26.4701	33.5099
C	35.1062	23.0473	38.4688	C	40.3388	21.5473	25.0660	H	40.4596	26.8920	32.0281
C	36.3514	22.4099	38.5065	C	40.7999	21.8520	26.3385	H	38.5444	22.3247	32.2430
C	36.8301	21.9003	39.7116	O	39.5511	19.7625	23.5876	H	42.4608	25.7329	30.6653
C	36.1164	22.0242	40.8975	C	39.5478	20.6157	22.4420	H	44.3607	24.0601	31.3500
C	34.8472	22.6228	40.8882	H	45.1030	19.5790	32.7549	H	46.6350	23.3747	30.5773
N	30.4155	24.7276	39.5196	H	44.3503	18.9567	30.4557	H	45.0079	22.4018	26.6828
N	34.6024	23.5957	37.2827	H	41.8465	22.4493	32.9326	H	42.7582	23.0861	27.4558
N	34.0784	22.7293	42.0473	H	42.7816	19.4351	28.4714	H	46.9154	20.9012	26.9460
C	35.5268	23.7565	36.1792	H	40.6502	18.7282	27.7439	H	48.5667	21.5854	27.2025
C	35.4320	23.0018	35.0079	H	39.7905	18.1740	25.4655	H	47.3458	22.5299	26.2635
C	36.3740	23.1811	33.9882	H	40.2770	22.3222	24.3057	Fe	40.7407	22.4080	29.9447
C	37.4254	24.0920	34.1273	H	41.0976	22.8736	26.5693	C	40.3937	46.9711	23.6811
C	37.5179	24.8215	35.3261	H	40.5556	20.9709	22.2063	C	41.6123	47.4909	24.1649
C	36.5762	24.6744	36.3348	H	38.8362	21.4331	22.5563	C	41.6594	48.8225	24.6824
C	29.0875	25.3167	39.4645	H	39.2032	19.9019	21.6570	C	42.8872	49.3370	25.1237
C	28.9398	26.6994	39.6307	C	38.9416	25.2466	27.1439	C	44.0287	48.5453	25.0650
C	27.6796	27.2839	39.5064	C	37.7476	24.5754	26.8493	C	43.9992	47.2323	24.5962
C	26.5519	26.5082	39.2104	C	37.4592	23.3639	27.4725	C	42.7990	46.6918	24.1249
C	26.7076	25.1199	39.0980	C	38.3761	22.8346	28.3818	C	39.2253	47.7570	23.7165
C	27.9629	24.5213	39.2215	N	39.5655	23.4615	28.6637	C	39.2783	49.0942	24.2232
C	34.6723	22.3107	43.3032	C	39.8323	24.6445	28.0512	C	38.1226	49.8819	24.1855
C	34.7814	20.9508	43.6124	C	38.2057	21.5548	29.0780	C	36.9451	49.3583	23.6547
C	35.4043	20.5658	44.7981	N	39.1860	21.1827	29.8717	C	36.8610	48.0489	23.1864
C	35.8998	21.5194	45.6997	C	39.1690	19.8855	30.4453	C	37.9949	47.2296	23.2105
C	35.7845	22.8756	45.3720	C	38.7154	18.7763	29.6992	C	40.3509	45.6713	23.1334
C	35.1777	23.2755	44.1819	C	38.8026	17.5004	30.2160	C	41.5423	44.8900	23.0552
H	29.6392	25.4881	37.0444	C	39.3237	17.3128	31.5117	C	41.5054	43.6435	22.4203
H	31.0290	25.3804	35.0182	C	39.7399	18.4312	32.2833	C	40.3005	43.1631	21.9120
H	33.3488	24.5723	35.0892	C	39.6595	19.6985	31.7529	C	39.1141	43.8761	22.0345
H	29.0820	24.5398	41.8675	O	39.4834	16.1532	32.1338	C	39.1228	45.1466	22.6307
H	29.9873	23.5873	43.9497	C	39.0906	14.9426	31.4657	N	40.4876	49.5817	24.7301
H	32.3068	22.7681	44.0901	H	37.0393	24.9983	26.1243	N	42.7279	45.3953	23.6035
H	36.9397	22.2770	37.5992	H	36.5319	22.8212	27.2378	N	37.9630	45.9144	22.7360
H	37.7933	21.3745	39.7211	H	40.7981	25.1177	28.2770	C	43.8327	44.4723	23.7637
H	36.5395	21.6237	41.8188	H	37.2993	20.9606	28.8861	C	45.0041	44.5700	23.0093
H	34.6294	22.2620	34.8863	H	38.3152	18.9210	28.6889	C	46.0251	43.6292	23.1873
H	36.2746	22.5929	33.0704	H	38.4629	16.6389	29.6237	C	45.8875	42.5765	24.0968
H	38.3473	25.5116	35.4809	H	40.1100	18.2579	33.2936	C	44.6887	42.4811	24.8259
H	36.6604	25.2607	37.2496	H	39.9588	20.5584	32.3502	C	43.6783	43.4211	24.6797
H	29.8073	27.3213	39.8453	H	39.6771	14.7829	30.5538	C	40.5412	50.9104	25.3180
H	27.5807	28.3621	39.6212	H	38.0111	14.9276	31.2758	C	40.3741	51.0596	26.7004
H	25.8330	24.4848	38.9036	H	39.3552	14.1814	32.2278	C	40.4968	52.3207	27.2835
H	28.0629	23.4322	39.1158	C	38.4382	24.3416	33.0878	C	40.7918	53.4477	26.5063
H	34.3772	20.1891	42.9314	C	38.9548	25.6492	32.9501	C	40.9053	53.2904	25.1184
H	35.4994	19.4937	45.0221	C	40.0292	25.8963	32.1183	C	40.7836	52.0342	24.5212
H	36.1552	23.6345	46.0589	C	40.5716	24.8341	31.3782	C	36.7077	45.3200	22.3161
H	35.0932	24.3341	43.9435	N	39.9765	23.5895	31.3764	C	36.3998	45.2117	20.9558
C	43.5615	21.0997	33.0049	C	38.9488	23.3515	32.2426	C	35.2141	44.5898	20.5693
C	44.2392	20.0899	32.3042	C	41.8038	24.8704	30.6274	C	34.3114	44.0940	21.5216
C	43.8321	19.7488	31.0208	N	42.0917	23.7406	29.9729	C	34.6382	44.2077	22.8782
C	42.7655	20.4457	30.4501	C	43.4124	23.5443	29.4709	C	35.8281	44.8140	23.2796
N	42.0462	21.3946	31.1357	C	44.5119	23.6844	30.3398	H	42.9621	50.3605	25.4917
C	42.4419	21.6991	32.4012	C	45.7687	23.3064	29.9189	H	44.9888	48.9712	25.3876
C	42.3435	20.2615	29.0608	C	45.9463	22.8069	28.6053	H	44.9195	46.6504	24.5818
N	41.4556	21.1125	28.6008	C	44.8635	22.7436	27.7126	H	38.1385	50.9128	24.5410

H	36.0567	50.0045	23.5900	C	48.5223	40.9017	14.9483	N	39.5157	49.5844	55.2846
H	35.9185	47.6844	22.7735	H	53.8923	42.9567	24.9895	N	37.2790	45.3956	56.4100
H	42.4128	43.0552	22.2884	H	52.7782	43.4601	22.8116	N	42.0446	45.9168	57.2727
H	40.2915	42.1985	21.3887	H	51.7328	39.2021	25.1206	C	36.1749	44.4720	56.2491
H	38.1930	43.4516	21.6354	H	51.1246	42.6923	20.9560	C	35.0043	44.5664	57.0051
H	45.1246	45.3738	22.2705	H	51.3164	41.6695	18.9165	C	33.9841	43.6250	56.8259
H	46.9426	43.7304	22.5989	H	50.3739	41.5215	16.6373	C	34.1216	42.5748	55.9135
H	44.5354	41.6503	25.5148	H	46.7031	39.8910	18.2708	C	35.3195	42.4829	55.1823
H	42.7633	43.3342	25.2651	H	47.6551	40.0396	20.5676	C	36.3289	43.4239	55.3295
H	40.1602	50.1925	27.3230	H	49.4271	40.3058	14.7830	C	39.4608	50.9126	54.6958
H	40.3814	52.4207	28.3615	H	48.7232	41.9791	14.9337	C	39.6262	51.0607	53.3130
H	41.0989	54.1644	24.4820	H	47.7534	40.6460	14.1909	C	39.5019	52.3211	52.7289
H	40.8901	51.9329	23.4324	C	46.9281	41.5647	24.3457	C	39.2069	53.4487	53.5053
H	37.0816	45.6160	20.1949	C	47.0711	41.0537	25.6551	C	39.0948	53.2925	54.8935
H	34.9908	44.4958	19.4970	C	47.9027	39.9794	25.9029	C	39.2181	52.0370	55.4917
H	33.9506	43.8365	23.6361	C	48.6375	39.4318	24.8395	C	43.3007	45.3220	57.6900
H	36.0655	44.8976	24.3385	N	48.6351	40.0223	23.5926	C	43.6117	45.2137	59.0496
C	47.0060	36.4336	21.0987	C	47.7684	41.0495	23.3541	C	44.7976	44.5905	59.4334
C	47.7066	35.7585	20.0872	C	49.3855	38.1982	24.8768	C	45.6978	44.0942	58.4791
C	48.9896	36.1672	19.7463	N	50.0353	37.9054	23.7452	C	45.3682	44.2082	57.1232
C	49.5603	37.2322	20.4458	C	50.5278	36.5810	23.5495	C	44.1776	44.8151	56.7245
N	48.8748	37.9487	21.3968	C	49.6530	35.4877	23.7003	H	37.0399	50.3625	54.5272
C	47.6095	37.5520	21.7008	C	50.0627	34.2266	23.3242	H	35.0134	48.9729	54.6347
C	50.9498	37.6541	20.2629	C	51.3709	34.0387	22.8146	H	35.0850	46.6515	55.4379
N	51.4098	38.5390	21.1168	C	52.2701	35.1155	22.7398	H	41.8637	50.9175	55.4743
C	52.7094	39.0701	20.8556	C	51.8459	36.3782	23.1155	H	43.9466	50.0100	56.4237
C	53.0287	39.4438	19.5251	O	51.6158	32.7815	22.4441	H	44.0876	47.6884	57.2362
C	54.2771	39.9212	19.2196	C	52.8725	32.4799	21.8251	H	37.5976	43.0531	57.7195
C	55.2613	40.0051	20.2422	H	46.5150	41.5108	26.4762	H	39.7205	42.1964	58.6160
C	54.9451	39.6480	21.5649	H	47.9969	39.5528	26.8998	H	41.8175	43.4524	58.3707
C	53.6713	39.1870	21.8641	H	47.7641	41.4501	22.3257	H	34.8838	45.3682	57.7460
O	56.4283	40.4450	19.7882	H	49.3484	37.5432	25.7407	H	33.0669	43.7239	57.4153
C	57.5719	40.4413	20.6440	H	48.6466	35.6468	24.0830	H	35.4730	41.6545	54.4908
H	47.2565	34.8953	19.5748	H	49.3993	33.3651	23.4013	H	37.2428	43.3400	54.7419
H	49.5543	35.6513	18.9524	H	53.2961	34.9631	22.3902	H	39.8401	50.1933	52.6908
H	47.0783	38.1454	22.4528	H	52.5440	37.2198	23.0740	H	39.6159	52.4201	51.6507
H	51.5396	37.2175	19.4359	H	53.0072	33.0497	20.8985	H	38.9010	54.1669	55.5292
H	52.2747	39.3508	18.7370	H	52.7481	31.4038	21.5947	H	39.1126	51.9365	56.5807
H	54.5556	40.2113	18.1927	H	53.7017	32.6250	22.5238	H	42.9317	45.6185	59.8119
H	55.7036	39.7047	22.3419	Fe	50.0650	39.2543	22.4112	H	45.0230	44.4960	60.5053
H	53.4376	38.8847	22.8836	C	39.6124	46.9729	56.3311	H	46.0536	43.8363	56.3637
H	57.8081	39.4300	20.9888	C	38.3928	47.4924	55.8495	H	43.9378	44.8986	55.6661
H	57.4552	41.1439	21.4688	C	38.3444	48.8243	55.3332	C	32.9996	36.4354	58.9065
H	58.3580	40.7944	19.9354	C	37.1157	49.3388	54.8945	C	32.2991	35.7604	59.9182
C	52.8688	41.0572	25.2440	C	35.9744	48.5471	54.9548	C	31.0151	36.1675	60.2573
C	53.1655	42.2487	24.5691	C	36.0052	47.2335	55.4222	C	30.4438	37.2313	59.5567
C	52.5419	42.5349	23.3573	C	37.2065	46.6928	55.8905	N	31.1295	37.9482	58.6062
C	51.6303	41.6184	22.8316	C	40.7800	47.7597	56.2956	C	32.3952	37.5526	58.3032
N	51.3469	40.4311	23.4617	C	40.7257	49.0974	55.7902	C	29.0534	37.6516	59.7375
C	51.9596	40.1664	24.6450	C	41.8807	49.8862	55.8285	N	28.5939	38.5366	58.8834
C	50.9326	41.7871	21.5522	C	43.0588	49.3628	56.3583	C	27.2937	39.0669	59.1433
N	50.1373	40.8071	21.1834	C	43.1445	48.0527	56.8244	C	26.9726	39.4398	60.4736
C	49.5601	40.8230	19.8877	C	42.0113	47.2326	56.8000	C	25.7236	39.9164	60.7779
C	50.3043	41.2723	18.7755	C	39.6566	45.6724	56.8771	C	24.7408	40.0010	59.7541
C	49.7831	41.1855	17.5014	C	38.4658	44.8901	56.9555	C	25.0590	39.6454	58.4315
C	48.4845	40.6700	17.3189	C	38.5045	43.6424	57.5879	C	26.3333	39.1848	58.1336
C	47.7153	40.2573	18.4403	C	39.7102	43.1620	58.0945	O	23.5729	40.4399	60.2067
C	48.2504	40.3366	19.7056	C	40.8958	43.8766	57.9725	C	22.4307	40.4366	59.3490
O	47.8569	40.5130	16.1619	C	40.8856	45.1478	57.3780	H	32.7501	34.8982	60.4316

H	30.4500	35.6509	61.0505	H	26.6961	34.9696	57.5923	H	40.1623	29.8063	52.6903
H	32.9262	38.1456	57.5507	H	27.4573	37.2241	56.9114	H	40.3848	27.5796	51.6494
H	28.4626	37.2137	60.5630	H	26.9664	33.0599	59.0869	H	41.0935	25.8299	55.5275
H	27.7258	39.3466	61.2626	H	27.2326	31.4113	58.3994	H	40.8832	28.0601	56.5803
H	25.4437	40.2055	61.8046	H	26.2892	32.6295	57.4560	H	37.0787	34.3878	59.8169
H	24.3017	39.7030	57.6534	Fe	29.9398	39.2535	57.5912	H	34.9866	35.5084	60.5116
H	26.5684	38.8838	57.1140	C	40.3902	33.0247	56.3320	H	33.9443	36.1508	56.3703
H	22.1939	39.4249	59.0057	C	41.6089	32.5047	55.8486	H	36.0598	35.0892	55.6710
H	22.5498	41.1373	58.5230	C	41.6564	31.1730	55.3317	C	47.0047	43.5603	58.8993
H	21.6438	40.7920	60.0553	C	42.8842	30.6584	54.8909	C	47.7066	44.2372	59.9090
C	27.1403	41.0577	54.7547	C	44.0260	31.4499	54.9502	C	48.9899	43.8289	60.2494
C	26.8440	42.2497	55.4288	C	43.9963	32.7629	55.4191	C	49.5596	42.7628	59.5511
C	27.4668	42.5360	56.6410	C	42.7957	33.3037	55.8889	N	48.8733	42.0449	58.6018
C	28.3765	41.6184	57.1684	C	39.2222	32.2384	56.2971	C	47.6077	42.4410	58.2985
N	28.6594	40.4305	56.5391	C	39.2755	30.9009	55.7912	C	50.9494	42.3409	59.7337
C	28.0480	40.1662	55.3550	C	38.1202	30.1128	55.8297	N	51.4085	41.4550	58.8805
C	29.0724	41.7867	58.4488	C	36.9425	30.6363	56.3601	C	52.7075	40.9223	59.1418
N	29.8658	40.8057	58.8193	C	36.8578	31.9461	56.8275	C	53.0258	40.5471	60.4720
C	30.4394	40.8209	60.1167	C	37.9916	32.7656	56.8027	C	54.2725	40.0648	60.7768
C	29.6926	41.2716	61.2267	C	40.3471	34.3249	56.8787	C	55.2558	39.9772	59.7538
C	30.2097	41.1829	62.5024	C	41.5384	35.1067	56.9561	C	54.9411	40.3378	58.4317
C	31.5061	40.6632	62.6891	C	41.5008	36.3544	57.5885	C	53.6690	40.8038	58.1332
C	32.2779	40.2497	61.5699	C	40.2954	36.8353	58.0954	O	56.4205	39.5297	60.2065
C	31.7476	40.3317	60.3028	C	39.1094	36.1214	57.9741	C	57.5632	39.5270	59.3495
O	32.1288	40.5026	63.8483	C	39.1187	34.8499	57.3805	H	47.2569	45.1013	60.4205
C	31.4603	40.8927	65.0597	N	40.4848	30.4135	55.2844	H	49.5555	44.3463	61.0418
H	26.1182	42.9582	55.0074	N	42.7243	34.6005	56.4096	H	47.0759	41.8465	57.5479
H	27.2311	43.4618	57.1859	N	37.9593	34.0812	57.2766	H	51.5398	42.7782	60.5599
H	28.2748	39.2016	54.8798	C	43.8289	35.5233	56.2472	H	52.2722	40.6426	61.2603
H	28.8802	42.6921	59.0446	C	44.9976	35.4315	57.0064	H	54.5503	39.7732	61.8035
H	28.6818	41.6708	61.0828	C	46.0187	36.3716	56.8252	H	55.6994	40.2798	57.6546
H	29.6169	41.5203	63.3645	C	45.8837	37.4176	55.9075	H	53.4364	41.1084	57.1141
H	33.2886	39.8804	61.7426	C	44.6877	37.5067	55.1731	H	57.8076	40.5378	59.0087
H	32.3453	40.0350	59.4424	C	43.6773	36.5672	55.3226	H	57.4396	38.8290	58.5219
H	30.5506	40.3029	65.2195	C	40.5386	29.0855	54.6954	H	58.3471	39.1643	60.0555
H	31.2668	41.9715	65.0757	C	40.3745	28.9384	53.3124	C	52.8700	38.9398	54.7540
H	32.2239	40.6301	65.8200	C	40.4978	27.6781	52.7278	C	53.1681	37.7487	55.4290
C	33.0816	41.5628	55.6629	C	40.7907	26.5498	53.5039	C	52.5439	37.4611	56.6401
C	32.9422	41.0506	54.3536	C	40.9014	26.7050	54.8923	C	51.6307	38.3763	57.1658
C	32.1114	39.9761	54.1044	C	40.7788	27.9604	55.4910	N	51.3458	39.5632	56.5356
C	31.3731	39.4295	55.1659	C	36.7036	34.6757	57.6949	C	51.9584	39.8287	55.3524
N	31.3719	40.0213	56.4122	C	36.3961	34.7887	59.0549	C	50.9330	38.2066	58.4449
C	32.2379	41.0487	56.6523	C	35.2098	35.4107	59.4395	N	50.1373	39.1860	58.8147
C	30.6248	38.1962	55.1274	C	34.3064	35.9020	58.4857	C	49.5608	39.1689	60.1107
N	29.9714	37.9046	56.2571	C	34.6326	35.7834	57.1293	C	50.3054	38.7174	61.2219
C	29.4759	36.5812	56.4522	C	35.8229	35.1768	56.7297	C	49.7850	38.8032	62.4964
C	30.3504	35.4865	56.3107	H	42.9593	29.6349	54.5230	C	48.4871	39.3199	62.6805
C	29.9362	34.2269	56.6868	H	44.9861	31.0238	54.6282	C	47.7176	39.7344	61.5602
C	28.6235	34.0418	57.1858	H	44.9169	33.3445	55.4347	C	48.2515	39.6561	60.2943
C	27.7251	35.1198	57.2509	H	38.1365	29.0815	55.4751	O	47.8606	39.4765	63.8382
C	28.1542	36.3813	56.8763	H	36.0543	29.9897	56.4253	C	48.5260	39.0845	65.0507
O	28.3740	32.7857	57.5570	H	35.9152	32.3105	57.2402	H	53.8960	37.0417	55.0089
C	27.1108	32.4867	58.1639	H	42.4080	36.9434	57.7192	H	52.7807	36.5358	57.1854
H	33.5001	41.5069	53.5333	H	40.2857	37.8011	58.6167	H	51.7296	40.7922	54.8763
H	32.0202	39.5488	53.1075	H	38.1879	36.5465	58.3721	H	51.1252	37.3008	59.0403
H	32.2390	41.4504	57.6803	H	45.1162	34.6324	57.7505	H	51.3169	38.3195	61.0797
H	30.6639	37.5407	54.2640	H	46.9343	36.2750	57.4173	H	50.3762	38.4654	63.3595
H	31.3598	35.6436	55.9351	H	44.5363	38.3317	54.4769	H	46.7057	40.1016	61.7307
H	30.5991	33.3642	56.6173	H	42.7647	36.6489	54.7328	H	47.6559	39.9544	59.4330

H	49.4336	39.6763	65.2154	C	36.3343	36.5762	24.6740	H	22.2058	40.5567	20.9707
H	48.7222	38.0062	65.0642	C	39.4640	29.0875	25.3169	H	22.5558	38.8373	21.4330
H	47.7592	39.3429	65.8093	C	39.6302	28.9399	26.6997	H	21.6564	39.2044	19.9018
C	46.9244	38.4288	55.6564	C	39.5058	27.6796	27.2842	C	27.1434	38.9421	25.2464
C	47.0670	38.9387	54.3466	C	39.2099	26.5519	26.5084	C	26.8483	37.7483	24.5749
C	47.8984	40.0129	54.0976	C	39.0975	26.7077	25.1202	C	27.4712	37.4599	23.3634
C	48.6338	40.5614	55.1602	C	39.2211	27.9629	24.5215	C	28.3808	38.3766	22.8341
N	48.6326	39.9710	56.4072	C	43.3029	34.6723	22.3111	N	28.6632	39.5659	23.4611
C	47.7661	38.9441	56.6469	C	43.6122	34.7813	20.9511	C	28.0509	39.8326	24.6442
C	49.3818	41.7950	55.1222	C	44.7980	35.4042	20.5663	C	29.0770	38.2062	21.5543
N	50.0328	42.0876	56.2531	C	45.6996	35.8996	21.5199	N	29.8709	39.1862	21.1823
C	50.5274	43.4113	56.4486	C	45.3718	35.7844	22.8761	C	30.4446	39.1691	19.8851
C	49.6524	44.5054	56.3053	C	44.1816	35.1777	23.2759	C	29.6985	38.7156	18.7759
C	50.0652	45.7652	56.6828	H	37.0439	29.6392	25.4881	C	30.2153	38.8027	17.4999
C	51.3766	45.9510	57.1850	H	35.0177	31.0291	25.3803	C	31.5111	39.3235	17.3124
C	52.2755	44.8734	57.2514	H	35.0886	33.3488	24.5720	C	32.2827	39.7397	18.4309
C	51.8480	43.6118	56.8757	H	41.8669	29.0821	24.5402	C	31.7523	39.6594	19.6981
O	51.6242	47.2069	57.5583	H	43.9493	29.9873	23.5880	O	32.1334	39.4831	16.1529
C	52.8860	47.5065	58.1678	H	44.0898	32.3067	22.7688	C	31.4653	39.0904	14.9422
H	46.5111	38.4810	53.5257	H	37.5989	36.9397	22.2766	H	26.1232	37.0402	24.9978
H	47.9922	40.4384	53.1002	H	39.7208	37.7932	21.3744	H	27.2362	36.5329	22.8204
H	47.7630	38.5436	57.6753	H	41.8185	36.5395	21.6238	H	28.2770	40.7983	25.1175
H	49.3442	42.4498	54.2581	H	34.8858	34.6293	22.2617	H	28.8849	37.2997	20.9601
H	48.6440	44.3479	55.9277	H	33.0699	36.2745	22.5925	H	28.6882	38.3155	18.9205
H	49.4016	46.6272	56.6123	H	35.4804	38.3474	25.5112	H	29.6230	38.4631	16.6384
H	53.3037	45.0242	57.5951	H	37.2490	36.6605	25.2603	H	33.2931	40.1096	18.2576
H	52.5452	42.7692	56.9122	H	39.8447	29.8074	27.3215	H	32.3496	39.9585	20.5580
H	53.0314	46.9299	59.0884	H	39.6206	27.5809	28.3624	H	30.5536	39.6771	14.7825
H	52.7613	48.5805	58.4078	H	38.9032	25.8330	24.4850	H	31.2753	38.0110	14.9271
H	53.7084	47.3688	57.4596	H	39.1154	28.0629	23.4324	H	32.2274	39.3549	14.1810
Fe	50.0634	40.7389	57.5872	H	42.9313	34.3772	20.1894	C	33.0872	38.4381	24.3411
C	39.6157	33.0255	23.6770	H	45.0221	35.4993	19.4942	C	32.9495	38.9546	25.6488
C	38.3965	32.5071	24.1608	H	46.0586	36.1551	23.6349	C	32.1179	40.0291	25.8959
C	38.3481	31.1760	24.6792	H	43.9431	35.0932	24.3345	C	31.3778	40.5716	24.8337
C	37.1197	30.6625	25.1195	C	33.0048	43.5613	21.0993	N	31.3759	39.9764	23.5891
C	35.9783	31.4543	25.0588	C	32.3042	44.2391	20.0894	C	32.2421	38.9488	23.3510
C	36.0090	32.7669	24.5887	C	31.0208	43.8321	19.7483	C	30.6271	41.8039	24.8699
C	37.2100	33.3065	24.1186	C	30.4500	42.7656	20.4453	N	29.9728	42.0919	23.7402
C	40.7832	32.2385	23.7134	N	31.1355	42.0463	21.3941	C	29.4710	43.4127	23.5438
C	40.7290	30.9017	24.2212	C	32.4011	42.4418	21.6987	C	30.3400	44.5121	23.6839
C	41.8838	30.1128	24.1841	C	29.0606	42.3438	20.2611	C	29.9193	45.7689	23.3060
C	43.0617	30.6348	23.6524	N	28.6005	41.4560	21.1121	C	28.6057	45.9467	22.8065
C	43.1472	31.9437	23.1827	C	27.3025	40.9236	20.8462	C	27.7128	44.8640	22.7433
C	42.0140	32.7640	23.2063	C	26.9872	40.5545	19.5135	C	28.1483	43.6053	23.1195
C	39.6594	34.3242	23.1270	C	25.7400	40.0775	19.2025	O	28.3499	47.2006	22.4326
C	38.4684	35.1061	23.0471	C	24.7528	39.9889	20.2217	C	27.0864	47.4919	21.8228
C	38.5061	36.3513	22.4098	C	25.0656	40.3397	21.5470	H	33.5092	38.5016	26.4697
C	39.7113	36.8300	21.9002	C	26.3381	40.8007	21.8516	H	32.0276	40.4595	26.8916
C	40.8972	36.1163	22.0243	O	23.5870	39.5521	19.7623	H	32.2424	38.5443	22.3243
C	40.8878	34.8472	22.6230	C	22.4415	39.5490	20.6155	H	30.6651	42.4609	25.7324
N	39.5191	30.4156	24.7278	H	32.7550	45.1029	19.5784	H	31.3502	44.3607	24.0596
N	37.2822	34.6023	23.5955	H	30.4559	44.3504	18.9561	H	30.5778	46.6351	23.3742
N	42.0469	34.0784	22.7296	H	32.9324	41.8463	22.4489	H	26.6830	45.0086	22.4015
C	36.1787	35.5267	23.7562	H	28.4712	42.7820	19.4347	H	27.4558	42.7588	23.0856
C	35.0075	35.4319	23.0014	H	27.7435	40.6507	18.7279	H	26.9466	46.9160	20.9008
C	33.9877	36.3738	23.1807	H	25.4648	39.7913	18.1737	H	27.2033	48.5673	21.5851
C	34.1267	37.4253	24.0915	H	24.3053	40.2781	22.3219	H	26.2640	47.3465	22.5294
C	35.3256	37.5179	24.8210	H	26.5688	41.0984	22.8733	Fe	29.9443	40.7408	22.4076

Table S3. Cartesian coordinates (in Å) for the PM7 model of helicate **2** (all Δ).

C	8.8780	22.5997	25.4156
C	9.0736	23.9505	25.7490
C	9.7464	24.8283	24.8452
C	9.9699	26.1504	25.2041
C	9.5161	26.6187	26.4497
C	8.8488	25.7990	27.3374
C	8.6125	24.4486	27.0025
C	9.3625	22.0940	24.1835
C	10.0083	22.9676	23.2638
C	10.4611	22.4599	22.0337
C	10.3042	21.1071	21.7546
C	9.7085	20.2279	22.6519
C	9.2037	20.7145	23.8701
C	8.1994	21.7519	26.3068
C	7.7021	22.2664	27.5399
C	6.9714	21.4166	28.3977
C	6.7496	20.1038	28.0336
C	7.2579	19.5687	26.8363
C	7.9947	20.3762	25.9812
N	10.1682	24.3034	23.6073
N	7.9454	23.5906	27.8616
N	8.5512	19.8906	24.7802
C	7.5445	24.0882	29.1610
C	8.4054	23.8933	30.2454
C	8.0488	24.4125	31.4817
C	6.8507	25.1288	31.6397
C	5.9916	25.2842	30.5407
C	6.3303	24.7660	29.2971
C	10.8082	25.2090	22.6890
C	10.0277	26.1394	21.9942
C	10.6574	27.0842	21.1903
C	12.0510	27.0941	21.0785
C	12.8166	26.1294	21.7428
C	12.1997	25.1819	22.5523
C	8.4863	18.4731	24.5404
C	7.6581	17.9681	23.5343
C	7.5911	16.5918	23.3379
C	8.3264	15.7226	24.1512
C	9.1446	16.2437	25.1618
C	9.2391	17.6165	25.3533
C	6.5257	25.7228	32.9269
C	7.5318	26.3557	33.6846
N	7.2950	26.9260	34.8708
C	6.0367	26.9026	35.3734
C	4.9738	26.2916	34.6790
C	5.2248	25.7006	33.4503
C	12.7232	28.1259	20.2852
C	13.7774	28.8751	20.8243
C	14.4301	29.8040	20.0234
C	14.0170	29.9584	18.6983
N	12.9494	29.2646	18.1722
C	12.3156	28.3670	18.9679
C	8.2403	14.2740	23.9544
C	7.0358	13.6408	23.6208
C	7.0215	12.2670	23.4068
C	8.2086	11.5470	23.5542
N	9.3879	12.1479	23.9367
C	9.3933	13.4916	24.1112
C	5.8775	27.5602	36.6874
N	4.6802	27.6940	37.1465
C	4.3792	28.3016	38.3700
C	3.0102	28.5293	38.6051
C	2.5888	29.1274	39.7844
C	3.5448	29.4904	40.7390
C	4.9206	29.2568	40.5272
C	5.3285	28.6687	39.3481
O	3.2943	30.0785	41.9243
C	1.9389	30.3701	42.2521
C	14.6729	30.8225	17.7165
N	14.1670	30.7717	16.5061
C	14.7854	31.5736	15.4905
C	15.5543	30.9572	14.5002
C	16.1439	31.7289	13.5077
C	15.9689	33.1203	13.5264
C	15.2091	33.7446	14.5433
C	14.6312	32.9709	15.5268
O	16.4594	33.9901	12.6357
C	17.3073	33.4989	11.5976
C	8.3564	10.1095	23.3184
N	9.5642	9.6363	23.5192
C	9.7920	8.2397	23.2880
C	10.5166	7.8491	22.1498
C	10.7718	6.5106	21.9349
C	10.2791	5.5527	22.8521
C	9.5293	5.9433	23.9698
C	9.2799	7.2921	24.1796
O	10.6160	4.2985	22.5262
C	10.0876	3.2294	23.3120
H	10.4956	26.8403	24.5469
H	9.7026	27.6699	26.7162
H	8.5047	26.1954	28.3018
H	10.9238	23.1140	21.3011
H	10.6552	20.7199	20.7925
H	9.6297	19.1751	22.3970
H	6.5729	21.8039	29.3443
H	6.1592	19.4496	28.6924
H	7.0485	18.5271	26.5991
H	9.3407	23.3491	30.1248
H	8.7050	24.2597	32.3445
H	5.0528	25.8346	30.6626
H	5.6636	24.8956	28.4447
H	8.9392	26.1412	22.1043
H	10.0587	27.8377	20.6746
H	13.9039	26.1260	21.6411
H	12.7947	24.4428	23.0945
H	7.0583	18.6450	22.9190
H	6.9506	16.1959	22.5460
H	9.6946	15.5749	25.8249
H	9.8767	18.0231	26.1427
H	8.5651	26.4227	33.3244
H	3.9714	26.2973	35.1222
H	4.4198	25.2123	32.9017
H	14.0769	28.7327	21.8695
H	15.2578	30.4081	20.4185
H	11.4661	27.8224	18.5388
H	6.1090	14.2216	23.5398
H	6.0917	11.7496	23.1347
H	10.3483	13.9584	24.3817
H	6.8150	27.8941	37.1569
H	2.2835	28.2336	37.8448
H	1.5297	29.3050	39.9536
H	5.6354	29.5451	41.2987
H	6.3888	28.4814	39.1761
H	1.5044	31.0772	41.5392
H	2.0460	30.8410	43.2429
H	1.3472	29.4527	42.3278
H	15.5305	31.4448	18.0092
H	15.7148	29.8805	14.5182
H	16.7550	31.2550	12.7403
H	15.1104	34.8348	14.5414
H	14.0524	33.4358	16.3300
H	16.7658	32.8216	10.9324
H	18.2111	33.0381	12.0085
H	17.5651	34.4362	11.0683
H	7.4965	9.5088	22.9910
H	10.8463	8.5938	21.4273
H	11.3219	6.1667	21.0550
H	9.1262	5.2027	24.6613
H	8.6882	7.6127	25.0419
H	10.4646	3.2695	24.3370
H	10.4974	2.3484	22.7833
H	8.9930	3.2142	23.2745
Fe	10.9182	10.8968	24.1731
C	18.1465	19.5478	20.1151
C	18.9397	20.3963	19.3244
C	18.3662	21.5386	18.6864
C	19.1558	22.3495	17.8823
C	20.5213	22.0556	17.7256
C	21.1079	20.9685	18.3419
C	20.3253	20.1136	19.1465
C	16.7762	19.8444	20.3205
C	16.2044	21.0008	19.7195
C	14.8604	21.3199	19.9782
C	14.1179	20.5067	20.8264
C	14.6481	19.3581	21.4042
C	15.9817	18.9992	21.1449
C	18.7142	18.4042	20.7015
C	20.1007	18.1260	20.5166
C	20.6562	16.9647	21.0962
C	19.8453	16.0996	21.8009
C	18.4740	16.3516	21.9875
C	17.9091	17.5112	21.4746
N	16.9985	21.7964	18.9033
N	20.8674	18.9982	19.7650
N	16.5550	17.8481	21.6788
C	22.2874	18.7544	19.6208
C	22.7359	17.8967	18.6130
C	24.1031	17.7186	18.4492

C	25.0185	18.3957	19.2714	C	9.0752	7.6788	27.7456	C	8.3290	19.1310	14.0333
C	24.5384	19.2244	20.2985	C	9.9772	8.6157	27.2601	C	8.4771	20.5267	14.2988
C	23.1747	19.4084	20.4808	O	6.7156	7.0831	27.9671	C	7.8305	21.4543	13.4938
C	16.4034	22.8864	18.1751	C	7.0582	5.9205	28.7226	C	7.0542	21.0121	12.4081
C	16.6499	24.2025	18.5795	H	18.7458	23.2133	17.3627	C	6.9108	19.6718	12.1128
C	16.1623	25.2500	17.8040	H	21.1326	22.7156	17.0918	C	7.5468	18.7044	12.9206
C	15.4295	24.9838	16.6436	H	22.1782	20.7668	18.2021	C	9.6994	18.5996	15.9880
C	15.1502	23.6624	16.2763	H	14.4072	22.2044	19.5405	C	9.8873	19.9885	16.2369
C	15.6360	22.6069	17.0394	H	13.0827	20.7828	21.0553	C	10.6823	20.3910	17.3239
C	15.7555	16.9745	22.4952	H	14.0306	18.7648	22.0735	C	11.2352	19.4274	18.1600
C	16.0817	16.8020	23.8449	H	21.7266	16.7512	20.9800	C	11.0293	18.0665	17.9622
C	15.3450	15.9080	24.6146	H	20.2750	15.1854	22.2365	C	10.2756	17.6317	16.8586
C	14.2628	15.2149	24.0598	H	17.8840	15.6189	22.5341	C	8.7834	16.8104	14.5955
C	13.9252	15.4283	22.7165	H	22.0303	17.3805	17.9634	C	8.0350	16.3910	13.4569
C	14.6756	16.2903	21.9252	H	24.4727	17.0411	17.6726	C	7.9348	15.0138	13.1654
C	26.4489	18.2680	19.0417	H	25.2485	19.7446	20.9498	C	8.5565	14.0938	13.9854
C	26.9570	18.2570	17.7272	H	22.8070	20.0594	21.2736	C	9.2768	14.4849	15.1284
N	28.2647	18.1817	17.4563	H	17.2416	24.4040	19.4775	C	9.3826	15.8318	15.4460
C	29.1471	18.1124	18.4821	H	16.3839	26.2801	18.0905	N	9.2838	20.9091	15.3896
C	28.7211	18.1035	19.8254	H	14.5616	23.4538	15.3805	N	7.4273	17.3487	12.6633
C	27.3651	18.1814	20.1006	H	15.4420	21.5733	16.7431	N	10.0764	16.2832	16.5875
C	14.9538	26.0776	15.7937	H	16.9179	17.3551	24.2887	C	6.6108	16.9271	11.5445
C	15.1598	26.0602	14.4076	H	15.6322	15.7434	25.6586	C	5.2741	16.5895	11.7755
C	14.6188	27.0741	13.6271	H	13.0675	14.9218	22.2762	C	4.4793	16.2357	10.6943
C	13.8773	28.0810	14.2497	H	14.4294	16.4293	20.8716	C	5.0039	16.2268	9.3914
N	13.7195	28.1413	15.6170	H	26.2962	18.3267	16.8551	C	6.3579	16.5397	9.1924
C	14.2624	27.1503	16.3681	H	29.4693	18.0406	20.6236	C	7.1690	16.8900	10.2638
C	13.4857	14.2767	24.8732	H	27.0132	18.1652	21.1318	C	9.4392	22.3193	15.6333
C	13.2959	14.4726	26.2487	H	15.7458	25.2559	13.9476	C	10.2481	23.0738	14.7761
C	12.5250	13.5707	26.9718	H	14.7633	27.0870	12.5391	C	10.3088	24.4530	14.9435
C	11.9118	12.5135	26.2968	H	14.1309	27.2105	17.4552	C	9.5800	25.0723	15.9647
N	12.0830	12.3096	24.9450	H	13.7506	15.3318	26.7539	C	8.8109	24.3002	16.8425
C	12.8958	13.1575	24.2674	H	12.3888	13.6864	28.0546	C	8.7337	22.9210	16.6800
C	30.5695	18.0516	18.0843	H	13.0810	12.9306	23.2095	C	10.5277	15.2897	17.5259
N	31.4514	18.1921	19.0140	H	30.7470	17.9074	17.0081	C	11.8906	15.0023	17.6424
C	32.8318	18.1770	18.7889	H	33.1334	18.8124	20.8330	C	12.2989	14.0030	18.5204
C	33.6227	18.5474	19.8929	H	35.6077	18.8768	20.6433	C	11.3563	13.2868	19.2664
C	35.0060	18.5820	19.7873	H	35.3259	17.5613	16.5306	C	9.9952	13.5892	19.1435
C	35.6011	18.2321	18.5706	H	32.8426	17.5085	16.7220	C	9.5750	14.5952	18.2815
C	34.8255	17.8388	17.4590	H	37.6332	19.6398	19.6685	C	4.1420	15.9231	8.2601
C	33.4511	17.8151	17.5733	H	38.7905	18.5148	18.8641	C	2.8319	16.4407	8.2113
O	36.9225	18.2242	18.3116	H	37.7427	17.9109	20.2022	N	1.9995	16.2108	7.1900
C	37.8100	18.6053	19.3591	H	13.2062	29.2200	12.4542	C	2.4243	15.4498	6.1526
C	13.1536	29.1410	13.5484	H	9.7978	30.0133	14.0232	C	3.7176	14.8908	6.1255
N	12.4538	29.9401	14.3211	H	8.4889	31.8426	12.9877	C	4.5764	15.1322	7.1864
C	11.7003	30.9871	13.6945	H	12.1576	33.9694	12.0997	C	9.6072	26.5293	16.1122
C	10.3076	30.8938	13.6355	H	13.4724	32.1187	13.1559	C	8.4329	27.2634	16.3267
C	9.5741	31.9162	13.0480	H	7.7470	34.1039	12.7136	C	8.5140	28.6374	16.5183
C	10.2484	33.0219	12.5101	H	7.9418	33.2679	11.1148	C	9.7688	29.2502	16.4896
C	11.6600	33.1032	12.5489	H	8.1217	35.0595	11.2266	N	10.9244	28.5478	16.2268
C	12.3798	32.0807	13.1288	H	10.8337	11.5459	27.9931	C	10.8303	27.2074	16.0426
O	9.6826	34.0875	11.9309	H	7.7798	10.9064	25.9792	C	11.7944	12.2184	20.1674
C	8.2665	34.0963	11.7522	H	6.1487	9.2119	26.8279	C	12.7981	11.3135	19.7952
C	11.0012	11.5448	26.9074	H	9.4406	6.7965	28.2723	C	13.2274	10.3567	20.7068
N	10.4348	10.7115	26.0657	H	11.0512	8.4644	27.4002	C	12.6366	10.3180	21.9718
C	9.5082	9.7499	26.5917	H	7.6564	5.2258	28.1278	N	11.6055	11.1596	22.3276
C	8.1312	9.9859	26.4418	H	6.0588	5.4929	28.9274	C	11.2042	12.0954	21.4322
C	7.2276	9.0590	26.9180	H	7.5513	6.1903	29.6628	C	1.4358	15.2569	5.0710
C	7.7023	7.8969	27.5712	C	8.9353	18.1796	14.8706	N	1.8430	14.6876	3.9881

C	1.0250	14.4459	2.8798	H	7.9004	22.5237	13.6829	H	14.0166	9.6401	20.4436
C	1.6967	14.0034	1.7247	H	6.5501	21.7632	11.7818	H	10.3925	12.7660	21.7396
C	0.9909	13.7314	0.5612	H	6.3007	19.3580	11.2555	H	0.4266	15.6375	5.2883
C	-0.3985	13.8942	0.5587	H	10.8720	21.4436	17.5117	H	2.7818	13.8810	1.7544
C	-1.0912	14.3219	1.7116	H	11.8593	19.7491	19.0005	H	1.5195	13.3957	-0.3274
C	-0.3784	14.5965	2.8602	H	11.4620	17.3524	18.6566	H	-2.1761	14.4269	1.6740
O	-1.2174	13.6753	-0.4876	H	7.3713	14.6786	12.2849	H	-0.9043	14.9238	3.7577
C	-0.6346	13.2384	-1.7119	H	8.4918	13.0216	13.7462	H	0.0580	13.9872	-2.1078
C	10.0172	30.6652	16.7655	H	9.7493	13.7157	15.7369	H	-1.5205	13.1534	-2.3620
N	11.2809	31.0218	16.7414	H	4.8595	16.6071	12.7825	H	-0.1544	12.2624	-1.5919
C	11.6008	32.3878	17.0356	H	3.4332	15.9566	10.8576	H	9.1820	31.3435	16.9856
C	12.2024	32.6945	18.2675	H	6.7730	16.5231	8.1793	H	12.3732	31.9113	19.0039
C	12.5410	34.0017	18.5492	H	8.2178	17.1389	10.1038	H	12.9996	34.2816	19.5013
C	12.2621	35.0137	17.5998	H	10.8008	22.5875	13.9673	H	11.3999	35.4910	15.6602
C	11.6349	34.7079	16.3838	H	10.9029	25.0517	14.2507	H	10.7977	33.1375	15.1702
C	11.2948	33.3913	16.1105	H	8.2550	24.7781	17.6524	H	12.9143	37.2502	16.1931
O	12.6631	36.2265	17.9978	H	8.1108	22.3183	17.3456	H	12.8131	38.1747	17.7421
C	12.3901	37.3428	17.1485	H	12.6234	15.5422	17.0362	H	11.3124	37.4846	17.0163
C	13.0448	9.4334	23.0626	H	13.3633	13.7735	18.6131	H	13.8589	8.7105	22.9168
N	12.3833	9.5967	24.1861	H	9.2512	13.0185	19.7019	H	13.5539	10.4783	26.4088
C	12.7569	8.7890	25.3079	H	8.5116	14.8260	18.1766	H	14.1881	9.0861	28.3885
C	13.3512	9.4085	26.4204	H	2.4351	17.0760	9.0120	H	12.7489	5.5503	26.3414
C	13.7100	8.6433	27.5106	H	4.0186	14.2797	5.2670	H	12.1046	6.9388	24.3907
C	13.4865	7.2461	27.4841	H	5.5770	14.7007	7.1910	H	12.7530	4.8739	28.6193
C	12.9065	6.6292	26.3670	H	7.4600	26.7582	16.3358	H	14.2106	5.0136	29.6773
C	12.5517	7.4054	25.2734	H	7.6108	29.2369	16.6909	H	14.4177	4.7443	27.9057
O	13.8735	6.6360	28.6109	H	11.7587	26.6592	15.8418	Fe	12.5838	29.6412	16.2541
C	13.7911	5.2114	28.6730	H	13.2347	11.3557	18.7902				

Table S4. Cartesian coordinates (in Å) for the PM7 model of tetrahedron **3** (all Δ).

C	24.1336	20.7319	11.8124	H	32.4685	27.5063	9.1645	H	34.1852	22.6375	11.3145
C	24.6191	20.5946	10.5057	Fe	26.8063	23.7129	13.4103	H	34.6184	21.0934	10.4803
C	25.7057	21.3621	10.1045	C	24.3377	22.6478	16.7849	H	34.3096	21.0890	12.2588
C	26.2712	22.2580	11.0166	C	25.2090	21.8335	17.5194	C	23.0868	25.8057	12.7744
N	25.8656	22.3267	12.3322	C	26.4895	21.6023	17.0321	C	23.4452	27.1160	13.1184
C	24.8174	21.5527	12.7151	C	26.8639	22.1824	15.8165	C	24.7479	27.3763	13.5236
C	27.2777	23.2643	10.6787	N	26.0444	23.0521	15.1292	C	25.6576	26.3163	13.5903
N	27.5876	24.0921	11.6507	C	24.8026	23.2824	15.6290	N	25.3432	25.0452	13.1604
C	28.5180	25.1406	11.3536	C	28.1119	21.8977	15.1091	C	24.0707	24.8131	12.7433
C	28.0554	26.4674	11.3087	N	28.2300	22.4672	13.9310	C	26.9890	26.4030	14.1884
C	28.9385	27.4847	11.0181	C	29.4132	22.1771	13.1760	N	27.6392	25.2639	14.2725
C	30.2960	27.1777	10.7483	C	29.3076	21.3816	12.0218	C	28.9163	25.2809	14.9218
C	30.7496	25.8496	10.7756	C	30.4400	21.0886	11.2932	C	29.0512	24.6453	16.1686
C	29.8541	24.8331	11.0666	C	31.6985	21.5758	11.7282	C	30.2705	24.6603	16.8104
O	31.0323	28.2591	10.4886	C	31.8019	22.3552	12.8911	C	31.3666	25.3343	16.2151
C	32.3960	28.0821	10.0938	C	30.6580	22.6429	13.6184	C	31.2237	25.9812	14.9775
H	24.1395	19.9015	9.8065	O	32.7009	21.2168	10.9253	C	29.9933	25.9624	14.3404
H	26.1104	21.2822	9.0840	C	34.0385	21.5546	11.3047	O	32.4789	25.2649	16.9474
H	24.5178	21.5922	13.7674	H	24.8813	21.3784	18.4597	C	33.6336	25.9903	16.5145
H	27.6986	23.3110	9.6618	H	27.1994	20.9666	17.5828	H	22.7039	27.9205	13.0796
H	27.0007	26.6839	11.4629	H	24.1657	23.9943	15.0946	H	25.0630	28.3929	13.8024
H	28.6127	28.5272	10.9563	H	28.8646	21.2293	15.5563	H	23.8345	23.8091	12.3767
H	31.7914	25.6104	10.5493	H	28.3449	20.9685	11.7292	H	27.3709	27.3669	14.5596
H	30.1913	23.7921	11.0790	H	30.4008	20.4585	10.3996	H	28.1888	24.1800	16.6404
H	32.9878	27.6429	10.9008	H	32.7731	22.7163	13.2374	H	30.4104	24.1904	17.7883
H	32.7048	29.1313	9.9142	H	30.7260	23.2385	14.5338	H	32.0642	26.5135	14.5259

H	29.8650	26.4728	13.3809	C	12.5105	19.1898	5.1522	C	21.7937	20.8800	12.5850
H	34.0104	25.6044	15.5639	C	13.4549	18.9524	4.1448	C	20.5752	20.2997	12.9203
H	34.3460	25.7652	17.3333	C	13.2038	17.9628	3.2031	C	16.2359	15.2989	17.6885
H	33.4347	27.0670	16.4765	C	12.0207	17.2238	3.3012	C	16.6989	13.9925	17.8700
C	7.8452	18.5249	7.4794	N	11.0407	17.5176	4.2247	C	16.6112	13.4110	19.1332
C	6.8947	19.3256	6.8332	C	11.2919	18.5054	5.1233	C	16.0629	14.1286	20.1984
C	6.7330	19.2065	5.4582	C	11.7215	16.0287	2.5141	C	15.5738	15.4250	19.9937
C	7.5377	18.3009	4.7605	N	10.6121	15.4054	2.8430	C	15.6597	16.0196	18.7391
N	8.4038	17.4394	5.3991	C	10.3139	14.1871	2.1505	H	19.4328	19.0284	10.6299
C	8.5302	17.5501	6.7470	C	10.3780	12.9717	2.8544	H	17.7800	18.5587	8.8628
C	7.6218	18.2150	3.3024	C	10.1102	11.7910	2.1961	H	15.6939	17.3446	9.3465
N	8.5347	17.3895	2.8431	C	9.7930	11.8160	0.8145	H	20.8420	18.6111	15.3431
C	8.7073	17.3189	1.4221	C	9.7425	13.0304	0.1124	H	20.4176	17.7507	17.6107
C	9.8818	17.8417	0.8530	C	10.0162	14.2122	0.7818	H	18.3621	16.5037	18.1476
C	10.0599	17.7823	-0.5123	O	9.5602	10.6028	0.3121	H	12.8308	14.9422	12.5772
C	9.0484	17.2132	-1.3264	C	9.3305	10.4748	-1.0944	H	12.4485	14.1273	14.8705
C	7.8675	16.7126	-0.7564	H	14.3836	19.5313	4.1090	H	14.0658	14.5642	16.6754
C	7.6962	16.7790	0.6171	H	13.9242	17.7486	2.3993	H	12.7078	17.5779	11.0224
O	9.3610	17.2156	-2.6230	H	10.4994	18.7546	5.8360	H	11.1806	16.8299	9.2122
C	8.3852	16.7604	-3.5651	H	12.4180	15.6890	1.7314	H	13.8189	13.5249	8.3305
H	6.2969	20.0449	7.4026	H	10.6758	12.9612	3.9005	H	15.3508	14.2645	10.1447
H	5.9968	19.8215	4.9188	H	10.1677	10.8243	2.7051	H	21.4493	17.0010	12.5511
H	9.1959	16.8430	7.2522	H	9.5118	13.0487	-0.9554	H	23.6193	18.0576	11.9486
H	6.9693	18.8377	2.6701	H	9.9934	15.1649	0.2438	H	21.8890	21.9657	12.5820
H	10.6256	18.3246	1.4828	H	8.4049	10.9749	-1.3906	H	19.7199	20.9184	13.1844
H	10.9520	18.1944	-0.9933	H	9.2259	9.3760	-1.1946	H	17.1365	13.4322	17.0355
H	7.0780	16.2936	-1.3846	H	10.1941	10.8229	-1.6717	H	16.9806	12.3880	19.2882
H	6.7747	16.4040	1.0727	C	16.8284	16.9088	13.8061	H	15.1452	15.9834	20.8261
H	8.1657	15.6990	-3.4257	C	17.0700	17.3701	12.4966	H	15.3002	17.0343	18.5804
H	8.9198	16.9221	-4.5224	C	18.2753	18.0717	12.1971	C	14.8386	24.4430	11.3870
H	7.4821	17.3799	-3.5318	C	18.5112	18.5046	10.8855	C	15.2803	25.3926	12.3302
Fe	9.4712	16.2888	4.1709	C	17.5741	18.2347	9.8922	C	16.6700	25.5088	12.6303
C	11.4472	14.6688	7.6482	C	16.3930	17.5469	10.1577	C	17.0927	26.4630	13.5650
C	10.7790	13.4468	7.8013	C	16.1325	17.0944	11.4575	C	16.1555	27.2918	14.1761
C	9.7777	13.1068	6.9009	C	17.7728	17.1511	14.8238	C	14.7962	27.2101	13.8873
C	9.4580	14.0044	5.8776	C	18.9766	17.8564	14.5281	C	14.3435	26.2710	12.9514
N	10.1681	15.1663	5.6647	C	19.9118	18.0809	15.5472	C	15.7683	23.5951	10.7523
C	11.1612	15.4722	6.5403	C	19.6653	17.5909	16.8264	C	17.1586	23.7094	11.0505
C	8.3150	13.8613	4.9773	C	18.5051	16.8864	17.1369	C	18.0722	22.8640	10.4082
N	8.1146	14.8714	4.1612	C	17.5492	16.6508	16.1404	C	17.6144	21.9440	9.4688
C	6.9730	14.7998	3.2981	C	15.6535	16.1851	14.0930	C	16.2656	21.8276	9.1447
C	5.8935	15.6729	3.5197	C	14.7137	15.9092	13.0561	C	15.3265	22.6542	9.7754
C	4.7852	15.6033	2.7035	C	13.5576	15.1762	13.3552	C	13.4699	24.3556	11.0628
C	4.7396	14.6394	1.6647	C	13.3496	14.7160	14.6526	C	12.5325	25.2386	11.6762
C	5.8129	13.7590	1.4566	C	14.2582	14.9602	15.6788	C	11.1783	25.1530	11.3279
C	6.9217	13.8345	2.2840	C	15.4262	15.6854	15.4099	C	10.7681	24.2186	10.3808
O	3.6099	14.6879	0.9578	N	19.1924	18.2965	13.2233	C	11.6640	23.3514	9.7620
C	3.3921	13.7021	-0.0562	N	14.9809	16.3714	11.7673	C	13.0257	23.4172	10.0848
H	11.0366	12.7745	8.6262	N	16.3822	15.9293	16.3947	N	17.5710	24.6692	11.9744
H	9.2326	12.1550	6.9908	C	14.1075	15.9618	10.6892	N	12.9977	26.1729	12.6020
H	11.7417	16.3802	6.3486	C	12.9479	16.6981	10.4288	N	13.9668	22.5930	9.4693
H	7.6753	12.9660	5.0288	C	12.0985	16.2765	9.4108	C	12.0694	27.1605	13.1080
H	5.9176	16.3703	4.3542	C	12.4007	15.1306	8.6645	C	11.3144	26.8697	14.2482
H	3.9185	16.2539	2.8556	C	13.5735	14.4168	8.9230	C	10.3900	27.8066	14.6984
H	5.7722	13.0056	0.6665	C	14.4345	14.8294	9.9374	C	10.2159	29.0166	14.0144
H	7.7592	13.1449	2.1413	C	20.4629	18.9062	12.8972	C	10.9969	29.3045	12.8930
H	4.1206	13.7995	-0.8651	C	21.5479	18.0929	12.5588	C	11.9299	28.3771	12.4338
H	2.3799	13.9805	-0.4118	C	22.7616	18.6881	12.2205	C	18.9875	24.9123	12.1415
H	3.3749	12.6922	0.3682	C	22.8853	20.0793	12.2245	C	19.6505	25.7387	11.2292

C	21.0033	26.0119	11.4190	H	13.6060	23.3531	6.9188	C	12.6402	28.3162	17.9169
C	21.6841	25.4624	12.5081	H	12.9508	21.8755	5.0282	C	13.3116	27.0996	17.9738
C	21.0140	24.6108	13.3952	H	12.7405	18.5129	7.7323	C	14.7321	20.0094	21.7533
C	19.6621	24.3323	13.2199	H	13.3887	19.9826	9.6257	C	15.0564	20.0345	23.1128
C	13.5423	21.7439	8.3778	H	6.9661	26.7603	20.7893	C	14.5427	19.0466	23.9501
C	13.4134	22.2887	7.0968	H	6.6104	28.9684	21.9348	C	13.7189	18.0460	23.4301
C	13.0484	21.4581	6.0397	H	8.0745	28.8987	17.2012	C	13.4226	18.0204	22.0615
C	12.8151	20.0991	6.2638	H	9.8165	12.2096	19.6361	C	13.9243	19.0033	21.2148
C	12.9205	19.5739	7.5580	H	10.4497	10.2261	21.0430	C	12.0881	30.5410	18.8333
C	13.2868	20.3913	8.6223	H	11.7276	14.7728	22.5444	C	12.8095	31.7359	18.7210
C	7.6348	27.6842	18.9425	H	6.9781	31.5756	21.8457	C	12.1254	32.9150	18.4496
C	7.1685	27.6988	20.2633	H	9.9235	33.7677	20.5714	C	10.7382	32.8679	18.2842
C	6.9771	28.9193	20.8984	H	10.0346	36.0466	21.5386	N	10.0105	31.7145	18.4850
C	7.2716	30.0955	20.2020	H	5.7070	36.1767	21.7344	C	10.6913	30.5753	18.7726
N	7.6481	30.0953	18.8762	H	5.6057	33.8746	20.7563	C	13.1214	17.0299	24.3047
C	7.8066	28.8935	18.2625	H	9.5561	38.4105	21.6322	C	12.1818	17.3825	25.2815
C	10.8198	13.6184	20.9473	H	9.5422	37.6641	23.2882	C	11.5351	16.3777	25.9907
C	10.4033	12.3413	20.5510	H	8.5369	39.1243	22.9442	C	11.8358	15.0443	25.6976
C	10.7587	11.2438	21.3258	H	11.9597	9.3275	23.0172	N	12.8205	14.6896	24.8004
C	11.5365	11.4495	22.4693	H	15.5662	10.2830	24.1010	C	13.4574	15.6840	24.1297
N	11.8658	12.7096	22.9205	H	16.7938	8.5622	25.4373	C	9.9155	33.9841	17.8176
C	11.4878	13.7739	22.1659	H	13.0506	7.3069	27.2228	N	8.6483	33.7004	17.6201
C	7.2827	31.4310	20.7968	H	11.8461	9.0342	25.9144	C	7.8064	34.7491	17.1216
N	7.6970	32.3943	20.0047	H	14.6717	6.4643	28.7561	C	7.3376	34.6960	15.8056
C	7.7627	33.7137	20.5611	H	16.0634	5.4312	28.2419	C	6.5231	35.7108	15.3238
C	9.0064	34.3101	20.7909	H	14.5077	5.3490	27.3303	C	6.1904	36.7860	16.1645
C	9.0683	35.5828	21.3398	Fe	13.0266	12.7260	24.5394	C	6.6830	36.8467	17.4913
C	7.8768	36.2513	21.6686	C	16.2984	23.1974	19.4286	C	7.4986	35.8401	17.9568
C	6.6186	35.6371	21.4503	C	16.8493	24.2868	18.7246	O	5.4111	37.8223	15.8528
C	6.5677	34.3704	20.9147	C	16.1055	25.4958	18.5834	C	4.9205	37.9435	14.5163
O	7.7724	37.4739	22.1891	C	16.6768	26.5786	17.9021	C	11.1187	13.8901	26.2393
C	8.9630	38.1902	22.5230	C	17.9639	26.4645	17.3837	N	11.4873	12.7257	25.7554
C	12.1538	10.3841	23.2595	C	18.7112	25.2967	17.5106	C	10.7776	11.5668	26.2105
N	12.9556	10.7927	24.2167	C	18.1700	24.1992	18.1924	C	9.9237	10.8975	25.3164
C	13.6457	9.7931	24.9766	C	15.0074	23.2979	19.9848	C	9.2319	9.7846	25.7429
C	15.0366	9.6615	24.8196	C	14.2599	24.5042	19.8417	C	9.3754	9.3413	27.0819
C	15.7147	8.7060	25.5451	C	12.9836	24.5949	20.4126	C	10.2137	10.0243	27.9769
C	14.9980	7.8554	26.4242	C	12.4736	23.5179	21.1319	C	10.9034	11.1440	27.5399
C	13.6063	7.9758	26.5615	C	13.1877	22.3339	21.2958	O	8.6537	8.2522	27.3490
C	12.9326	8.9378	25.8261	C	14.4642	22.2110	20.7317	C	8.6276	7.7567	28.6908
O	15.7833	6.9874	27.0636	C	17.0476	22.0155	19.5969	H	16.1343	27.5187	17.7973
C	15.1753	6.0016	27.9036	C	18.3676	21.9245	19.0644	H	18.4113	27.3285	16.8734
H	18.1502	26.5775	13.8038	C	19.1054	20.7486	19.2547	H	19.7204	25.2558	17.1011
H	16.5008	28.0447	14.8971	C	18.5481	19.6934	19.9717	H	12.3968	25.5089	20.3235
H	14.0993	27.8929	14.3727	C	17.2661	19.7614	20.5100	H	11.4838	23.6121	21.5992
H	19.1392	22.9342	10.6183	C	16.5056	20.9256	20.3405	H	12.7584	21.5221	21.8833
H	18.3430	21.3018	8.9558	N	14.8291	25.5665	19.1410	H	20.1210	20.6600	18.8685
H	15.9540	21.1094	8.3858	N	18.8899	23.0191	18.3752	H	19.1441	18.7850	20.1324
H	10.4455	25.8261	11.7735	N	15.2297	21.0554	20.8868	H	16.8757	18.9191	21.0806
H	9.7070	24.1750	10.1001	C	20.2729	22.9713	17.9535	H	19.8111	21.9728	16.0904
H	11.3020	22.6463	9.0139	C	20.5935	22.3787	16.7283	H	22.1946	21.8227	15.4058
H	11.4371	25.9196	14.7643	C	21.9295	22.3006	16.3488	H	23.3824	23.8196	19.0476
H	9.7812	27.5830	15.5746	C	22.9339	22.8042	17.1850	H	20.9981	23.9707	19.7418
H	10.8743	30.2601	12.3653	C	22.5967	23.4123	18.3965	H	14.8747	27.5071	21.0020
H	12.5361	28.5995	11.5482	C	21.2624	23.5002	18.7874	H	13.6701	29.6806	20.8799
H	19.1148	26.1772	10.3794	C	14.1149	26.8232	19.0844	H	12.0244	28.5509	17.0486
H	21.5304	26.6665	10.7115	C	14.2460	27.7370	20.1338	H	13.2229	26.3790	17.1634
H	21.5452	24.1814	14.2448	C	13.5708	28.9539	20.0618	H	15.6992	20.8231	23.5214
H	19.1340	23.6866	13.9186	C	12.7733	29.2484	18.9540	H	14.7861	19.0603	25.0214

H	12.7775	17.2400	21.6582	C	7.5328	18.8276	11.2240	O	13.2095	12.2287	31.3727
H	13.6825	18.9979	20.1539	C	7.2142	18.5632	9.8936	C	13.4866	11.0768	32.1747
H	13.8988	31.7384	18.8330	C	8.1743	18.7373	8.8943	H	8.4501	24.5111	14.7333
H	12.6615	33.8710	18.3513	C	9.4664	19.1617	9.2306	H	8.1499	24.0149	12.3404
H	10.1058	29.6690	18.9576	C	9.7972	19.4327	10.5542	H	8.4615	21.7380	11.4508
H	11.9538	18.4356	25.4762	C	8.7282	23.9895	17.1659	H	9.4580	22.9315	19.3038
H	10.7902	16.6194	26.7641	C	9.7139	24.9429	17.4382	H	10.0466	21.0849	20.8237
H	14.2561	15.3975	23.4381	C	9.3360	26.1595	17.9976	H	10.3703	18.7900	19.9919
H	10.3691	34.9704	17.6318	C	7.9906	26.4197	18.2861	H	9.5060	16.9762	12.2446
H	7.6348	33.8804	15.1497	C	7.0142	25.4671	17.9863	H	10.0537	15.1639	13.8177
H	6.1689	35.6855	14.2931	C	7.3780	24.2461	17.4227	H	10.3565	15.5996	16.2250
H	6.4226	37.7094	18.1175	C	10.3020	16.9222	18.3377	H	6.7768	18.6962	12.0068
H	7.9035	35.8818	18.9717	C	11.5922	16.4812	18.6473	H	6.2033	18.2196	9.6342
H	4.2370	37.1278	14.2699	C	11.7435	15.4101	19.5215	H	10.2137	19.3042	8.4498
H	5.7408	38.0283	13.7966	C	10.6202	14.7825	20.0752	H	10.7951	19.7785	10.8164
H	4.3699	38.9047	14.5791	C	9.3383	15.2424	19.7667	H	10.7606	24.7296	17.2305
H	10.3161	14.0312	26.9803	C	9.1722	16.3178	18.8963	H	10.0975	26.9031	18.2339
H	9.7811	11.2788	24.3077	C	9.1672	29.9387	14.4672	H	5.9566	25.6744	18.1997
H	8.5446	9.2477	25.0824	C	8.0447	30.1912	13.6674	H	6.6122	23.4957	17.1952
H	10.3090	9.6948	29.0142	C	6.9899	30.9247	14.1951	H	12.4616	16.9607	18.2020
H	11.5507	11.6934	28.2304	C	7.0758	31.3740	15.5167	H	12.7435	15.0459	19.7580
H	9.6157	7.4111	29.0049	N	8.2158	31.2191	16.2755	H	8.4564	14.7569	20.2065
H	7.9324	6.8995	28.5880	C	9.2496	30.5226	15.7345	H	8.1658	16.6765	18.6512
H	8.2084	8.4985	29.3797	C	16.0233	13.5623	21.5525	H	7.9952	29.8020	12.6455
C	9.5171	20.1077	15.6446	C	17.2032	13.3300	22.2708	H	6.0920	31.1381	13.5968
C	9.1730	21.1507	14.7616	C	17.1183	12.9383	23.6011	H	10.1622	30.4273	16.3316
C	8.9659	22.4706	15.2619	C	15.8553	12.8010	24.1850	H	18.1783	13.4653	21.7920
C	8.6157	23.4958	14.3742	N	14.6895	12.9367	23.4620	H	18.0248	12.7485	24.1953
C	8.4481	23.2072	13.0224	C	14.7905	13.2989	22.1566	H	13.8637	13.3794	21.5800
C	8.6251	21.9241	12.5126	C	5.9684	31.9607	16.2688	H	5.0010	32.1490	15.7788
C	8.9825	20.8785	13.3742	N	6.2136	32.1882	17.5401	H	4.9447	30.8463	19.4586
C	9.6731	20.3716	17.0203	C	5.1373	32.6911	18.3394	H	3.0793	31.7166	20.8788
C	9.4678	21.6907	17.5230	C	4.5829	31.8638	19.3318	H	3.1899	35.4617	18.6995
C	9.6181	21.9339	18.8944	C	3.5478	32.3366	20.1092	H	5.0453	34.5916	17.3036
C	9.9477	20.8848	19.7485	C	3.0463	33.6450	19.8897	H	2.1302	36.0645	20.7493
C	10.1356	19.5858	19.2854	C	3.5872	34.4589	18.8811	H	0.6714	35.2059	21.3846
C	9.9880	19.3102	17.9195	C	4.6215	33.9720	18.0994	H	0.9278	35.3283	19.6017
C	9.6827	18.7964	15.1557	O	2.0603	33.9721	20.7244	H	16.4635	12.4389	26.3015
C	9.4937	18.5218	13.7688	C	1.4257	35.2464	20.5738	H	13.4529	14.5159	27.5800
C	9.6501	17.2112	13.2991	C	15.6172	12.5774	25.6106	H	12.9275	14.2810	30.0135
C	9.9605	16.1916	14.1946	N	14.3547	12.5923	25.9752	H	14.2388	10.1532	29.8788
C	10.1346	16.4308	15.5552	C	14.0679	12.4484	27.3717	H	14.7292	10.3873	27.4591
C	9.9918	17.7321	16.0538	C	13.5755	13.5582	28.0807	H	12.8979	10.2160	31.8482
N	9.1128	22.7019	16.6294	C	13.2967	13.4361	29.4246	H	13.1459	11.4229	33.1711
N	9.1481	19.5710	12.9179	C	13.5299	12.1999	30.0785	H	14.5605	10.8611	32.2003
N	10.1330	18.0204	17.4112	C	14.0408	11.1003	29.3713	Fe	8.0582	31.9035	18.1406
C	8.8207	19.2689	11.5413	C	14.3197	11.2329	28.0202				

10. References

(1) Plajer, A. J.; Percastegui, E. G.; Santella, M.; Rizzuto, F. J.; Gan, Q.; Laursen, B. W.; Nitschke, J. R. Fluorometric recognition of nucleotides within a water-soluble tetrahedral capsule. *Angew. Chem. Int. Ed.* **2019**, *58*, 4200-4204.

(2) Hammershoj, P.; Sorensen, T. J.; Han, B. H.; Laursen, B. W. Base-assisted one-pot synthesis of N,N',N"-triaryltriazatriangulenium dyes: enhanced fluorescence efficiency by steric constraints. *J. Org. Chem.* **2012**, *77*, 5606-5612.

(3) Poveda, A.; Alonso, I.; Fernández-Ibáñez, M. Á. Experimental and computational studies on the mechanism of the Pd-catalyzed C(sp3)–H γ-arylation of amino acid derivatives assisted by the 2-pyridylsulfonyl group. *Chem. Sci.* **2014**, *5*, 3873-3882.

(4) Lavendomme, R.; Ronson, T. K.; Nitschke, J. R. Metal and organic templates together control the size of covalent macrocycles and cages. *J. Am. Chem. Soc.* **2019**, *141*, 12147-12158.

(5) Allan, D.; Nowell, H.; Barnett, S.; Warren, M.; Wilcox, A.; Christensen, J.; Saunders, L.; Peach, A.; Hooper, M.; Zaja, L.; Patel, S.; Cahill, L.; Marshall, R.; Trimnell, S.; Foster, A.; Bates, T.; Lay, S.; Williams, M.; Hathaway, P.; Winter, G.; Gerstel, M.; Wooley, R. A Novel Dual Air-Bearing Fixed-χ Diffractometer for Small-Molecule Single-Crystal X-ray Diffraction on Beamline I19 at Diamond Light Source. *Crystals* **2017**, *7*, 336.

(6) Winter, G. xia2: an expert system for macromolecular crystallography data reduction. *J. Appl. Crystallogr.* **2010**, *43*, 186-190.

(7) Winter, G.; Waterman, D. G.; Parkhurst, J. M.; Brewster, A. S.; Gildea, R. J.; Gerstel, M.; Fuentes-Montero, L.; Vollmar, M.; Michels-Clark, T.; Young, I. D.; Sauter, N. K.; Evans, G. DIALS: implementation and evaluation of a new integration package. *Acta Cryst.* **2018**, *D74*, 85-97.

(8) Farrugia, L. WinGX and ORTEP for Windows: an update. *J. Appl. Crystallogr.* **2012**, *45*, 849-854.

(9) Sheldrick, G. SHELXT - Integrated space-group and crystal-structure determination. *Acta. Cryst.* **2015**, *A71*, 3-8.

(10) Sheldrick, G. M. Crystal structure refinement with SHELXL. *Acta. Cryst.* **2015**, *C71*, 3-8.

(11) van der Sluis, P.; Spek, A. L. BYPASS: an effective method for the refinement of crystal structures containing disordered solvent regions. *Acta Cryst.* **1990**, *A46*, 194-201.

(12) Spek, A. L.: *PLATON: A Multipurpose Crystallographic Tool*; Utrecht University: Utrecht, The Netherlands, 2008.

(13) Kleywegt, G. J.; Jones, T. A. Detection, Delineation, Measurement and Display of Cavities in Macromolecular Structures. *Acta Cryst.* **1994**, *D50*, 178-185.

(14) Hristova, Y. R.; Smulders, M. M. J.; Clegg, J. K.; Breiner, B.; Nitschke, J. R. Selective anion binding by a "chameleon" capsule with a dynamically reconfigurable exterior. *Chem. Sci.* **2011**, *2*, 638-641.

(15) Stewart, J. J. P. Optimization of Parameters for Semiempirical Methods VI: More Modifications to the NDDO Approximations and Re-optimization of Parameters. *J. Mol. Mod.* **2013**, *19*, 1–32.

(16) MOPAC2016, James J. P. Stewart, Stewart Computational Chemistry, Colorado Springs, CO, USA, http://OpenMOPAC.net (2016).
